# *Hermetia illucens* (L.) (Diptera: Stratiomyidae) Odorant Binding Proteins and Their Interactions with Selected Volatile Organic Compounds: An In Silico Approach

**DOI:** 10.3390/insects12090814

**Published:** 2021-09-11

**Authors:** Carmen Scieuzo, Marisa Nardiello, Donatella Farina, Andrea Scala, Jonathan A. Cammack, Jeffery K. Tomberlin, Heiko Vogel, Rosanna Salvia, Krishna Persaud, Patrizia Falabella

**Affiliations:** 1 Department of Sciences, University of Basilicata, via dell’Ateneo Lucano 10, 85100 Potenza, Italy; carmen.scieuzo@unibas.it (C.S.); nardiellomarisa@gmail.com (M.N.); donatella.farina.92@gmail.com (D.F.); andreascala@inwind.it (A.S.); 2 Spinoff XFlies s.r.l, University of Basilicata, via dell’Ateneo Lucano 10, 85100 Potenza, Italy; 3 Department of Entomology, Texas A&M University, College Station, TX 77843, USA; jcammack_07@tamu.edu (J.A.C.); jktomberlin@tamu.edu (J.K.T.); 4 Department of Entomology, Max Planck Institute for Chemical Ecology, Hans-Knöll-Straße 8, D-07745 Jena, Germany; hvogel@ice.mpg.de; 5 Department of Chemical Engineering and Analytical Science, The University of Manchester, Manchester M13 9PL, UK

**Keywords:** black soldier fly, next generation sequencing, expression analysis, VOCs, OBPs, molecular docking

## Abstract

**Simple Summary:**

The black soldier fly (BSF) is a saprophagous insect that is an efficient bioconverter of organic waste because of its extreme voracity and fast larval development. Specific chemical molecules from decaying organic substances can influence BSF behaviour; in particular, several are likely attractants for BSF and are able to induce female oviposition and larval feeding. In insects, the perception of volatile organic compounds (VOCs) is based on a wide arsenal of chemoreception proteins. As a first step in understanding this process, an assessment of chemoreception genes belonging to the larval and adult stages of *Hermetia illucens* was conducted together with candidate VOCs that potentially regulate adult females searching for oviposition sites and phagostimulants for their larval progeny. The genes encoding several odorant binding proteins (OBPs) of interest were identified and three-dimensional models of these proteins were created, allowing a preliminary investigation of how different VOCs may interact with their binding sites. The present study provides a road map for further analysis and correlation among insect olfactory proteins and VOCs indicative of organic decomposition, starting from a computational approach to establish a reliable correlation between them.

**Abstract:**

The black soldier fly (BSF), *Hermetia illucens* (Diptera: Stratiomyidae), has considerable global interest due to its outstanding capacity in bioconverting organic waste to insect biomass, which can be used for livestock, poultry, and aquaculture feed. Mass production of this insect in colonies requires the development of methods concentrating oviposition in specific collection devices, while the mass production of larvae and disposing of waste may require substrates that are more palatable and more attractive to the insects. In insects, chemoreception plays an essential role throughout their life cycle, responding to an array of chemical, biological and environmental signals to locate and select food, mates, oviposition sites and avoid predators. To interpret these signals, insects use an arsenal of molecular components, including small proteins called odorant binding proteins (OBPs). Next generation sequencing was used to identify genes involved in chemoreception during the larval and adult stage of BSF, with particular attention to OBPs. The analysis of the de novo adult and larval transcriptome led to the identification of 27 and 31 OBPs for adults and larvae, respectively. Among these OBPs, 15 were common in larval and adult transcriptomes and the tertiary structures of 8 selected OBPs were modelled. In silico docking of ligands confirms the potential interaction with VOCs of interest. Starting from the information about the growth performance of *H. illucens* on different organic substrates from the agri-food sector, the present work demonstrates a possible correlation between a pool of selected VOCs, emitted by those substrates that are attractive for *H. illucens* females when searching for oviposition sites, as well as phagostimulants for larvae. The binding affinities between OBPs and selected ligands calculated by in silico modelling may indicate a correlation among OBPs, VOCs and behavioural preferences that will be the basis for further analysis.

## 1. Introduction

*Hermetia illucens* (L.) (Diptera: Stratiomyidae), commonly known as the black soldier fly (BSF), is attracted by decomposing organic matter to lay their eggs. These saprophagous insects are of economic importance since the larvae can be used to promote the biodegradation of a variety of organic waste. However, little is known about the chemosensory mechanisms associated with these insects or attractant chemicals that may govern the choice of substrates for oviposition. Here, we investigate volatile organic compounds (VOCs) associated with different diets, identifying those that may be implicated with the choice of the insect for certain substrates. We report the transcriptome analysis to identify chemosensory genes and propose a link between odorant binding proteins (OBPs) and VOCs based on in silico docking analysis of the binding sites of these proteins with a large range of VOCs.

These insects have a cosmopolitan distribution [1]. Because of the extreme voracity of the larvae, together with a brief period of larval development, they are considered efficient bioconverters of organic wastes, producing high quality biomass for use as livestock, poultry and aquaculture feed [2,3,4,5]. Because of their generalist nature, BSF larvae are able to develop on a variety of substrates, ranging from decomposing animal and vegetal resources to manure [6], food scrap waste [7], distiller grains [8], animal offal, kitchen waste and organic wastes from the agri-food chain [3,4]. The substrate can influence BSF development and the final nutrient composition of larvae, which are rich in proteins, lipids and minerals [9]. Larvae can consume twice their body weight on many substrates daily [10]. The diversity of substrates processed is higher than any other insect species, and the bioconversion process is more efficient than any other known dipteran species used for such purposes, due to the robust mouthparts and digestive enzymes [11,12]. While feeding, larvae can reduce dry matter by 50–80% and convert up to 20% into larval biomass within 14 days [2,13,14], also reducing the bacterial load typical of certain substrates (for example manure) [15,16], due to the production of antimicrobial peptides [17,18,19]. Once larvae have completed their feeding, they disperse from the substrate towards a dry site in order to complete the pupation process that lasts around two weeks [20]. After emergence, the adults mate, and females oviposit two days later near sites where decomposing organic matter is present [21].

Environmental factors, such as light, temperature and humidity, as well as molecules derived from decomposing organic matter can influence BSF behaviour [22,23,24]. The choice of oviposition sites is influenced by the odours associated with organic decomposition, but there has been little investigation thus far on the chemoreception mechanisms of this species.

VOCs emitted by fruits and vegetables (i.e., food substrates for BSF larvae) can vary depending on their composition (i.e., percent makeup of a substrate) [25]. Shifts in VOCs may impact adult BSF attraction and oviposition. Studies identifying these VOC attractants are needed to enhance colony maintenance efficiency in industrialized settings. Utilizing a low palatability substrate can inhibit oviposition and consequently the waste bioconversion process.

Most of these attractive VOCs are released by microbial species (fungi and bacteria) as side-products of their primary and secondary metabolism during the course of spoilage of organic matter [26,27,28]. The efficacy of organic matter biodegradation by BSF should be considered in the context of the insect’s ability to detect and be attracted to specific VOCs emitted by fruits and vegetables, which may also be considered as promising substrates for rearing this species [4]. Different species are known to be associated with different decomposing organic substrates and the VOCs in turn influence the choice of oviposition sites by insect adult females and/or the attractiveness for larvae and their bioconversion efficiency [29,30,31]. Many alcohols, aldehydes, aromatics, aliphatics, acids, ethers, esters, ketones, terpenoids and other compounds are released by substrate-specific microorganisms and therefore may be indicative of their presence. For instance, when apples are subjected to fungal attack, during storage for several months after harvest, typical compounds produced are 1-butanol, 1-hexanol, butanoic acid propyl ester, hexanoic acid ethyl ester, and butyl 2-methylbutanoate [32]. Grains often suffer from microbial contamination by bacteria and fungi during storage. Aldehydes and alcohols are predominant in oat grains [33], while 3-methylbutanal is the main compound identified from spent grain, where fungi are common [34].

For many insects, the detection of volatile and soluble chemicals has a key role to find food sources, identify oviposition sites, localize mates and avoid dangers [35]. Chemical perception in insects is known to be mediated by molecules belonging to the classes of olfactory, gustatory and ionotropic receptors, and soluble olfactory proteins, including OBPs and chemosensory proteins (CSPs) [36]. Particularly, OBPs and CSPs are involved in the transport of hydrophobic chemical compounds from the external environment to sensillar lymph and finally to the receptors located in the membrane of sensory neurons [37]. Insect OBPs have been identified in several species of different orders [38,39,40,41]. They are small proteins consisting of 130–150 amino acids (13–17 kDa) [36,42], characterized by the presence of a conserved pattern of six cysteines and three disulphide bridges, which limit the molecule flexibility but ensure a greater resistance to degradation [43]. OBPs can be divided into several categories, including classical OBPs (with six positional conserved cysteines, paired into three interlocked disulphide bridges, following a specific motif pattern C_1_-X_25-30_-C_2_-X_3_-C_3_-X_36-42_-C_4_-X_8-14_-C_5_-X_8_-C_6_) [44], plus-OBPs (with more than six cysteines) [45], minus OBPs (with less than six cysteines) [46,47] and atypical OBPs with more than eight cysteines [45,46,48].

Using a transcriptomic approach, we identified olfactory elements that may be involved in the chemoreception of attractive or repulsive VOCs. Combining transcriptomic data useful for selection of the most expressed OBPs and in vivo experimental tests to identify VOCs of interest allowed in silico analyses to estimate the proteomic functionality of individual OBPs. The binding sites of the selected OBPs were examined in detail by in silico docking of volatile ligands to give estimates of chemical affinities. Understanding the nature of VOCs emitted by different substrates colonized with BSF larvae, linked to the computational information on chemosensory proteins, will allow VOCs with the highest affinities to be identified, and in consequence, the most attractive compounds for adults and larvae, fostering the development of strategies to increase insect performance for waste bioconversion.

## 2. Materials and Methods

### 2.1. Insect Rearing

BSF larvae and adults, used for RNA extraction and sequencing, were reared in a colony maintained in the Laboratory of Insect Physiology and Molecular Biology at the University of Basilicata (Potenza, Italy). Larvae were reared on a standard Gainesville diet (30% alfalfa, 50% wheat bran, 20% corn meal) [49] at 70% moisture, under controlled conditions: temperature 27 ± 1.0 °C, relative humidity of 70% ± 5% and photoperiod of 12 L:12 D (light: dark, hours). Resulting pupae were transferred into a different room and metamorphosis into adults occurred under the same rearing conditions.

### 2.2. RNA Extraction from BSF Larvae and Adults

Total RNA, used for tissue-specific transcriptome sequencing, was extracted using liquid nitrogen and TRI-Reagent (Sigma-Aldrich, St. Louis, MO, USA), according to the manufacturer’s protocol, from 10 BSF larvae of second and fourth instar (5 larvae for each stadium), 10 adults (5 female and 5 male bodies, both without antennae), and 200 antennae excised from adult females and males, respectively. For the adult body and antennae, a mix of virgin and starved males and females, from pupal emerging to the 2nd day, was collected. A DNase (Turbo DNase, Ambion Austin, TX, USA) treatment was conducted to eliminate any contaminating DNA. The DNase enzyme was then removed, and the RNA was further purified using the RNeasy MinElute Clean-up Kit (Qiagen, Venlo, The Netherlands), following the manufacturer’s protocol, and eluted in 20 µL of RNAse free water (Ambion Austin, TX, USA). The integrity and purity of total RNA was determined by agarose (0.8%, *w*/*v*) gel electrophoresis, and RNA concentration was measured using a spectrophotometer (NanoDrop ND-1000).

### 2.3. RNA Sequencing and Transcriptome De Novo Assembly

Tissue-specific transcriptome sequencing of the RNA sample was performed with poly(A)+ enriched mRNA (New England Biolabs, Ipswich, MA, USA) fragmented to an average of 240 nucleotides. RNA sequencing was performed on an Illumina HiSeq 2500 Genome Analyzer platform, using standard TruSeq procedures at the Max Planck Genome Center (Jena, Germany) (http://mpgc.mpipz.mpg.de/home/, accessed in June 2018), generating ~42 Mio paired-end (2 × 100 bp) reads for the tissue samples. Sequencing quality assessments, trimming of the Illumina reads using standard settings and the de novo transcriptome assemblies were conducted using CLC Genomics Workbench v9 (http://www.clcbio.com, accessed in June 2018). All obtained sequences (contigs) were used as queries for a blastx search [50] in the National Center for Biotechnology Information (NCBI) non-redundant (nr) database, considering all hits with an e-value <1 × 10^−5^. The transcriptome was annotated using BLAST, Gene Ontology and InterProScan searches using BLAST2GO PRO 4.1 (www.blast2go.de, accessed in September 2018) [51]. To optimize annotation of the obtained data, GO slim, a subset of GO terms that provides a higher level of annotations and allows a more global view of the result, was used. Digital gene expression analysis was performed using CLC Genomics workbench v9 (http://www.clcbio.com, accessed in September 2018) to generate BAM (mapping) files and QSeq Software (DNAStar, Inc., Madison, WI, USA, accessed in September 2018) to remap the Illumina reads onto the reference transcriptome, and finally, by counting the sequences to estimate expression levels, using previously described parameters for read-mapping and normalization. In particular, expression levels of each contig was calculated based on the fragments per kilobase per million mapped reads (FPKM) method, using the formula: FPKM (A) ¼ (10,00,000_C_1000)/(N_L), where FPKM (A) is the abundance of gene A, C is the number of reads that uniquely aligned to gene A, N is the total number of reads that uniquely aligned to all genes and L is the number of bases in gene A [52].

The six reading frames of the 78,763 nucleotide sequences of adult transcriptome and the 25,133 nucleotide sequences of larval transcriptome were translated into the corresponding amino acid sequences by SEQtools software (http://www.seqtools.dk/, accessed in December 2018).

### 2.4. Identification of Chemosensory Genes

The identification of chemosensory genes, including sensory neuron membrane proteins (SNMPs), chemosensory proteins (CSPs), odorant receptors (ORs), gustatory receptors (GRs), glutamate receptors (GluR), chemosensory proteins (CSPs) and odorant binding proteins (OBPs) for BSF larval and adult transcriptomes was performed. All candidate proteins were manually checked with the BLAST/blastx program from the National Center for Biotechnology Information (NCBI), considering the query cover, percentage of identity and e-value. Query cover is the percentage of the length of sequence of interest that align with sequences in database; identity is the percentage of nucleotides/amino acids that match in the alignment, the e-value represents the quality of the alignment, Considering both the query cover and the percentage of identity [53]. Concerning putative OBPs sequences, each contig was translated in the respective amino acid sequence with Translate Tool software, by ExPASy (https://web.expasy.org/translate/, accessed in May 2020), searching for the right frame and the completeness at 5′ and 3′ ends. Then, the correct amino acid sequence was analysed to identify the signal peptide (through the SignalP 5.0 software (http://www.cbs.dtu.dk/services/SignalP/, accessed in May 2020) and the cysteine pattern.

### 2.5. Differential Expression of OBP Genes in Adult BSF and Identification of Common OBPs in Larval and Adult Transcriptome

In order to show OBP genes differentially expressed in female and male bodies and antennae, heat maps of these genes were generated. The map was based on log2-transformed FPKM values shown in the gradient heat map, and to identify the common OBPs in larval and adult transcriptome, the nucleotide sequences of these proteins were translated using Expasy-translate tool software (https://web.expasy.org/translate/, accessed in May 2020). The corresponding protein sequences were aligned using the BLAST/blastp program from NCBI (https://blast.ncbi.nlm.nih.gov/Blast.cgi?PAGE=Proteins, Rockville PikeBethesda MD, USA, accessed in May 2020). OBP protein alignments were generated with MAFFT v7.388 implemented in Geneious Prime (a bioinformatics software package), using the Fast Fourier Transform algorithm and the normalized similarity matrix (FFT-NS-i × 1000) algorithm, BLOcks SUbstitution Matrix (BLOSUM62) scoring matrix, a gap open penalty of 1.53 and an offset value of 0.123. Approximately maximum-likelihood phylogenetic trees from alignments of *H. illucens* OBP protein sequences were generated with FastTree v2.2.11 implemented in Geneious Prime, using the Whelan-And-Goldman 2001 model0, optimized Gamma20 likelihood and using pseudocounts.

### 2.6. Volatile Organic Compound (VOC) Sampling

On the basis of previous work, in which the growth performance of BSF reared on different substrates from the agri-food chain (apple, banana and spent grain alone or mixtures) was analysed [9], VOC sampling was conducted at the beginning (prefeeding) and at the end of the process (postfeeding) of BSF larvae feeding on these substrates. The end of the process was determined when a decrease in larval weight was registered, suggesting the beginning of the prepupal stage [9,54,55].

Each rearing tray (n = 3 per substrate), containing substrate and larvae, was covered tightly with aluminium foil to entrap the air over the substrate. A 14.6 cm glass Labcraft Pasteur pipet (Curtin Matheson Scientific, Inc., Morris Plains, NJ, USA) filled with 0.75 g Black Diamond activated carbon (Marineland, Cincinnati, OH, USA) was placed in a hole in the aluminium foil to purify incoming air. A volatile trap packed with 30.0 mg of Hayesep^®^ Q porous polymer (Volatile Assay Systems, Rensselaer, NY, USA) was placed in another hole in the aluminium foil on the opposite side of the tray to collect VOCs. The volatile traps were attached to an AC110 V, 60 Hz, oil free vacuum pump (Rocker 300, Rocker Scientific Co., Ltd., New Taipei City, Taiwan) through a flow meter (Dwyer Instruments, Inc., Michigan City, IN, USA). Volatiles were pulled from each tray at a rate of 1.0 L·min^−1^ for 1 h under laboratory conditions. The control was a pan without larvae kept under the same conditions to assess the impact of larvae on the VOC profile.

### 2.7. GC/MS Analysis

In order to analyse relevant compounds from decomposing substrates, VOCs were eluted from the Hayesep^®^ Q using 150 µL of dichloromethane (DCM) (Thermo Fisher Scientific, Waltham, MA, USA) and ultra-high purity nitrogen into 9.0 mm 300 μL insert (Thermo Fisher Scientific, Waltham, MA, USA) within a 1.5 mL SureStop™ GC vial (Thermo Fisher Scientific, Waltham, MA, USA). An aliquot of 5 µL of n-Octane (Sigma-Aldrich, St. Louis, MO, USA) was added as internal standard (80 ng/µL). GC/MS analysis was performed at the Geochemical and Environmental Research Group (G.E.R.G.) at the Texas A&M University, College Station, using a Hewlett-Packard 6890 gas chromatograph with a Hewlett-Packard 5973 mass selective detector (Hewlett-Packard Company, Palo Alto, CA, USA). The column used was a fused silica DB-5MS capillary column (30.00 m × 0.25 mm ID, 0.50 μm film thickness) (Agilent Technologies, Santa Clara, CA, USA). Injections of 1 μL were performed in split mode with an injection temperature of 250 °C. Zero-grade helium was used as the carrier gas at a flow rate of 1.2 mL·min^−1^. A preliminary database of 100 VOCs based on literature data of VOCs found in our substrates (apple, banana and spent grain) and VOCs coming from different sources of decaying organic matter was built (Supplementary Table S1). VOCs were identified by comparing their mass spectra fragmentation patterns with those stored in the NIST05 mass spectra library, Kovats indices, and chemical standards. Differences in VOCs profile between treatments (prefeeding vs. postfeeding) and substrates were calculated using PERMANOVA, non-metric multidimensional scaling (NMDS), multiple response permutation procedure (MRPP), and indicator species analysis (ISA) in the statistical package R (R Core Team, 2010). In response to the BSF feeding, the differential production of a subset of 55 VOCs, several known to be produced by microbes or important in other decomposition systems, was analysed using a *t* test in JMP^®^ Pro 15 (JMP 2019) [56].

### 2.8. Ab Initio Modelling of OBPs and Virtual Ligand Screening

Due to the lack of X-ray crystal or nuclear magnetic resonance (NMR) structures, the tertiary structures of selected OBPs were modelled *ab initio* using the I-TASSER web server [57] and saved in a .pdb format (accessed in April 2021). Seven OBPs with 100% of query cover and 100% of identity among the common sequences between larvae and adults were selected to study the possible interaction with VOCs. An additional OBP (C31956) with 100% of query cover and 64.19% of identity between larvae and adults was selected since it was the most expressed OBPs in male and female antennae. Based on the amino acid sequence deprived of signal peptide, the server first tried to retrieve the initial template from the PDB library by LOMETS, a locally installed meta-threading approach. Then, the continuous fragments excised from the PDB templates were reassembled into full-length models, chosen on the basis of the highest C-score. After the model selection, the quality of the obtained models was further evaluated using the molecular graphics software PyMOL Version 2.0 (Schrödinger, LLC) (accessed in May 2021) [58]. The molecular conformations of all tested VOCs were constructed with MarvinSketch software (ChemAxon’s Chemicalize platform, http://www.chemaxon.com/products/marvin/marvinsketch/) and downloaded in .mol2 format (accessed in June 2021). To predict the possible binding modes of different VOCs to OBPs and the best interaction with the strongest affinity or lowest ΔG (kcal/mol), a molecular docking simulation with SwissDock algorithm [59] was performed following default protocols. The resulting docking predictions were viewed and analysed using the SwissDock server plugin in UCSF Chimera X software [60] and energetic evaluations of different docked complexes were implemented with a ClusterRank algorithm (accessed in June 2021). The Computed Atlas of Surface Topography of proteins (CASTp) web server [61] provided useful data to locate and measure the area and volume of all the possible OBP pockets involved in the binding of specific VOCs (accessed in June 2021).

## 3. Results

### 3.1. Candidate Chemosensory Genes in BSF Adult and Larval Transcriptomes

In order to identify genes that are involved in BSF chemoreception during the larval and adult stages, next generation sequencing (NGS) was performed. Sequencing and de novo assembly of the combined transcriptome (hereafter defined as “adult transcriptome”) derived from antennae and whole bodies of BSF adult females and males resulted in 78,763 contigs, with a maximum contig length of 16,723 bp.

Each identified contig was functionally annotated using the Blast2GO software (http://www.blast2go.org accessed in June 2021). The candidate chemosensory genes were further manually checked with BLAST software in order to confirm the Blast2GO results, allowing the identification in the adult transcriptome of 47 putative odorant binding proteins (Supplementary Table S2a, Supplementary Figure S1a), 127 putative odorant receptors (27 of them putative ionotropic receptors) (Supplementary Table S2b, Supplementary Figure S1b,b′), 25 putative gustatory receptors (Supplementary Table S2c, Supplementary Figure S1c), 24 putative glutamate receptors (Supplementary Table S2d, Supplementary Figure S1d), 4 putative chemosensory proteins (Supplementary Table S2e, Supplementary Figure S1e) and 2 putative sensory neuron membrane proteins (Supplementary Table S2f, Supplementary Figure S1f).

The larval transcriptome comprised 25,128 contigs, with a maximum contig length of 23,709 bp. Analysis using the Blast2GO software led to the identification of 36 putative odorant binding proteins (Supplementary Table S3a, Supplementary Figure S2a), 1 putative ionotropic receptor (Supplementary Table S3b, Supplementary Figure S2b), 1 putative gustatory receptor (Supplementary Table S3c, Supplementary Figure S2c), 5 putative glutamate receptors (Supplementary Table S3d, Supplementary Figure S2d), 1 putative chemosensory protein (Supplementary Table S3e, Supplementary Figure S2e) and 1 putative sensory neuron membrane protein (Supplementary Table S3f, Supplementary Figure S2f).

### 3.2. Differential Expression of OBP in BSF Adults and Larvae

**Figure 1:**
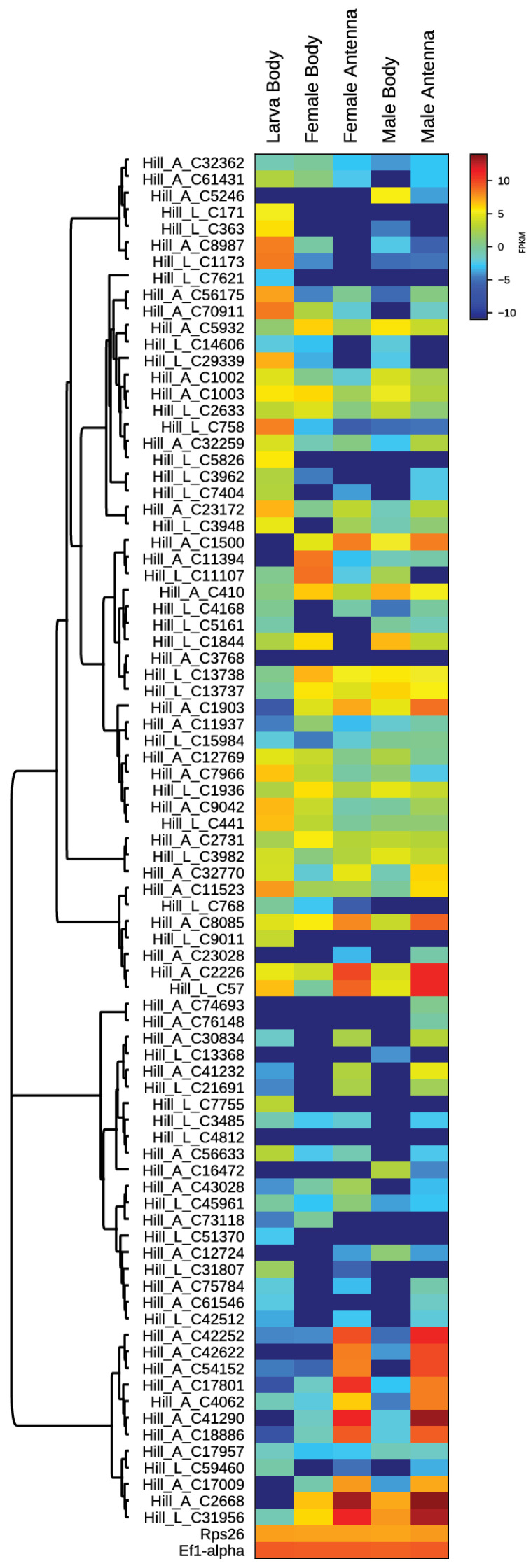
Heat map showing differences in the expression of OBPs between BSF adult male and female individuals (antennae and whole bodies) and in BSF larvae. The map is based on log2- transformed FPKM values shown in the gradient heat map (blue represents low-expressed genes and red represents high-expressed genes). The housekeeping genes 40 S ribosomal protein 26 (Rps26) and elongation factor 1-alpha (EF1-alpha) are used for normalization and are shown to confirm the uniform expression of these control genes across samples.

Using the transcriptome and RNAseq mapping data, it was also possible to evaluate transcript levels of OBP genes expressed in female and male bodies and antennae and the transcript levels of OBP genes expressed in BSF larvae. The different expression levels are shown in the heat map, based on log2-transformed FPKM values (Figure 1).

Sequence analysis, performed by BLAST software and searching for sequence completeness at the 5′ and 3′ ends, presence of signal peptide and conserved cysteine pattern, led to the identification of 27 adult and 31 larval OBPs (Supplementary Tables S2a and S3a). From the transcriptome of adults, 22 Classical OBPs, 3 Plus OBPs, 1 Atypical OBP and 1 Minus OBP were identified; from the transcriptome of larvae 22 Classical OBPs, 2 Plus OBPs and 5 Atypical OBPs were identified (Supplementary Tables S4 and S5). Moreover, we detected 6 OBPs more frequently expressed in females and 7 in males, 15 OBPs more frequently expressed in the antennae, and 10 OBPs more frequently expressed in the bodies. All the other OBPs have similar expression levels in all the analysed samples.

### 3.3. Identification of Common OBPs in Larval and Adult Transcriptomes

Complete OBP sequences from larval and adult transcriptomes were compared using BLASTp (National Center for Biotechnology informatio-NCBI, https://blast.ncbi.nlm.nih.gov/Blast.cgi?PAGE=Proteins accessed in June 2021) and the analysis provided significant sequence similarity (99–100% of query cover and 95–100% identity) for 15 OBPs identified in both transcriptomes (Table 1), while the remaining larval OBPs showed a lower similarity compared to adult OBPs (Supplementary Table S5). Information on further common OBPs between adults and larvae with lower identity value (higher than 50%) are reported in Supplementary Figure S1. Comparing expression level of common larval and adult OBPs (considering FPKM reported in Supplementary Table S5) several have higher expression levels in larvae (contigs 768, 1173, 2633, 3948, 3982, 59,460). On the contrary, several have a higher expression level compared directly to certain samples: the contigs 21,691 and 13,368 compared to male and female body without antennae; 9011 compared to male body without antennae; 1844 compared to female antennae; 45,961 and 11,107 compared to female and male antennae. Contig 57 has similar expression compared to female and male antennae, while contig 13,738 has the lowest expression compared to all other samples.

### 3.4. Identification of Volatile Organic Compounds

**Figure 2:**
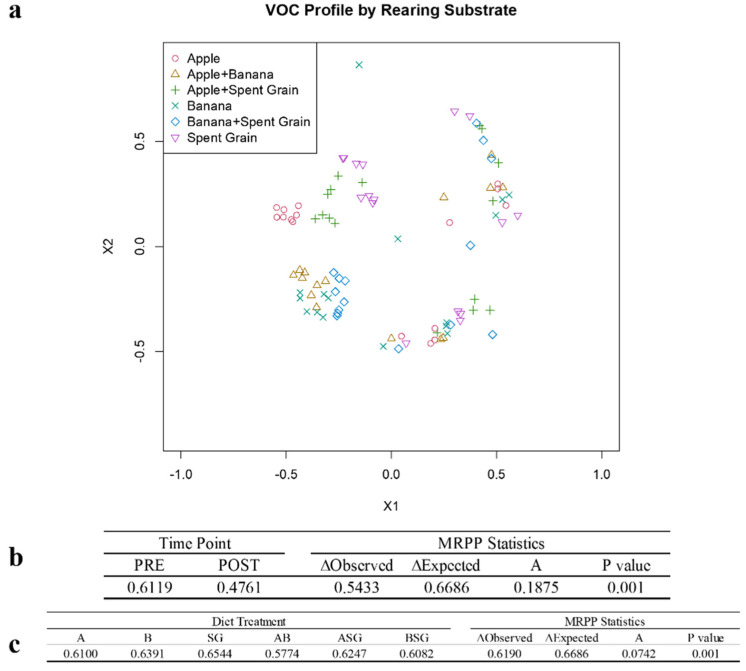
Identification of VOCs: (**a**) Nonmetric multidimensional scaling (NMDS) ordination of the overall VOC profiles for all six diet treatments, before and after feeding by BSF larvae. The vertical black line on the graph represents the separation in VOC profiles between the two sampling time points; (**b**) Bray–Curtis dissimilarity values (∆) and MRPP statistics for the overall VOC profile for each sampling time point: PRE (before) and POST (after) feeding by BSF larvae, for all six diet treatments combined; (**c**) Bray–Curtis dissimilarity values (∆) and MRPP statistics for each diet treatment. A, apple; B, banana; SG, spent grain; AB, apple and banana; ASG, apple and spent grain; BSG, banana and spent grain.

Decomposition processes were the focus of this investigation as the associated VOCs may be attractive for *H. illucens* in the induction of the oviposition phase and the subsequent larval feeding. In the analysed digested substrates, a total of 55 VOCs was identified in different amounts via GC/MS. These VOCs comprised a variety of compound classes, including aldehydes, alcohols, esters, terpenes and ketones, and they were distributed in different proportions in the six analysed diets (apple, banana, and spent grain, individually or in 1:1 mixtures). Other VOCs commonly associated with decomposing organic materials, included in the database specifically built for the analysis (Supplementary Table S6), were not produced by any of the six diet treatments. Sample time point (prefeeding vs. postfeeding) (F_1,87_ = 57.6, *p* < 0.001) and diet treatment (F_5,87_ = 4.273, *p* < 0.001) had a significant effect on the overall VOC profile generated by a given sample (Figure 2).

Among the VOCs targeted as standard compounds indicative of organic decomposition, 33 VOCs were identified as indicators of the substrates prior to larval feeding and 11 VOCs were identified as indicators of the substrates after feeding (Table 2). The remaining 11 VOCs did not show statistically significant differences between pre- and post-feeding phases.

A total of 20 VOCs was identified as indicators of four of the six different diet substrates, apple, banana, spent grain, and apple and banana (Table 3).

Additionally, we focused on 26 VOCs of interest (those known from other decomposition systems, or known to have impacts on insect behaviour, or known to be produced by microbes), in response to BSF larvae feeding, as these VOCs may be capable of uniquely typing the phases of organic degradation in different food matrices (Supplementary Table S7). Twenty-five VOCs were differentially produced across the six diet treatments in response to larval feeding. Most of them decreased in concentration: particularly, *2*-methyl-butanal, n-propyl acetate and acetic acid, and butyl ester were significantly lower in all diet treatments. Styrene was the only compound to significantly increase in all diet treatments, while benzaldehyde, also commonly associated with decomposing organic matter, was not affected by BSF feeding (Table 4).

### 3.5. Molecular Modelling and Virtual Docking of OBPs

**Figure 3:**
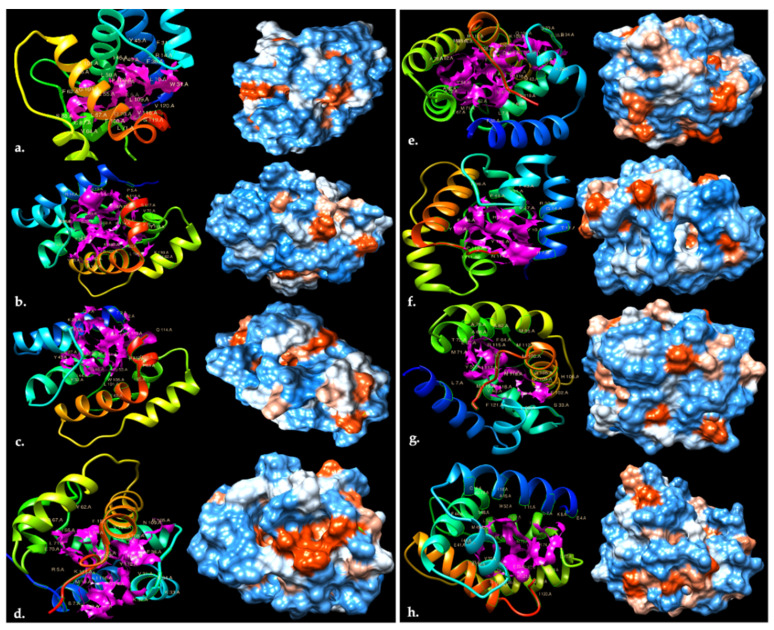
Three-dimensional OBPs structures modelled with UCSF Chimera software and provided by the I-TASSER server. (**a**) OBP_C57 (**b**) OBP_C1173 (**c**) OBP_C2633 (**d**) OBP_C11107 (**e**) OBP_C13368 (**f**) OBP_C13738 (**g**) OBP_C21691 (**h**) OBP_C31956. Left: ribbon diagram with a pink coloration indicative of the specific main pocket surface involved in ligand binding. Right: the Kyte–Doolittle scale shows the hydrophobic surface with colours ranging from blue for the most hydrophilic amino acidic residues to white and orange/red for the most hydrophobic ones.

Starting from the open reading frame (ORF) and following the comparison between larval and adult OBPs and considering several parameters in terms of 5′- and 3′-end completeness, presence of signal peptide and six-cysteine pattern (Supplementary Table S4), 8 common OBPs between *H. illucens* larvae and adults were eligible to be modelled ab initio with I-TASSER server (Figure 3). The selected OBPs consisted of six α-helices, held together by three pairs of disulphide bridges according to a conserved pattern; the hydrophilic residues were mostly present on the surface and a large hydrophobic cavity, with the possibility to accommodate various ligands, and showed a channel with a distinct entry mouth and specific residues involved in the binding activity. The CASTp server was used to identify all potential binding pockets within the OBPs and amino acids directly involved in binding activity, mainly hydrophobic in nature (Table 5). In order to better understand the binding events between OBPs and VOCs indicative of organic decomposition, molecular docking studies with SwissDock web service were preliminarily performed in silico. The energetic evaluations (free binding energy, ΔG) of the protein–ligand complexes were calculated and the lowest ΔG values were used to estimate the amount that the ligands were able to fully penetrate the binding pocket, since the lower the ΔG value the stronger the interaction, reflecting the affinity between VOCs and OBPs (Table 6). These energetic complexes are the sum of several weak electrostatic interactions, as electrostatic and van der Waals forces, hydrophobic interactions, hydrogen bonds and other noncovalent bonds (π-stacking or cation-π interaction). After docking of desired ligands against the protein binding sites, data were analysed using SwissDock plugin UCSF Chimera. Selected common OBPs between *H. illucens* larvae and adults showed higher binding affinities to all the selected ligands, with the exception of alpha- and beta-pinene. These two ligands are not directly involved in the interaction with OBPs because they do not have the right distance to the atoms located in the binding site, with a low contact surface.

## 4. Discussion

Scientific and economic interest on *H. illucens* is increasing due to its ability to bioconvert organic waste and use larvae for feed and food (in certain countries). Here, we provide preliminary information on the most attractive VOCs and on proteins involved in chemoreception. This opens the way for further studies and insights that can improve the bioconversion performance of this insect at industrial scale. Studies on *H. illucens* have historically focused on the behaviour of this insect in nature, the breeding physiology, bioconversion of organic wastes, and the larval biomass composition [9,62,63]. However, little is known about the molecular mechanisms and the specific volatile organic compounds (VOCs) involved in its behavioural preferences and developmental processes.

A recent study investigated different organic wastes (fruit wastes, household food wastes, chicken/pig/dairy manure) as oviposition sites for wild flies; eggs were only deposited on fruit wastes [64]. The explanation may be prior exposure to this waste type, as the urban site in which the experiment was conducted (i.e., university campus) was not surrounded by animal farms; however, this does not explain the lack of a response to household food wastes, which will presumably be abundant in trash collection locations in the environment. BSF females likely search for the most abundant food source for their progeny, to increase their chances of survival but the exact molecular mechanism responsible for this behaviour is unknown. Nyakeri et al. [65] demonstrated that manure, fish, fruits and frass attract BSF larvae and, in contrast to the Sripontan et al. [64] study, manure was the most attractive substrate. These seemingly contradictory findings can be potentially explained through an in-depth analysis of these organic matter at a more refined level, highlighting the importance of olfactory perception in BSF and how different life stages can be influenced by their environment. It is also important to underline the amount in which all stages are linked to each other: attractive compounds perceived during the larval stages can influence the adult stage and the inclination to search for different oviposition sites, as previously demonstrated in other Diptera [62]. For this reason, many and more detailed studies regarding the olfactory system are required to better understand which VOCs are the most attractive for BSF adults and larvae. OBPs have an important role in BSF females searching for suitable oviposition sites, for this reason, all genes involved in BSF olfaction were identified, with particular attention paid to OBPs. The analysis of OBP gene expression patterns in two different tissues (antennae and whole body), is helpful for clarifying their physiological function. In general, gene expression analyses revealed 31 putative OBPs expressed in adult antennae, suggesting that the BSF OBP genes identified in the current study may play an important role in the insect olfaction. Different expression profiles of OBPs in female and male antennae suggest different functions: female antennae OBPs may be involved in searching for the most suitable oviposition sites, rich in protein sources for egg production and consequently larval feeding, while male antennae-specific OBPs may be involved in sex pheromone detection and sexual attraction [66,67,68,69]. Several OBPs are expressed equally in female and male antennae, and this can be explained by simultaneous research for the specific area of mating by male and female, and at the same time, the search of the best oviposition sites by females. During mating, males locate a lekking area that is essential since females do not mate if there is not a territory with specific characteristics [62]. Generally, lekking areas are zones of vegetation near decomposing organic matter; in this way, males firstly compete to attract females, contemporary females attract males, and after the mating ritual, females can oviposit near decaying organic matter [70]. For all these reasons, we hypothesise that BSF males can also be attracted to decaying areas in order to mate and allow subsequent oviposition by females on the larval feeding substrates. 

In most insect species, OBPs are highly expressed in the antennae and associated with odour perception. However, among the OBPs identified in adults, 10 are expressed at higher levels in the female/male body, as reported for other insect species, such as in the aphids *Megoura viciae* (Buckton) [71] and *Acyrthosiphon pisum* (Harris) [72], the lepidopteran *Agrotis ipsilon* (Hufnagel) [73] and the hymenopterans *Polistes dominula* (Christ) [74] and *Sclerodermus* sp. [75]. Although the specific functions of several OBPs are still unknown, results from Sun et al. [76] suggest the possibility that the complexity of the insect OBP repertoire may have functions other than odorant transport in the lymph of olfactory sensilla on the antennae because an increasing number of OBPs have been found in other parts of the body. Many OBPs are expressed in the labellum, leg and taste organs in fruit flies, and influence their host-plant preferences [77,78] in sensilla, where they may be related to the olfactory and gustatory receptors [43,79,80], in the larval gut of tsetse (*Glossina* spp.) related to immune system development [81], venom glands of wasps [82,83] and reproductive organs of male mosquitoes [84,85]. The comparison between putative OBP genes in BSF adult and larvae transcriptomes showed 15 common OBPs. Thus, it is possible to assess that a group of the common OBPs share a similar expression pattern across these developmental stages, indicating that these OBPs may be involved in the perception of the same or similar VOCs with different functions: the presence of decomposing organic substrates may represent simultaneously a feeding stimulus for larvae and an egg-laying site for females. Our data on VOCs show clear differences between substrates and between colonized and uncolonized substrates and can help provide insights into which compounds adult female BSF use to identify suitable oviposition sites. The reduction in the number of compounds collected from substrates after being fed upon by BSF larvae is likely due to the vast array of antimicrobial peptides and enzymes the larvae produce and utilize while consuming decomposing resources [11,86,87] and subsequent impact on the microbes that are responsible for producing many of the VOCs collected. For example, butanoic acid butyl ester, and acetic acid methyl ester are produced respectively by *Mucor piriformis* and *Botrytis cinerea* during apple decomposition [32]. *Limonene* is abundant in fruits such as mango and nectarines, colonized by *Colletotrichum gloeosporioides*, while α-pinene, β-pinene and styrene are significantly higher in the presence of *Lasiodiplodia theobromae* [88]; *styrene* is also produced by *Penicillium* and repels pine weevils *Hylobius abietis* (L.) from Scots pine twigs [89]. Ragaert et al. [90] investigated the metabolic activity of yeast on strawberries, demonstrating the presence of 2-methyl-1-butanol, 3-methyl-1-butanol, 1-hexanol produced by *Debaryomyces melissophilus* and *Rhodotorula glutinis*, and isopropyl acetate as a secondary product of *Cryptococcus laurentii* activity. Additionally, previous studies have shown that BSF larvae are able to reduce microbial populations in waste streams [13,16,20,91]. Insect-microbe interactions are fundamental for ecosystem function and may help elucidate the mechanisms regulating subsequent insect attraction and colonization.

Among all analysed VOCs, 2-methyl-butanal production was significantly lower in all diet treatments after being fed upon by BSF larvae. This compound is produced by numerous bacteria and fungi [92,93,94]; *Staphylococcus* sp. produce *2*-methyl butanal during exponential growth, and adult female BSF may be using this compound as a cue to locate oviposition sites. This hypothesis is supported by the fact that Zheng et al. [31] found that BSF oviposited in response to the presence of *Staphylococcus* sp. isolated from larvae of the blow fly *Chrysomya rufifacies* (Macquart) (Diptera: Calliphoridae). The reduction of *2*-methyl-butanal may be the result of BSF larval activity negatively impacting *Staphylococcus* sp., as previously demonstrated with other bacteria [13,15,16,91]. The compounds n-propyl acetate and acetic acid, butyl ester decreased significantly in all diet treatments in response to the BSF larval feeding. Both compounds are produced by fungi and bacteria, but to date, nothing is known about the effect that these compounds may have on insects. *Styrene* is the only compound to increase significantly in all diet treatments after being fed upon by BSF larvae. This compound is produced by numerous bacteria and fungi [92,95] and is an indicator of fish spoilage [96]. The increase in production may be a cue to female BSF that a given resource is suitable for offspring development or may have the opposite effect as an indicator that a substrate is already colonized by other organisms or too decomposed. Benzaldehyde is the only compound further investigated that was not differentially produced in response to feeding by BSF larvae and it is produced by numerous bacteria and fungi [92,93,94]. Benzaldehyde has both insecticidal and antibacterial properties [97]. The lack of a decrease in production coupled with these properties suggest that the microbes responsible for producing benzaldehyde are well suited to compete with other microbes and BSF larvae for access to these resources. Production of the compound *3-methyl butanal* decreased only in the BSG (banana and spent grain) treatment after BSF larval feeding. This compound is produced by numerous bacteria and fungi and has been identified in numerous carrion sources [98,99,100]. Of the identified VOCs, 3-methyl-butanal is attractive to the dipterans *Anastrepha ludens* (Loew) (Tephritidae) and *Anopheles gambiae* Giles (Culicidae) [93] and has been incorporated into an attractant blend for trapping and management of numerous fly species [101]. The 3-methyl butanal is also known to be a compound commonly present in oat grains, and in particular, represents a chemical stimulus that determines the attractiveness towards spent grain [102] and greater palatability, supported by fast larval utilization of this substrate alone or in mixtures [9]. Based on previous work, where BSF growth performance on apple, banana and spent grain (alone or mixed) was analysed, substrates containing spent grain allowed higher critical weight gains in a shorter amount of time compared to other substrates, with the highest rate of bioconversion. The high performance of BSF larvae on spent grain are likely related to the nutritional properties (i.e., a more balanced mixture of nutrients when mixed with apple or banana), but also to the perception of specific VOCs such as 3-methyl butanal.

Odorant binding proteins (OBPs) play a role in insect chemoreception, such as larvae searching for or accepting food sources and in adults for choosing mating partners and localising oviposition sites. However, there is no specific information about the correlation between attractants, BSF behaviour and its chemoreception system. The large number of identified OBPs through the de novo transcriptome analysis of both adults and larvae is an indication for several different functions of chemoreception genes between the two stages of the BSF life cycle. The adult transcriptome contains a larger number of each chemoreception element than the larval one. In particular, the high number of ORs in adults is remarkable; the number of BSF OR genes are two-fold higher compared to *Musca domestica*, which has the second largest number of these genes among Diptera [103]. ORs are potential BSF-specific pheromone receptors and may be involved in BSF-specific recognition of environmental cues or mating and social behaviour [103]. ORs play a central role in the chemosensory signal transduction process, facilitating the conversion of the chemical message to an electrical signal. However, it has been shown that the perception of chemicals is not only dependent on ORs, but also requires the contribution of OBPs that are the first and main proteins involved in olfactory perception and can be highly selective and specific towards chemicals such as VOCs [104]. For predictions about OBPs biological features, computational biology was applied to speed up a preliminary virtual screening. In silico modelling of the docking between target (OBP) and ligands (VOCs), gives understanding of the OBP-VOC interactions. Detailed energy calculations of ligand-target docking may give an idea of the interaction mode of OBPs with different chemical classes of VOCs. From the in silico prediction, all the compounds seem to be able to bind the selected OBPs except for alpha- and beta-pinene, indicating a broad spectrum of selectivity. All the analysed compounds, although with a different binding affinity, do not seem to differ between all the 3D-analysed OBPs. Among the OBPs investigated, there was not considerable difference in predicted energies of interactions to the individual ligands tested, indicating similarities in the binding pockets. The most negative free energy values obtained seem to demonstrate a lack of selectivity towards different chemical classes, such that the OBPs are able to bind a large range of VOCs. However molecular models are limited by assumptions about the degrees of freedom and the accuracy by which the tertiary structure can be modelled; the predictions all referred to the main binding pocket, not considering different ways of interaction for each OBPs. However, these data are indicative of the binding of OBPs to potential ligands, starting from the structural conformation. The ecology of *H. illucens* larvae, which live in environments saturated with odours associated with organic decomposition, provide a rational explanation about the similar energy values obtained for all the OBPs with the same ligand. Larvae born from eggs laid in decomposition sites carefully chosen by adults may not need to develop an extremely specific and selective arsenal of OBPs because the progeny are already located in sites suitable for their survival, rich in different odours, without needing to move in the search of food. The identification of VOCs representative of specific sources, phases or processes of organic degradation, along with olfactory proteins involved in BSF chemoreception, provide a starting point for further investigation of the larval and adult BSF responses to these compounds, to describe the molecular interactions between relevant VOCs and OBPs. 

## 5. Conclusions

The identification of 55 VOCs characteristic of specific sources and processes of organic degradation, along with olfactory proteins involved in BSF chemoreception (27 and 31 OBPs for adults and larvae, respectively, 15 of them in common between the two stages) and in silico prediction OBPs–VOCs binding, provide a starting point for further investigation of the larval and adult BSF responses to these compounds and deepen and describe the molecular interactions between relevant VOCs and OBPs. Specific VOCs attractive to females are good indicators of larval progeny resources, and as a consequence, may be used as additives on substrates that are not normally attractive, thus stimulating oviposition. In this way, the larval bioconversion capacity may be optimized on different waste streams ranging from farming and agricultural processes, zootechnical, urban and agri-food industrial wastes that are difficult to valorise through the addition of specific VOCs that stimulate larval feeding. Future work on these identified compounds should focus on those that are microbial in origin, as many such compounds have been shown to play a role in the interaction between insects, microbes, and the decomposing resources on which these two groups of organisms feed. Moreover, the identification of putative OBP genes, differentially expressed in BSF larvae and in males and females by transcriptome sequencing, can help in unravelling molecular mechanisms of chemoreception. The ligand binding interactions between these OBPs and relevant VOCs are under investigation, and this will help further understanding of the chemoreception mechanisms associated with *H. illucens* adults and larvae.

## Figures and Tables

**Table 1:** Comparison between larval and adult OBPs. Complete OBP sequences from larvae (L) and adult (A) transcriptomes were compared using the Basic Local Alignment Search Tool Protein (BLASTp). Query cover, e-value and identity parameters were reported. The table shows the alignments with a query cover of 100% and an identity greater than or equal to 95%.

Larval Contig vs. Adult Contig	Query Cover	e-Value	Identity
L21961 A41232	100%	7 × 10^−104^	99.29%
L13368 A30834	100%	1 × 10^−104^	100.00%
L57 A2226	100%	4 × 10^−103^	100.00%
L11107 A11394	100%	2 × 10^−105^	100.00%
L45961 A43028	100%	8 × 10^−134^	100.00%
L768 A11523	100%	4 × 10^−100^	98.55%
L1173 A8987	100%	3 × 10^−103^	99.25%
L1844 A410	100%	7 × 10^−97^	97.71%
L2633 A1002	100%	2 × 10^−94^	96.15%
L2633 A1003	100%	6 × 10^−98^	99.23%
L3948 A23172	100%	8 × 10^−102^	99.26%
L3982 A2731	100%	3 × 10^−101^	99.26%
L9011 A8085	100%	7 × 10^−105^	100.00%
L13738 A3768	100%	9 × 10^−101^	100.00%
L59460 A17957	100%	2 × 10^−108^	95.45%

**Table 2:** Indicator VOCs for the two sampling time points (before and after feeding by BSF larvae), for all the six diet treatments combined. MVOC, microbial volatile organic compounds, Y, yes; N, no. Data are reported as indicator value, showing the relative frequency and relative abundance of each compound that can be considered as a measure of exclusiveness for a species in a group.

Time Point	Compound	Indicator Value	*p* Value	MVOC
Before BSF Feeding	n-propyl acetate	0.9679	0.001	Y
	2-methyl-butanal	0.9304	0.001	Y
	acetic acid, butyl ester	0.9230	0.001	Y
	butanoic acid, butyl ester	0.8945	0.001	Y
	acetic acid, hexyl ester	0.8691	0.001	Y
	1-hexanol	0.8518	0.001	Y
	butyl 2-methylbutanoate	0.8496	0.001	Y
	acetic acid, pentyl ester	0.8465	0.001	Y
	beta pinene	0.8417	0.001	Y
	2-pentanol, acetate	0.8253	0.001	N
	butanoic acid, propyl ester	0.8122	0.001	Y
	2-hexenal	0.8071	0.001	Y
	acetic acid, 2-methylpropyl ester	0.7692	0.001	Y
	2-hexen-1-ol, (E)	0.7871	0.001	Y
	propanoic acid, ethyl ester	0.7423	0.001	Y
	1-butanol, 3-methyl-, acetate	0.6411	0.001	Y
	2-hexen-1-ol, acetate, (E)	0.6018	0.001	N
	3-methyl-butanal	0.5989	0.009	Y
	alpha pinene	0.5852	0.049	Y
	1-butanol, 2-methyl-, acetate	0.5109	0.001	Y
	acetic acid, 1-methylethyl ester	0.5000	0.001	Y
	propanoic acid, butyl ester	0.4867	0.001	Y
	butanoic acid, 2-methyl-, ethyl ester	0.4681	0.001	Y
	hexanoic acid, ethyl ester	0.4435	0.001	Y
	hexanoic acid, hexyl ester	0.4226	0.001	N
	3-methyl-2-butanol	0.4126	0.001	Y
	butanoic acid, 2-methyl-, propyl ester	0.3902	0.001	N
	butanoic acid, 2-methyl, hexyl ester	0.3614	0.002	N
	butanoic acid, 3-methylbutyl ester	0.3284	0.046	Y
	hexanoic acid, butyl ester	0.3036	0.001	Y
	butanoic acid, 1-methylethyl ester	0.2917	0.001	N
	propanoic acid, 1-methylethyl ester	0.2500	0.002	N
	propanoic acid, propyl ester	0.1455	0.030	Y
After BSF Feeding	styrene	0.9332	0.001	Y
	4-methyl octane	0.8898	0.001	N
	acetophenone	0.8898	0.001	Y
	2,4-dimethyl-1-heptene	0.8554	0.001	N
	1,4-dichloro-benzene	0.8179	0.001	N
	alpha farnesene	0.7572	0.001	Y
	4-methyl heptane	0.7538	0.001	Y
	3-hydroxy-2-butanone	0.7121	0.001	Y
	delta limonene	0.6809	0.001	Y
	3-octanone	0.6349	0.009	Y
	2-hexanone	0.3896	0.004	Y

**Table 3:** Indicator VOCs for each diet treatment. A, apple; B, banana; SG, spent grain; AB, apple and banana; ASG, apple and spent grain; BSG, banana and spent grain. MVOC, microbial volatile organic compounds; Y, yes; N, no.

Diet Treatment	Compound	Indicator Value	*p* Value	MVOC
A	propanoic acid, propyl ester	0.3411	0.001	Y
	hexanoic acid, butyl ester	0.3295	0.002	Y
	butanoic acid, 2-methyl-, propyl ester	0.3082	0.005	N
	hexanoic acid, hexyl ester	0.3039	0.003	N
	propanoic acid, butyl ester	0.2970	0.002	Y
	butanoic acid, 2-methyl, hexyl ester	0.2919	0.003	N
	alpha farnesene	0.2772	0.020	Y
	butanoic acid, propyl ester	0.2767	0.015	Y
	acetic acid, pentyl ester	0.2683	0.009	Y
	butanoic acid, 2-methyl-, ethyl ester	0.2521	0.017	Y
	butyl 2-methylbutanoate	0.2521	0.024	Y
	1-butanol, 2-methyl-, acetate	0.2416	0.018	Y
	2-hexen-1-ol, acetate, (E)	0.2165	0.018	N
B	2-pentanone	0.2647	0.016	Y
	butanoic acid, 3-methyl-, 3-methylbutyl ester	0.2385	0.035	Y
	3-methyl-2-butanol	0.1969	0.030	Y
SG	acetic acid, 1-methylethyl ester	0.2071	0.022	Y
	acetic acid, 1-methylpropyl ester	0.2019	0.011	Y
	propanoic acid, 1-methylethyl ester	0.1630	0.040	Y
AB	2-heptanone	0.2574	0.032	Y

**Table 4:** Differential production of 25 VOCs of interest, in response to larval feeding. A, apple; B, banana; SG, spent grain; AB, apple and banana; ASG, apple and spent grain; BSG, banana and spent grain.

Diet Treatment	Compound	*t*-Statistic	*p* Value	Response to BSF Feeding
A	acetic acid, 1-methylethyl ester	2.0330	0.0307	Decreased
	2-methyl-butanal	7.6570	<0.0001	Decreased
	propanoic acid, ethyl ester	6.1018	<0.0001	Decreased
	n-propyl acetate	12.5324	<0.0001	Decreased
	3-methyl 1 butanol	−2.6771	0.0090	Increased
	2-hexanone	−2.1064	0.0268	Increased
	acetic acid, butyl ester	9.0735	<0.0001	Decreased
	butanoic acid, 1-methylethyl ester	3.2234	0.0031	Decreased
	2-hexen-1-ol, (E)	13.3337	<0.0001	Decreased
	1-hexanol	17.6178	<0.0001	Decreased
	2-heptanone	−3.4129	0.0021	Increased
	styrene	−3.9625	0.0007	Increased
	butanoic acid, propyl ester	8.0026	<0.0001	Decreased
	3-octanone	−2.6621	0.0093	Increased
	hexanoic acid, ethyl ester	3.6954	0.0012	Decreased
	acetophenone	−2.9457	0.0053	Increased
	butanoic acid, 2-methyl, hexyl ester	6.8270	<0.0001	Decreased
B	2-methyl-butanal	5.2414	<0.0001	Decreased
	n-propyl acetate	4.1729	0.0005	Decreased
	acetic acid, 2-methylpropyl ester	2.8090	0.007	Decreased
	2-hexanone	−2.7025	0.0086	Increased
	acetic acid, butyl ester	4.6626	0.0002	Decreased
	2-hexen-1-ol, (E)	4.1726	0.0005	Decreased
	1-hexanol	4.4551	0.0003	Decreased
	1-butanol, 3-methyl-, acetate	2.8739	0.0061	Decreased
	styrene	−3.0277	0.0045	Increased
	3-octanone	−3.0591	0.0042	Increased
	delta limonene	−1.8663	0.0415	Increased
	acetophenone	−2.9461	0.0053	Increased
	alpha farnesene	−2.9470	0.0053	Increased
SG	acetic acid, 1-methylethyl ester	3.1821	0.0033	Decreased
	2-methyl-butanal	1.7853	0.0479	Decreased
	2-pentanone	−2.6189	0.0101	Increased
	propanoic acid, ethyl ester	23.4326	<0.0001	Decreased
	n-propyl acetate	5.6582	<0.0001	Decreased
	propanoic acid, 1-methylethyl ester	2.6301	0.0099	Decreased
	acetic acid, 2-methylpropyl ester	3.9253	0.0008	Decreased
	acetic acid, butyl ester	3.7345	0.0011	Decreased
	butanoic acid, 1-methylethyl ester	2.5664	0.0112	Decreased
	1-hexanol	3.1753	0.0034	Decreased
	1-butanol, 3-methyl-, acetate	3.1919	0.0033	Decreased
	styrene	−2.6347	0.0098	Increased
	butanoic acid, propyl ester	4.1825	0.0005	Decreased
	delta limonene	−2.2986	0.0187	Increased
	acetophenone	−2.7377	0.0080	Increased
	alpha farnesene	−2.7992	0.0071	Increased
AB	2-methyl-butanal	4.2259	0.0004	Decreased
	n-propyl acetate	5.4622	<0.0001	Decreased
	acetic acid, 2-methylpropyl ester	3.3697	0.0023	Decreased
	acetic acid, butyl ester	5.5960	<0.0001	Decreased
	2-hexen-1-ol, (E)	5.3248	<0.0001	Decreased
	1-hexanol	3.3854	0.0022	Decreased
	1-butanol, 3-methyl-, acetate	3.2337	0.0030	Decreased
	styrene	−2.7290	0.0082	Increased
	butanoic acid, propyl ester	4.1006	0.0005	Decreased
	3-octanone	−1.8185	0.0452	Increased
	acetophenone	−2.1429	0.0251	Increased
ASG	acetic acid, 1-methylethyl ester	3.4404	0.0020	Decreased
	2-methyl-butanal	18.781	<0.0001	Decreased
	propanoic acid, ethyl ester	24.1909	<0.0001	Decreased
	n-propyl acetate	9.9488	<0.0001	Decreased
	3-methyl 1 butanol	3.1033	0.0039	Decreased
	propanoic acid, 1-methylethyl ester	2.5636	0.0113	Decreased
	acetic acid, 2-methylpropyl ester	7.5709	<0.0001	Decreased
	acetic acid, butyl ester	18.0855	<0.0001	Decreased
	butanoic acid, 1-methylethyl ester	2.3669	0.0164	Decreased
	2-hexen-1-ol, (E)	3.9159	0.0008	Decreased
	1-hexanol	6.0055	<0.0001	Decreased
	1-butanol, 3-methyl-, acetate	12.6400	<0.0001	Decreased
	styrene	−2.6991	0.0086	Increased
	butanoic acid, propyl ester	4.0969	0.0005	Decreased
	hexanoic acid, ethyl ester	7.4051	<0.0001	Decreased
	delta limonene	−1.8678	0.0414	Increased
	butanoic acid, 2-methyl, hexyl ester	4.0931	0.0005	Decreased
	alpha farnesene	−1.8727	0.0411	Increased
BSG	3-methyl butanal	1.9782	0.0347	Decreased
	acetic acid, 1-methylethyl ester	3.1037	0.0042	Decreased
	1-butanol	1.8738	0.0418	Decreased
	2-methyl-butanal	5.3343	<0.0001	Decreased
	2-pentanone	3.0636	0.0045	Decreased
	propanoic acid, ethyl ester	3.0325	0.0048	Decreased
	n-propyl acetate	6.3571	<0.0001	Decreased
	3-methyl 1 butanol	2.4682	0.0141	Decreased
	propanoic acid, 1-methylethyl ester	2.3981	0.0161	Decreased
	acetic acid, 2-methylpropyl ester	5.5777	<0.0001	Decreased
	acetic acid, butyl ester	14.2486	<0.0001	Decreased
	2-hexen-1-ol, (E)	5.2596	<0.0001	Decreased
	1-hexanol	9.2635	<0.0001	Decreased
	1-butanol, 3-methyl-, acetate	6.0414	<0.0001	Decreased
	styrene	−2.4923	0.0135	Increased
	butanoic acid, propyl ester	2.5515	0.0121	Decreased
	acetophenone	−1.7904	0.0483	Increased
	alpha farnesene	−2.4558	0.0144	Increased

**Table 5:** Identification and analysis of the overall pockets on OBP surfaces with CASTp server. The amino acid residues in the main binding pocket, which were most directly involved in ligand interactions, were highlighted in yellow. Properties of each specific contact are the nearest distance (Å), the contact surface area (Å^2^) and the contact volume (Å^3^) between atoms of the putative ligand and the protein residues. The presence of aromatic and non-polar amino acid residues led to a high percentage of hydrophobicity for HillOBP_C57 and HillOBP_C21691 binding pockets.

*Hill*OBPs 3D Structure	Highlighted Residues Involved in Ligand Binding	Number of Pockets	Mouths	Area of Solvent Access Surface (Å^2^)	Area of Molecular Surface (Å^2^)	Volume of Solvent Access Surface (Å^3^)	Volume of Molecular Surface (Å^3^)	Diameter Pocket (Å)	Hydro-Phobicity (%)
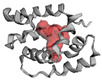	**>*Hill*OBP_C57** tytikthddliktrglcvkelnvpdnyvekfkkwdfqddettrcyikcvlnkmelfdtangfnvenlveqlgqnkdktevrtevtkcsdkneqksddctwayrgfkcflskhlqlvqssvks	16	1	275.84	615.78	116.07	729.82	285.99	79.31
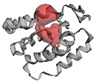	**>*Hill*OBP_C11107** ewvprtsdqmykdqaecfkqlelteeeqqkvkkedfpdepkfrcylrcilmggqiwddekgynperayaellnidmtadvenlrkcntqnlhhsdsctrafrvvkcfannnyitsikpks	21	4	338.23	753.68	185.62	901.17	316.48	56.67
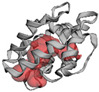	**>*Hill*OBP_C21691** nvndpklksileqcigsekaspadiaalearssdlskeakcviscvmknykllsddgkvnrdvfmaeaeemtkgdagamkeagemfeicsaktvadpcesafnfghcmktemtarnipmdf	17	0	228.54	579.12	83.35	628.65	258.62	72.41
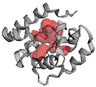	**>*Hill*OBP_C1173** nwstptkeqfkqhrddclkegnvpeetankirkeqypndrdtycyircvgsksgiwndrkgydidrslqvfeangyevtrenlercfaplpgadtctwagvnmrclrdnkyvtkkasa	19	1	313.85	807.34	136.89	900.72	378.30	53.33
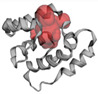	**>*Hill*OBP_C2633** isteefqemreecfksekvpeadieklkhreygldlgheakcyirclgmktgnwddtngydvekiytdfrtaglevtkenlkkcfkssgdddkcvwaakdlkclwtnkymsrkq	15	2	382.45	654.31	285.81	1005.38	334.98	53.85
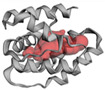	**>*Hill*OBP_C13368** dvndprlkaslekcigsekaspadvealkahssdlsreaqcvmacvmkefkllgddgkinrdvymaeaeemakgdagaikqatemydicsaktvadncesannfgqciknemiarnipldm	15	1	379.24	841.48	199.76	1011.53	376.49	69.70
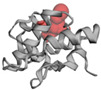	**>*Hill*OBP_C13738** dwkprsreqytkdgdecfksenisedgiheirrhvftddskcffrcvlmknhvwddttgynvervykevthiglkaskdgltqcnsddkkdkdpcqwvnnivrcvfehnyiepny	20	2	240.94	454.29	83.13	534.66	227.29	64.71
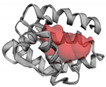	**>*Hill*OBP_C31956** kvdenklkaytaniaktcqpegepfgevhdivekanptqdekcfitctmtkwgllsengkfqpdgvrkvneairefddnpaeyknadeaiiakcsaiekpekcdkgyaiaecgfkvfdeihg	17	1	493.53	802.17	398.72	1286.26	360.19	62.16

**Table 6:** Virtual screening based on molecular docking. SwissDock server (https://www.swissdock.ch/docking/ accessed in June 2021) was used to simulate all the possible interactions between 57 selected VOCs indicative of organic decomposition and 8 putative OBPs during the computational analysis. Free binding energy values (ΔG, kcal/mol) are listed, showing the best cluster of each specific interaction. All the identified OBPs, in common between *H. illucens* larvae and adults, seem to interact almost in the same way in presence of a specific ligand.

Energy (ΔG, kcal/mol)								
VOCs	OBP_C*57*	OBP_C*1173*	OBP_C*2633*	OBP_C*13368*	OBP_C*31956*	OBP_C*13738*	OBP_C*21691*	OBP_C*11107*
hexanoic acid, hexyl ester	−47.94	−44.71	−47.88	−50.04	−42.63	−44.10	−44.80	−46.54
hexanoic acid, butyl ester	−43.91	−38.89	−41.47	−43.30	−38.31	−36.80	−46.54	−40.07
isopentyl hexanoate	−41.45	−38.45	−42.33	−43.53	−37.59	−38.02	−40.16	−39.96
butanoic acid, 2-methyl, hexyl ester	−41.05	−37.01	−41.14	−44.11	−38.98	−34.96	−39.68	−39.93
butanoic acid, butyl ester	−37.05	−32.86	−34.51	−36.28	−33.00	−32.86	−36.49	−34.70
acetic acid, hexyl ester	−36.18	−32.31	−35.05	−33.57	−30.26	−29.41	−33.80	−32.42
4-methyl octane	−35.89	−32.32	−34.26	−36.00	−30.94	−28.77	−32.71	−32.81
alpha-farnesene	−35.28	−24.42	−28.61	−32.20	−24.74	−23.14	−23.83	−28.01
butyl 2-methylbutanoate	−35.25	−32.64	−33.22	−37.15	−32.51	−32.64	−36.70	−34.74
hexanoic acid, ethyl ester	−35.04	−33.63	−33.06	−36.18	−32.34	−32.72	−37.84	−33.35
butanoic acid, 3-methylbutyl ester	−34.38	−33.09	−34.26	−36.28	−31.53	−31.87	−34.61	−32.56
butanoic acid, 1-methylbutyl ester	−34.38	−32.49	−35.52	−35.03	−30.97	−32.83	−34.21	−33.22
butanoic acid, 3-methyl-3-methylbutyl ester	−33.76	−30.52	−34.29	−36.00	−29.83	−32.11	−33.62	−32.76
3-octanone	−33.71	−31.42	−35.07	−33.79	−30.43	−30.12	−33.11	−31.79
2-hexen-1-ol, acetate, (E)	−33.38	−29.82	−33.25	−33.62	−28.98	−30.13	−28.31	−31.81
propanoic acid, butyl ester	−32.72	−29.33	−31.89	−31.91	−28.57	−30.43	−31.95	−29.91
2-pentyl furan	−32.43	−29.68	−30.36	−32.04	−29.62	−28.22	−32.03	−30.30
butanoic acid, 2-methylpropyl ester	−31.90	−29.04	−31.43	−33.34	−29.51	−29.81	−33.93	−30.38
butanoic acid, propyl ester	−31.55	−29.93	−31.18	−33.48	−30.91	−29.69	−32.69	−29.58
4-methyl heptane	−30.76	−29.85	−30.33	−32.79	−29.23	−25.92	−28.15	−29.10
acetic acid, pentyl ester	−30.72	−27.43	−30.94	−31.29	−28.75	−28.36	−31.82	−30.26
2-heptanone	−29.46	−26.79	−29.01	−29.35	−25.71	−26.06	−29.62	−27.76
1-hexanol	−29.32	−25.85	−25.81	−29.88	−26.30	−26.15	−30.47	−26.77
propanoic acid, propyl ester	−28.88	−25.80	−28.11	−28.36	−26.91	−26.89	−29.73	−26.57
butanoic acid, 1-methylethyl ester	−28.83	−27.45	−27.90	−30.74	−27.90	−26.73	−28.66	−26.76
butanoic acid, 2-methylethyl ester	−28.65	−25.84	−27.18	−28.34	−25.48	−25.62	−27.01	−26.02
2-pentanol, acetate	−28.00	−24.31	−26.44	−26.66	−24.66	−24.22	−29.30	−26.61
delta-limonene	−27.91	−26.76	−28.21	−29.21	−26.70	−22.69	−21.09	−27.35
2,4-dimethyl-1-heptene	−27.39	−26.02	−25.72	−29.29	−24.97	−22.14	−28.62	−26.47
1-butanol, 2-methylacetate	−27.09	−25.47	−25.78	−26.93	−24.25	−24.79	−25.50	−25.00
acetic acid, butyl ester	−26.80	−25.56	−26.00	−28.94	−25.32	−26.61	−29.31	−25.27
2-hexen-1-ol, (E)	−26.20	−24.79	−25.66	−26.60	−25.26	−25.42	−32.58	−26.75
1-butanol, 3-methylacetate	−26.14	−24.49	−25.64	−27.37	−25.89	−23.93	−27.22	−24.16
2-hexanone	−25.85	−23.64	−25.61	−26.60	−23.43	−22.82	−26.35	−23.95
propanoic acid, 1-methylethyl ester	−25.53	−23.77	−25.29	−25.81	−22.65	−24.15	−25.12	−24.08
n-propyl acetate	−24.80	−21.68	−22.74	−26.14	−21.99	−22.52	−25.69	−22.64
propanoic acid, ethyl ester	−24.69	−22.47	−24.29	−25.05	−22.39	−24.17	−26.50	−23.16
2-hexenal	−23.33	−22.47	−22.57	−23.08	−21.96	−21.73	−26.85	−22.35
acetic acid, 1-methylpropyl ester	−23.29	−22.29	−23.13	−24.17	−21.49	−20.94	−24.45	−22.29
acetic acid, 2-methylpropyl ester	−22.76	−22.32	−23.70	−25.17	−21.75	−22.03	−23.71	−22.07
2-pentanone	−21.72	−19.76	−22.39	−21.59	−19.42	−19.93	−23.73	−20.49
acetic acid, 1-methylethyl ester	−21.70	−18.68	−19.94	−20.53	−18.17	−18.67	−23.24	−19.03
3-methyl butanal	−20.75	−19.37	−21.15	−20.96	−18.97	−18.32	−22.25	−19.28
1-butanol	−20.60	−20.04	−20.22	−21.18	−18.71	−20.06	−22.19	−19.72
2-methyl-1-butanol	−20.50	−18.55	−20.78	−21.18	−18.14	−17.66	−21.53	−18.66
3-methyl-1-butanol	−20.32	−19.39	−21.77	−20.13	−18.91	−17.74	−20.15	−18.92
2-methyl butanal	−19.51	−19.58	−20.86	−20.94	−17.96	−18.02	−22.68	−19.45
3-methyl-2-butanol	−16.91	−15.98	−19.07	−17.48	−15.41	−14.69	−17.10	−15.69
3-hydroxy-2-butanone	−14.95	−14.92	−16.23	−16.18	−13.73	−14.84	−16.17	−14.14
1,4-dichlorobenzene	−7.00	−4.91	−6.12	−6.56	−5.80	−5.84	−8.19	−5.46
benzaldehyde	−2.42	−2.43	−3.83	−2.84	−1.82	−2.91	−5.34	−2.08
acetophenone	−2.38	−1.47	−3.29	−2.60	−0.95	−2.57	−4.99	−2.91
styrene	−1.18	0.42	−0.49	0.69	0.40	−0.55	−3.34	−0.33
beta-pinene	46.13	41.79	46.36	41.49	43.11	49.55	50.76	43.80
alpha-pinene	51.27	45.95	50.38	46.01	48.30	52.29	54.58	48.70

## Data Availability

Data is contained within this article and the supplementary material.

## Supplementary Materials

The following are available online at https://www.mdpi.com/article/10.3390/insects12090814/s1. Table S1: Database of VOCs emitted from different food matrices. Table S2a: Blastx analysis of *Hermetia illucens* OBP genes from adult transcriptome. Table S2b: Blastx analysis of *Hermetia illucens* olfactory receptor genes from adult transcriptome. Table S2c: Blastx analysis of *Hermetia illucens* gustatory receptor genes from adult transcriptome. Table S2d: Blastx analysis of *Hermetia illucens* glutamate receptor genes from adult transcriptome. Table S2e: Blastx analysis of *Hermetia illucens* chemosensory protein genes from adult transcriptome. Table S2f: Blastx analysis of *Hermetia illucens* sensory neuron membrane genes from adult transcriptome. Figure S1: Phylogenetic trees of adult odorant binding proteins (a), olfactory receptors (b), ionotropic receptors (b’), gustatory receptors (c), glutamate receptors (d), chemosensory proteins (e), sensory neuron membrane proteins (f). Table S3a: Blastx analysis of *Hermetia illucens* odorant binding protein genes from larval transcriptome. Table S3b: Blastx analysis of *Hermetia illucens* ionotropic receptor genes from larval transcriptome. Table S3c: Blastx analysis of *Hermetia illucens* gustatory receptor genes from larval transcriptome. Table S3d: Blastx analysis of *Hermetia illucens* glutamate receptor genes from larval transcriptome. Table S3e: Blastx analysis of *Hermetia illucens* chemosensory protein genes from larval transcriptome. Table S3f: Blastx analysis of *Hermetia illucens* sensory neuron membrane genes from larval transcriptome. Figure S2: Phylogenetic trees of larval odorant binding proteins (a), ionotropic receptors (b), gustatory receptors (c), glutamate receptors (d), chemosensory proteins (e), sensory neuron membrane proteins (f). Table S4: analysis of the OBP sequences from adult transcriptome, searching for complete sequence at 5’ and 3’ ends, the presence of the signal peptide with SignalP-5.0 software and the conserved cysteine pattern. Table S5: Analysis of the OBP sequences from larval transcriptome, searching for complete sequence at 5’ and 3’ ends, the presence of the signal peptide with SignalP-5.0 software and the conserved cysteine pattern. Table S6: Complete OBP sequences from larvae and adult transcriptome were compared using the Basic Local Alignment Search Tool Protein (BLASTp). Figure S3: Sequence alignments performed by Clustal Omega, between the sequences of common OBPs in larval and adult transcriptomes. Table S7: Selected VOCs of interest, index of specific phases of organic degradation, in different food matrices.
