# Supplementary material for: Hermetia illucens (L.) (Diptera: Stratiomyidae) Odorant Binding Proteins and Their Interactions with Selected Volatile Organic Compounds: An In Silico Approach
Source: Insects. 2021 Sep 11;12(9):814. doi: 10.3390/insects12090814 (PMC8469849; doi:10.3390/insects12090814)
Supplement: Supplementary file 1 [file insects-12-00814-s001.zip › insects-sup-1366842.pdf]

***Hermetia illucens* (L.) (Diptera: Stratiomyidae) Odorant Binding Proteins and Their Interactions with Selected Volatile Organic Compounds: An *in Silico* Approach**

Carmen Scieuzo[1,2#], Marisa Nardiello[1#], Donatella Farina[1,2#], Andrea Scala[1], Jonathan A. Cammack[3], Jeffery K. Tomberlin[3], Heiko Vogel[4], Rosanna Salvia[1,2*], Krishna Persaud[5]*, Patrizia Falabella[1,2]*

1 Department of Sciences, University of Basilicata, Via dell'Ateneo Lucano 10, 85100, Potenza, Italy.
2 Spinoff XFlies s.r.l, University of Basilicata, Via dell'Ateneo Lucano 10, 85100, Potenza, Italy.
3 Department of Entomology, Texas A&M University, College Station, TX, USA
4 Department of Entomology, Max Planck Institute for Chemical Ecology, Hans-Knöll-Straße 8, D-07745, Jena, Germany.
5 Department of Chemical Engineering and Analytical Science, The University of Manchester, Manchester, M13 9PL, United Kingdom.
#equally

*corresponding authors: patrizia.falabella@unibas.it; krishna.persaud@manchester.ac.uk; r.salvia@unibas.it

VOC	REFERENCE
3-methyl-butanal	[105]
Acetic acid, 1-methylethyl ester	[106]
1-butanol	[107,108]
2-methyl-butanal	[109]
2-pentanone	[105,110,111]
3-methyl-2-butanol	[112]
3-hydroxy-2-butanone	[111,113]
propanoic acid, ethyl ester	[114]
n-propyl acetate	[115]
3-methyl 1 butanol	[108,116,117]
2-methyl 1 butanol	[108,117]
propanoic acid, 1-methylethyl ester	[118]
Acetic acid, 1-methylpropyl ester	[119]
4-methyl heptane	[105]
acetic acid, 2-methylpropyl ester	[120,121]

2-hexanone	[122,123]
propanoic acid, propyl ester	[124]
acetic acid, butyl ester	[115,122]
2,4-dimethyl-1-heptene	[125]
butanoic acid, 1-methylethyl ester	[126,127]
2-pentanol, acetate	[128]
butanoic acid, 2-methyl-, ethyl ester	[129]
2-hexenal	[130]
4-methyl octane	[131,132]
2-hexen-1-ol	[105,110]
1-hexanol	[111,130,133]
1-butanol, 3-methyl-, acetate	[134,135]
1-butanol, 2-methyl-, acetate	[136,137]
2-heptanone	[138-140]
Styrene	[141,142]
butanoic acid, propyl ester	[143]
propanoic acid, butyl ester	[144,145]
acetic acid, pentyl ester	[146,147]
alpha pinene	[110]
butanoic acid, 2-methyl-, propyl ester	[148]
Benzaldehyde	[108,111,149]
beta pinene	[150]
3-octanone	[151]
2-pentyl furan	[149,152-154]
butanoic acid, butyl ester	[153,155]
hexanoic acid, ethyl ester	[121]
acetic acid, hexyl ester	[156,157]
2-hexen-1-ol, acetate	[158]
1,4-dichloro-benzene	[159]
butanoic acid, 1-methylbutyl ester	[160]
Delta limonene	[161]
butyl 2-methylbutanoate	[158,162]
butanoic acid, 3-methylbutyl ester	[135]

Acetophenone	[163,164]
butanoic acid, 3-methyl-, 3-methylbutyl ester	[135]
hexanoic acid, butyl ester	[162,165]
butanoic acid, 2-methyl, hexyl ester	[166-168]
isopentyl hexanoate	[135,169]
hexanoic acid, hexyl ester	[147, 153,155]
Alpha farnesene	[166,170]
Phenol	[171]
Butanoic acid	[122]
Trimethylamine	[172]
4 ethyl phenol	[173]
1- propanol	[174,175]
Acetone	[176]
Isobutyraldehyde	[177]
Ethyl acetate	[178]
Ethanol	[179]
Geraniol	[176]
Linalool	[180]
Nonanal	[181]
Methyl propionate	[182]
Hexanal	[183,184]
Ethyl butanoate	[185]
(Z)-3-hexenal	[186]
Decanal	[186]
6-methyl-5-hepten-2-one	[187]
methyl ethyl ketone	[188]
toluene	[188]
Methyl 2-hydroxyhexanoate	[178]
Methyl 5-acetoxyhexanoate	[178]
3-Methylbutanoic acid	[176]
1-Heptanol	[176]
Geranial	[176]
Acetaldehyde	[189]

2-Pentyl-furan	[189]
2-nonanone	[190]
2-octenone	[190]
α-Caryophyllene	[191]
Pentanal	[184]
1-Hexene	[192]
2-methylpropanal	[193]
Methyl butanoate	[194]
2-Methyl butyl acetate	[195]
3-Methyl-4-ethylhexane	[196]
Allyl isothiocyanate	[197]
cis-3-hexenyl acetate	[198]
2-hexanol	[199]
2-Methylbutyl propanoate	[199]
Methyl anthranilate	[200]
1-nonanol	[187]
Phenylethyl alcohol	[201]
Dimethyl propanedioate	[178]
Ethyl 2-methylbutanoate	[202]

Table S1. **Database of VOCs emitted from different food matrices**. VOC name and reference are listed.

ODORANT BINDING PROTEINS				
**CONTIG NAME**	**BLASTX**	**QUERY COVER**	**E – VALUE**	**IDENTITY**
NHill_AD_C41290	pheromone-binding protein-related protein 6-like [Hermetia illucens]	98%	5e-57	78.07%
NHill_AD_C42622	general odorant-binding protein 84a isoform X1 [Hermetia illucens]	98%	8e-47	83.91%
NHill_AD_C41232	general odorant-binding protein 19d-like [Hermetia illucens]	59%	4e-94	98.58%
NHill_AD_C30834	general odorant-binding protein 19d-like [Hermetia illucens]	61%	1e-95	100%

NHill_AD_C54152	general odorant-binding protein 84a isoform X1 [Hermetia illucens]	98%	9e-41	84.15%
NHill_AD_C42252	general odorant-binding protein 84a isoform X2 [Hermetia illucens]	99%	7e-48	84.62%
NHill_AD_C18886	general odorant-binding protein 83a-like [Hermetia illucens]	8%	7e-91	99.31%
NHill_AD_C23028	general odorant-binding protein 99a-like [Hermetia illucens]	80%	6e-24	93.88%
NHill_AD_C2668	uncharacterized protein LOC119654785 [Hermetia illucens]<br>general odorant-binding protein 19d-like isoform X1 [Hermetia illucens]	76%<br>72%	9e-107<br>3e-64	100%<br>64.19%
NHill_AD_C2226	unnamed protein product [Hermetia illucens]<br>general odorant-binding protein 99a-like [Hermetia illucens]	42%<br>28%	1e-79	99.17%<br>100%
NHill_AD_C5246	uncharacterized protein LOC119655221 [Hermetia illucens]<br>general odorant-binding protein 99a-like [Hermetia illucens]	70%<br>63%	4e-108<br>6e-22	99.35%<br>36.23%
NHill_AD_C16472	OBP8 [Trichogramma japonicum]	86%	0.0099	30.51%
NHill_AD_C11937	general odorant-binding protein 99a-like isoform X2 [Hermetia illucens]	44%	7e-33	61.86%
NHill_AD_C11394	general odorant-binding protein 99a-like [Hermetia illucens]	50%	3e-80	97.79%
NHill_AD_C1500	general odorant-binding protein 28a-like [Hermetia illucens]	42%	5e-101	100.00%
NHill_AD_C17009	odorant-binding protein 59a [Hermetia illucens]	59%	8e-167	99.13%
NHill_AD_C74693	general odorant-binding protein 56d-like [Copidosoma floridanum]	63%	8e8-66	75.00%
NHill_AD_C4062	uncharacterized protein LOC119655996 [Hermetia illucens]<br>general odorant-binding protein 83a [Drosophila willistoni]	37%<br>14%	4e-74<br>3e-05	82.46%<br>40.00%
NHill_AD_C17957	general odorant-binding protein 28a-like [Hermetia illucens]	75%	4e-97	94.81%
NHill_AD_C75784	uncharacterized protein LOC119659326 [Hermetia illucens]<br>general odorant-binding protein 66-like [Hermetia illucens]	75%<br>72%	1e-55<br>9e-31	98.82%<br>62.20%
NHill_AD_C61546	unnamed protein product [Hermetia illucens]<br>general odorant-binding protein 66-like [Hermetia illucens]	95%<br>95%	5e-52<br>1e-32	100%<br>65.98%
NHill_AD_C43028	uncharacterized protein LOC119661578 [Hermetia illucens]<br>general odorant-binding protein 68-like [Hermetia illucens]	48%	5e-120	100%
NHill_AD_C73118	uncharacterized protein LOC119661137 [Hermetia illucens]<br>general odorant-binding protein 68-like [Hermetia illucens]	59%<br>59%	5e-44<br>8e-30	98.65%<br>71.62%
NHill_AD_C56633	odorant-binding protein 19 [Bradysia odoriphaga]<br>uncharacterized protein LOC119648416 [Hermetia illucens]	96%<br>99%	4e-23<br>8e-79	40.83%<br>96.77%
NHill_AD_C12724	general odorant-binding protein 68-like [Hermetia illucens]	86%	3e-112	98.14%
NHill_AD_C32770	general odorant-binding protein 99a-like [Hermetia illucens]	55%	9e-74	88.81%
NHill_AD_C1003	general odorant-binding protein 99b-like [Hermetia illucens]	96%	2e-87	96.92%

NHill_AD_C56175	uncharacterized protein LOC119654413 [Hermetia illucens]<br>general odorant-binding protein 99a-like [Hermetia illucens]	98%<br>98%	2e-77<br>6e-65	96.61%<br>78.81%
NHill_AD_C23172	general odorant-binding protein 99b-like [Hermetia illucens]	75%	1e-93	99.27%
NHill_AD_C8085	general odorant-binding protein 99a-like [Hermetia illucens]	52%	4e-84	100.00%
NHill_AD_C1903	uncharacterized protein LOC119656184 [Hermetia illucens]<br>general odorant-binding protein 99a-like isoform X2 [Hermetia illucens]	35%<br>27%	3e-113<br>1e-40	98.79%<br>51.91%
NHill_AD_C32259	general odorant-binding protein 99b-like [Hermetia illucens]	78%	1e-80	98.52%
NHill_AD_C11523	general odorant-binding protein 99a-like [Hermetia illucens]	71%	3e-98	100%
NHill_AD_C3768	general odorant-binding protein 99a-like [Hermetia illucens]	83%	8e-71	92.86%
NHill_AD_C2731	general odorant-binding protein 99a-like isoform X1 [Hermetia illucens]	56%	2e-104	97.40%
NHill_AD_C1002	general odorant-binding protein 99b-like [Hermetia illucens]	78%	2e-90	100%
NHill_AD_C32362	general odorant-binding protein 99a-like [Hermetia illucens]	60%	7e-22	49.07%
NHill_AD_C12769	general odorant-binding protein 99a-like [Hermetia illucens]	54%	4e-75	98.18%
NHill_AD_C5932	uncharacterized protein LOC119655003 [Hermetia illucens]<br>general odorant-binding protein 99a-like [Hermetia illucens]	61%<br>59%	1e-91<br>3e-63	97.01%<br>71.54%
NHill_AD_C9042	general odorant-binding protein 99a-like [Hermetia illucens]	88%	1e-82	100%
NHill_AD_C70911	uncharacterized protein LOC119654807 [Hermetia illucens]<br>general odorant-binding protein 99b-like [Hermetia illucens]	99%	7e-78<br>3e-52	98.28%<br>71.55%
NHill_AD_C8987	general odorant-binding protein 99a-like [Hermetia illucens]	71%	7e-95	99.25%
NHill_AD_C7966	general odorant-binding protein 99a-like isoform X1 [Hermetia illucens]	52%	1e-78	100%
NHill_AD_C410	general odorant-binding protein 99a-like isoform X1 [Hermetia illucens]	62%	7e-90	100%
NHill_AD_C61431	general odorant-binding protein 99a-like [Hermetia illucens]	61%	8e-17	46.79%
NHill_AD_C17801	general odorant-binding protein 99a-like [Hermetia illucens]	61%	8e-17	46.79%
NHill_AD_C76148	general odorant-binding protein 56d [Copidosoma floridanum]	74%	9e9-33	71.43%

Table S2a. Blastx analysis of *Hermetia illucens* OBP genes from adult transcriptome. Each adult *H. illucens* contig sequence was manually checked with the BLASTX program from the National Center for Biotechnology Information (NCBI). For each sequence, the best matched protein was reported, with the respective Query Cover, the E-value and the Percentage of Identity.

ODORANT RECEPTORS				
**CONTIG NAME**	**BLASTX**	**QUERY COVER**	**E – VALUE**	**IDENTITY**
NHill_AD_C21581	putative odorant receptor 92a [Hermetia illucens]	57%	5e-52	97.83%
NHill_AD_C41456	uncharacterized protein LOC119647277 [Hermetia illucens]<br>odorant receptor 63a-like isoform X2 [Hermetia illucens]	61%<br>61%	3e-175<br>3e-127	99.65%<br>70.77%
NHill_AD_C32477	odorant receptor 63a-like isoform X1 [Hermetia illucens]	38%	5e-179	82.66%
NHill_AD_C40886	odorant receptor 22c-like [Hermetia illucens]	72%	0.0	100%
NHill_AD_C44491	putative odorant receptor 71a [Hermetia illucens]	82%	2e-99	78.89%
NHill_AD_C44088	odorant receptor 56a-like [Hermetia illucens]	83%	0.0	98.78%
NHill_AD_C41545	odorant receptor 56a-like isoform X1 [Hermetia illucens]	86%	6e-112	98.80%
NHill_AD_C12746	uncharacterized protein LOC119646983 [Hermetia illucens]<br>odorant receptor 59a-like [Hermetia illucens]	47%<br>66%	4e-164<br>2e-118	100%<br>65.92%
NHill_AD_C42139	odorant receptor 33a-like isoform X1 [Hermetia illucens]	99%	0.0	97.37%
NHill_AD_C41137	odorant receptor coreceptor [Hermetia illucens]	21%	6e-11	89.19%
NHill_AD_C40973	odorant receptor 94a-like isoform X1 [Hermetia illucens]	66%	0.0	99.48%
NHill_AD_C41586	odorant receptor 94b-like [Hermetia illucens]	91%	4e-128	99.45%
NHill_AD_C19031	odorant receptor 94a-like [Hermetia illucens]	87%	0.0	99.48%
NHill_AD_C63662	odorant receptor 85c-like [Hermetia illucens]	59%	2e-45	96.30%
NHill_AD_C25031	odorant receptor 2a-like [Hermetia illucens]	100%	4e-64	100%
NHill_AD_C41079	odorant receptor 22c-like [Hermetia illucens]	70%	0.0	99.49%
NHill_AD_C31170	uncharacterized protein LOC119646234 [Hermetia illucens]<br>odorant receptor 63a-like isoform X2 [Hermetia illucens]	88%<br>88%	3e-147<br>5e-60	100%<br>49.54%
NHill_AD_C40852	odorant receptor 33a-like [Hermetia illucens]	87%	3e-85	100%
NHill_AD_C41000	odorant receptor 2a-like [Hermetia illucens]	75%	0.0	98.93%
NHill_AD_C29699	odorant receptor 2a-like [Hermetia illucens]	80%	0.0	99.24%
NHill_AD_C53183	odorant receptor 33b-like [Hermetia illucens]	66%	7e-129	98.91%
NHill_AD_C42212	odorant receptor 59a-like [Hermetia illucens]	92%	2e-135	65.15%
NHill_AD_C24181	odorant receptor 33a-like [Hermetia illucens]	59%	5e-167	98.04%
NHill_AD_C53408	putative odorant receptor 92a isoform X1 [Hermetia illucens]	86%	5e-88	100.00%
NHill_AD_C43841	unnamed protein product [Hermetia illucens]<br>odorant receptor 2a-like [Hermetia illucens]	86%<br>85%	0.0<br>1e-47	99.69%<br>29.71%
NHill_AD_C42051	odorant receptor 33a-like [Hermetia illucens]	99%	1e-87	100%
NHill_AD_C43031	odorant receptor 49b-like [Hermetia illucens]	27%	2e-59	99.03%

NHill_AD_C42994	odorant receptor 59a-like [Hermetia illucens]	98%	5e-51	98.82%
NHill_AD_C43077	putative odorant receptor 71a [Hermetia illucens]	58%	8e-159	95.82%
NHill_AD_C42222	putative odorant receptor 71a [Hermetia illucens]	86%	0.0	98.88%
NHill_AD_C44928	odorant receptor 94b-like [Hermetia illucens]	99%	5e-95	98.87%
NHill_AD_C41233	odorant receptor 94a-like [Hermetia illucens]	70%	0.0	99.20%
NHill_AD_C43775	odorant receptor 19a-like [Hermetia illucens]	89%	0.0	98.93%
NHill_AD_C46337	unnamed protein product [Hermetia illucens]<br>odorant receptor 19a-like [Hermetia illucens]	76%<br>64%	0.0<br>2e-27	94.92%<br>27.78%
NHill_AD_C23297	odorant receptor coreceptor [Hermetia illucens]	64%	0.0	100%
NHill_AD_C41392	odorant receptor 85b-like [Hermetia illucens]	87%	0.0	99.25%
NHill_AD_C43182	putative odorant receptor 92a isoform X2 [Hermetia illucens]	99%	2e-57	100%
NHill_AD_C42500	odorant receptor 23a-like [Hermetia illucens]	87%	0.0	99.73%
NHill_AD_C40891	odorant receptor 2a-like [Hermetia illucens]	95%	0.0	96.61%
NHill_AD_C41817	odorant receptor 2a-like [Hermetia illucens]	65%	2e-73	97.25%
NHill_AD_C45790	odorant receptor 33a-like [Hermetia illucens]	43%	0.0	99.20%
NHill_AD_C39414	odorant receptor 33a-like [Hermetia illucens]	99%	0.0	94.14%
NHill_AD_C40888	odorant receptor 33b-like isoform X2 [Hermetia illucens]	99%	7e-157	97.50%
NHill_AD_C47973	odorant receptor 33b-like isoform X3 [Hermetia illucens]	88%	8e-47	67.94%
NHill_AD_C41400	odorant receptor 33a-like [Hermetia illucens]	84%	2e-57	100%
NHill_AD_C34422	odorant receptor 33b-like [Hermetia illucens]	53%	3e-17	97.87%
NHill_AD_C41519	unnamed protein product [Hermetia illucens]<br>odorant receptor 33b-like [Hermetia illucens]	88%<br>88%	5e-76<br>6e-73	99.24%<br>99.24%
NHill_AD_C44475	odorant receptor 33b-like [Hermetia illucens]	97%	0.0	99.32%
NHill_AD_C42002	odorant receptor 59a-like [Hermetia illucens]	79%	7e-127	100%
NHill_AD_C45287	unnamed protein product [Hermetia illucens]<br>odorant receptor 33b-like [Hermetia illucens]	99%<br>99%	6e-128<br>2e-108	100.00%<br>87.91%
NHill_AD_C33589	odorant receptor 33a-like isoform X4 [Hermetia illucens]	97%	8e-158	95.58%
NHill_AD_C44439	odorant receptor 33a-like [Hermetia illucens]	51%	1e-53	98.80%
NHill_AD_C32178	odorant receptor 47a-like isoform X3 [Hermetia illucens]	99%	4e-99	86.29%
NHill_AD_C42199	odorant receptor 33a-like [Hermetia illucens]	95%	0.0	100.00%
NHill_AD_C41082	odorant receptor 33a-like isoform X1 [Hermetia illucens]	99%	5e-158	99.54%
NHill_AD_C22258	odorant receptor 33b-like [Hermetia illucens]	34%	0.0	99.73%
NHill_AD_C41468	odorant receptor 2a-like [Hermetia illucens]	30%	4e-65	77.54%
NHill_AD_C68947	odorant receptor 63a-like [Hermetia illucens]	68%	6e-58	100%
NHill_AD_C11445	odorant receptor 63a-like [Hermetia illucens]	73%	0.0	99.73%

NHill_AD_C41945	odorant receptor 2a-like [Hermetia illucens]	99%	9e-73	98.21%
NHill_AD_C44667	odorant receptor 59a-like [Hermetia illucens	82%	3e-154	64.22%
NHill_AD_C35353	odorant receptor 33a-like isoform X10 [Hermetia illucens]	31%	3e-17	93.75%
NHill_AD_C8206	odorant receptor 33a-like [Hermetia illucens]	73%	0.0	74.35%
NHill_AD_C41435	odorant receptor 59a-like [Hermetia illucens]	95%	0.0	100%
NHill_AD_C54061	putative odorant receptor 85e [Hermetia illucens]	57%	2e-48	100%
NHill_AD_C41180	uncharacterized protein LOC119658097 [Hermetia illucens]<br>odorant receptor 94a-like [Hermetia illucens]	67%<br>65%	0.0<br>1e-89	98.99%<br>41.30%
NHill_AD_C42546	odorant receptor 33a-like [Hermetia illucens]	97%	0.0	100%
NHill_AD_C41347	odorant receptor 33a-like isoform X2 [Hermetia illucens]	85%	0.0	92.28%
NHill_AD_C45661	odorant receptor 33a-like [Hermetia illucens]	86%	0.0	94.72%
NHill_AD_C41258	odorant receptor 33a-like isoform X2 [Hermetia illucens]	99%	0.0	98.39%
NHill_AD_C7984	odorant receptor 13a [Hermetia illucens]	68%	0.0	98.78%
NHill_AD_C38560	uncharacterized protein LOC119646234 [Hermetia illucens]<br>odorant receptor 63a-like isoform X2 [Hermetia illucens]	55%<br>54%	0.0<br>4e-113	89.74%<br>50.26%
NHill_AD_C54695	putative odorant receptor 92a [Hermetia illucens]	91%	1e-117	92.74%
NHill_AD_C43878	odorant receptor 56a-like isoform X2 [Hermetia illucens]	43%	1e-24	100%
NHill_AD_C42007	odorant receptor 33a-like [Hermetia illucens]	70%	8e-117	100%
NHill_AD_C43319	odorant receptor 33a-like isoform X2 [Hermetia illucens]	99%	2e-42	100%
NHill_AD_C41351	odorant receptor 59a-like [Hermetia illucens]	59%	2e-175	98.12%
NHill_AD_C43290	odorant receptor 33b-like [Hermetia illucens]	71%	0.0	98.01%
NHill_AD_C45322	odorant receptor 2a-like isoform X1 [Hermetia illucens]	77%	0.0	100%
NHill_AD_C25667	odorant receptor 2a-like [Hermetia illucens]	73%	0.0	98.70%
NHill_AD_C26571	odorant receptor 33a-like [Hermetia illucens]	97%	0.0	98.37%
NHill_AD_C30148	unnamed protein product [Hermetia illucens]<br>odorant receptor 33a-like [Hermetia illucens]	83%<br>79%	0.0	99.75%<br>99.74%
NHill_AD_C42377	odorant receptor 2a-like [Hermetia illucens]	82%	0.0	74.93%
NHill_AD_C41922	odorant receptor 2a-like [Hermetia illucens]	77%	1e-173	68.25%
NHill_AD_C44474	odorant receptor 33b-like [Hermetia illucens]	99%	2e-152	100%
NHill_AD_C42132	odorant receptor 33b-like [Hermetia illucens]	95%	0.0	98.16%
NHill_AD_C43759	odorant receptor 59b-like [Hermetia illucens]	79%	0.0	100%
NHill_AD_C41186	odorant receptor 2a-like isoform X1 [Hermetia illucens]	84%	0.0	99.73%
NHill_AD_C41447	odorant receptor 2a-like [Hermetia illucens]	90%	3e-128	88.39%
NHill_AD_C41520	odorant receptor 33a-like [Hermetia illucens]	89%	1e-62	83.72%

NHill_AD_C42523	odorant receptor 33a-like isoform X1 [Hermetia illucens]	88%	0.0	100%
NHill_AD_C40853	odorant receptor 33a-like isoform X1 [Hermetia illucens]	74%	1e-151	88.46%
NHill_AD_C35696	odorant receptor 33a-like isoform X3 [Hermetia illucens]	52%	0.0	87.91%
NHill_AD_C41761	odorant receptor 67c-like isoform X1 [Hermetia illucens]	80%	0.0	95.65%
NHill_AD_C45972	putative odorant receptor 92a [Hermetia illucens]	89%	0.0	99.73%
NHill_AD_C15333	putative odorant receptor 92a isoform X2 [Hermetia illucens]	81%	0.0	97.97%
NHill_AD_C42441	putative odorant receptor 92a [Hermetia illucens]	68%	0.0	98.71%
NHill_AD_C21580	putative odorant receptor 92a [Hermetia illucens]	82%	0.0	81.40%
NHill_AD_C41243	putative odorant receptor 92a [Hermetia illucens]	93%	0.0	93.29%
NHill_AD_C41341	ionotropic receptor IR2 [Colaphellus bowringi]<br>uncharacterized protein LOC119651354 isoform X1 [Hermetia illucens]	75%<br>90%	9e-101<br>0.0	36.20%<br>98.96%
NHill_AD_C11008	ionotropic receptor 75a-like isoform X1 [Hermetia illucens]	47%	4e-121	100%
NHill_AD_C21099	ionotropic receptor 75a-like isoform X3 [Hermetia illucens]	40%	6e-128	100%
NHill_AD_C42032	ionotropic receptor 21a isoform X1 [Hermetia illucens]	83%	0.0	99.62%
NHill_AD_C20652	ionotropic receptor 25a [Hermetia illucens]	72%	1e-121	100%
NHill_AD_C43496	ionotropic receptor [Bactrocera dorsalis]<br>LOW QUALITY PROTEIN: uncharacterized protein LOC119655012 [Hermetia illucens]	80%<br>99%	2e-09<br>2e-64	38.30%<br>93.69%
NHill_AD_C44323	ionotropic receptor 56b [Drosophila melanogaster]<br>uncharacterized protein LOC119655200 [Hermetia illucens]	80.5%<br>100%	97%<br>0.0	1e-12<br>97.35%
NHill_AD_C38475	ionotropic receptor 56c [Drosophila melanogaster]<br>unnamed protein product [Hermetia illucens]	41%<br>61%	1e-04<br>2e-50	24.35%<br>48.52%
NHill_AD_C66692	ionotropic receptor 56d [Drosophila melanogaster]<br>unnamed protein product [Hermetia illucens]	92%<br>99%	2e-10<br>3e-105	28.38%<br>96.20%
NHill_AD_C52359	unnamed protein product [Hermetia illucens]<br>glutamate receptor ionotropic, delta-2 [Lucilia sericata]	50%<br>61%	2e-130<br>6e-07	95.38%<br>25.88%
NHill_AD_C41221	putative ionotropic receptor IR8 [Scaeva pyrastri]<br>uncharacterized protein LOC119650383 [Hermetia illucens]	69%<br>79%	4e-94<br>0.0	55.78%<br>98.81%
NHill_AD_C44460	ionotropic receptor [Procecidochares utilis]<br>uncharacterized protein LOC119650383 [Hermetia illucens]	50%<br>99%	1e-84<br>0.0	61.00%<br>98.74%
NHill_AD_C41222	putative ionotropic receptor IR8 [Scaeva pyrastri]<br>uncharacterized protein LOC119650383 [Hermetia illucens]	49%<br>38%	1e-26<br>6e-28	77.14%<br>98.18%
NHill_AD_C53362	ionotropic receptor 67c [Drosophila melanogaster]<br>unnamed protein product [Hermetia illucens]	71%<br>93%	2e-09<br>0.0	23.29%<br>96.17%

NHill_AD_C72456	ionotropic receptor 67c [Drosophila melanogaster]<br>unnamed protein product [Hermetia illucens]	98%<br>100%	3e-15<br>5e-94	30.91%<br>94.90%
NHill_AD_C44637	ionotropic receptor 75a-like [Hermetia illucens]	94%	0.0	93.33%
NHill_AD_C30659	ionotropic receptor 93a isoform X2 [Hermetia illucens]	87%	0.0	99.72%
NHill_AD_C43588	unnamed protein product [Hermetia illucens]<br>glutamate receptor ionotropic, delta-1 [Hermetia illucens]	100%<br>66%	7e-130<br>1e-84	96.37%<br>97.66%
NHill_AD_C6138	unnamed protein product [Hermetia illucens]<br>ionotropic receptor 25a [Hermetia illucens]	65%<br>66%	0.0<br>0.0	98.04%<br>98.16%
NHill_AD_C47154	putative ionotropic receptor IR11 [Scaeva pyrastri]<br>uncharacterized protein LOC119651974 isoform X1 [Hermetia illucens]	64%<br>90%	1e-49<br>3e-139	44.86%<br>97.30%
NHill_AD_C31205	putative ionotropic receptor IR6 [Scaeva pyrastri]<br>uncharacterized protein LOC119651974 isoform X1 [Hermetia illucens]	50%<br>45%	2e-13<br>2e-24	58.93%<br>98.04%
NHill_AD_C62171	ionotropic receptor 85a [Peridroma saucia]<br>unnamed protein product [Hermetia illucens]	89%<br>99%	4e-04<br>6e-64	32.00%<br>90.99%
NHill_AD_C34789	ionotropic receptor 25a [Hermetia illucens]	92%	0.0	100%
NHill_AD_C22408	putative ionotropic receptor IR2 [Scaeva pyrastri]<br>uncharacterized protein LOC119651108 isoform X1 [Hermetia illucens]	78%<br>93%	2e-164<br>0.0	46.53%<br>99.06%
NHill_AD_C31183	75a-like isoform X3 [Hermetia illucens]	99%	2e-58	98.98
NHill_AD_C46271	ionotropic receptor 25a [Hermetia illucens]	16%	1e-05	95.45%
NHill_AD_C41702	ionotropic receptor 40a [Hermetia illucens]	65%	8e-65	95.58%

Table S2b. Blastx analysis of *Hermetia illucens* olfactory receptor genes from adult transcriptome. Each adult *H. illucens* contig sequence was manually checked with the BLASTX program from the National Center for Biotechnology Information (NCBI). For each sequence, the best matched protein was reported, with the respective Query Cover, the E-value and the Percentage of Identity.

**GUSTATORY RECEPTORS**				
**CONTIG NAME**	**BLASTX**	**QUERY COVER**	**E – VALUE**	**IDENTITY**
NHill_AD_C30527	gustatory and odorant receptor 22-like [Hermetia illucens]	82%	1e-155	78.33%
NHill_AD_C67675	putative gustatory receptor 28b isoform X2 [Hermetia illucens]	63%	7e-78	100%
NHill_AD_C30866	gustatory receptor for sugar taste 64f isoform X3 [Drosophila pseudoobscura]<br>unnamed protein product [Hermetia illucens]	99%<br>99%	7e-30<br>8e-66	47.17%<br>100%

NHill_AD_C19982	uncharacterized protein LOC119650006 [Hermetia illucens]<br>putative gustatory receptor 28a [Culex quinquefasciatus]	56%<br>35%	8e-94<br>5e-09	98.11%<br>33.98%
NHill_AD_C41457	gustatory and odorant receptor 22-like [Hermetia illucens]	99%	2e-91	100%
NHill_AD_C41699	gustatory and odorant receptor 22-like [Hermetia illucens]	30%	1e-163	100%
NHill_AD_C78658	gustatory and odorant receptor 22-like [Teleopsis dalmanni]	59%	8e-33	84.44%
NHill_AD_C29794	putative gustatory receptor 28b isoform X1 [Hermetia illucens]	91%	1e-98	99.40%
NHill_AD_C29688	putative gustatory receptor 28b isoform X2 [Hermetia illucens]	68%	7e-149	100%
NHill_AD_C51136	uncharacterized protein LOC119647209 [Hermetia illucens]<br>putative gustatory receptor 28b isoform X3 [Hermetia illucens]	81%<br>81%	1e-39<br>2e-37	38.22%<br>36.65%
NHill_AD_C29460	putative gustatory receptor 28b isoform X1 [Hermetia illucens]	81%	9e-168	98.74%
NHill_AD_C49312	putative gustatory receptor 2a [Hermetia illucens]	83%	00.0	97.29%
NHill_AD_C40950	gustatory receptor 68a-like isoform X4 [Hermetia illucens]	37%	2e-137	98.52%
NHill_AD_C66298	gustatory and pheromone receptor 33a [Hermetia illucens]	100%	3e-79	96.72%
NHill_AD_C51573	putative gustatory receptor 57a [Hermetia illucens]	66%	1e-162	98.68%
NHill_AD_C10478	putative gustatory receptor 59f [Hermetia illucens]	75%	5e-50	100%
NHill_AD_C40866	gustatory and odorant receptor 63a [Hermetia illucens]	86%	0.0	100%
NHill_AD_C44258	gustatory receptor for sugar taste 64a-like [Hermetia illucens]	85%	0.0	99.30%
NHill_AD_C55553	PREDICTED: gustatory receptor for sugar taste 64f-like [Drosophila rhopaloa]<br>unnamed protein product [Hermetia illucens]	59%<br>63%	4e-64<br>3e-95	71.01%<br>99.32%
NHill_AD_C58700	PREDICTED: gustatory receptor for sugar taste 64f-like [Drosophila rhopaloa]<br>unnamed protein product [Hermetia illucens]	25%<br>26%	9e-12<br>1e-18	79.55%<br>100%
NHill_AD_C41107	gustatory receptor for bitter taste 66a-like isoform X2 [Hermetia illucens]	86%	4e-117	98.82%
NHill_AD_C17291	gustatory receptor for bitter taste 66a [Hermetia illucens]	32%	4e4e-28	96.77%
NHill_AD_C8761	putative gustatory receptor 28b isoform X3 [Hermetia illucens]	100%	0.0	99.63%
NHill_AD_C39103	gustatory receptor [Eogystia hippophaecolus[	98%	4e-26	66.97%
NHill_AD_C27799	gustatory receptor 127 [Tribolium castaneum]<br>unnamed protein product [Hermetia illucens]	76%<br>58%	2e-04<br>5e-29	31.82%<br>79.10%

Table S2c. Blastx analysis of *Hermetia illucens* gustatory receptor genes from adult transcriptome. Each adult *H. illucens* contig sequence was manually checked with the BLASTX program from the National Center for Biotechnology Information (NCBI). For each sequence, the best matched protein was reported, with the respective Query Cover, the E-value and the Percentage of Identity.

GLUTAMATE RECEPTORS				
**CONTIG NAME**	**BLASTX**	**QUERY COVER**	**E – VALUE**	**IDENTITY**
NHill_AD_C29958	probable glutamate receptor [Hermetia illucens]	99%	0.0	99.25%
NHill_AD_C60142	glutamate receptor ionotropic NMDA receptor 3 [Bradysia odoriphaga]<br>uncharacterized protein LOC119661346 isoform X1 [Hermetia illucens]	94%<br>99%	7e-21<br>6e-50	64.90%<br>99.30%
NHill_AD_C53624	probable glutamate receptor [Hermetia illucens]	76%	2e-52	100%
NHill_AD_C47020	glutamate receptor ionotropic, kainate 2 isoform X2 [Hermetia illucens]	29%	4e-19	100%
NHill_AD_C32005	glutamate receptor ionotropic, kainate 2-like isoform X1 [Hermetia illucens]	99%	3e-40	98.94%
NHill_AD_C37253	glutamate receptor ionotropic, kainate 2 isoform X1 [Hermetia illucens]	72%	2e-160	100%
NHill_AD_C6128	glutamate receptor ionotropic, kainate 2 isoform X1 [Hermetia illucens]	71%	0.0	99.63%
NHill_AD_C6794	glutamate receptor ionotropic, kainate 1 isoform X3 [Hermetia illucens]	74%	0.0	100%
NHill_AD_C39066	glutamate receptor ionotropic, kainate 2 isoform X2 [Hermetia illucens]	40%	2e-128	100%
NHill_AD_C16314	glutamate receptor ionotropic, kainate 2 isoform X3 [Hermetia illucens]	96%	0.0	99.24%
NHill_AD_C19616	glutamate receptor ionotropic, kainate 2 isoform X1 [Hermetia illucens]	99%	3e-136	99.49%
NHill_AD_C17391	glutamate receptor ionotropic, kainate 2-like [Hermetia illucens]	91%	0.0	99.55%
NHill_AD_C26460	glutamate receptor ionotropic, kainate 2-like [Hermetia illucens]	93%	0.0	99.80%
NHill_AD_C19042	glutamate receptor ionotropic, kainate 2-like [Hermetia illucens]	95%	0.0	98.29%
NHill_AD_C1262	glutamate receptor ionotropic, kainate 2 isoform X1 [Hermetia illucens]	62%	0.0	99.69%
NHill_AD_C17336	glutamate receptor ionotropic, kainate 2-like isoform X2 [Hermetia illucens]	99%	6e-75	99.17%
NHill_AD_C22050	glutamate receptor ionotropic, kainate 2 [Hermetia illucens]	80%	6e-96	79.28%
NHill_AD_C78255	PREDICTED: glutamate receptor ionotropic, kainate 2-like [Rhagoletis zephyria]	97%	6e6-19	40.16%

NHill_AD_C7692	glutamate receptor ionotropic, kainate 2-like isoform X1 [Hermetia illucens]	93%	0.0	99.82%
NHill_AD_C67273	glutamate receptor ionotropic, delta-2 [Bactrocera oleae]<br>uncharacterized protein LOC101463425 isoform X1 [Ceratitis capitata]	79%<br>79%	5e-27<br>1e-29	28.57%<br>31.63%
NHill_AD_C21662	glutamate receptor ionotropic, kainate 2 [Hermetia illucens]	99%	5e-100	100%
NHill_AD_C52327	glutamate receptor ionotropic, delta-1 [Hermetia illucens]	99%	2e-50	100%
NHill_AD_C26161	glutamate receptor ionotropic, delta-1-like [Hermetia illucens]	89%	0.0	99.71%
NHill_AD_C40776	probable glutamate receptor [Hermetia illucens]	99%	1e-150	98.70%

Table S2d. Blastx analysis of *Hermetia illucens* glutamate receptor genes from adult transcriptome. Each adult *H. illucens* contig sequence was manually checked with the BLASTX program from the National Center for Biotechnology Information (NCBI). For each sequence, the best matched protein was reported, with the respective Query Cover, the E-value and the Percentage of Identity.

**CHEMOSENSORY PROTEINS**				
**CONTIG NAME**	**BLASTX**	**QUERY COVER**	**E – VALUE**	**IDENTITY**
NHill_AD_C35093	chemosensory protein [Dioryctria abietella]	57%	1e-65	89.91%
NHill_AD_C3992	ejaculatory bulb-specific protein 3-like [Hermetia illucens]	40%	6e-73	100%
NHill_AD_C12670	chemosensory protein A 7a [Drosophila melanogaster]<br>uncharacterized protein LOC119655481 [Hermetia illucens]	46%<br>71%	3e-12<br>7e-106	35.00%<br>97.75%
NHill_AD_C11820	chemosensory protein 5 [Bradysia odoriphaga]<br>unnamed protein product [Hermetia illucens]	39%<br>53%	4e-55<br>2e-164	59.22%<br>99.17%

Table S2e. Blastx analysis of *Hermetia illucens* chemosensory protein genes from adult transcriptome. Each adult *H. illucens* contig sequence was manually checked with the BLASTX program from the National Center for Biotechnology Information (NCBI). For each sequence, the best matched protein was reported, with the respective Query Cover, the E-value and the Percentage of Identity.

**SENSORY NEURON MEMBRANE PROTEINS**				
**CONTIG NAME**	**BLASTX**	**QUERY COVER**	**E – VALUE**	**IDENTITY**
NHill_AD_C14048	LOW QUALITY PROTEIN: sensory neuron membrane protein 2 [Hermetia illucens]	50%	0.0	100%
NHill_AD_C40885	sensory neuron membrane protein 1 [Hermetia illucens]	100%	5e-83	99.32%

Table S2f Blastx analysis of *Hermetia illucens* sensory neuron membrane genes from adult transcriptome. Each adult *H. illucens* contig sequence was manually checked with the BLASTX program from the National Center for Biotechnology Information (NCBI). For each sequence, the best matched protein was reported, with the respective Query Cover, the E-value and the Percentage of Identity.

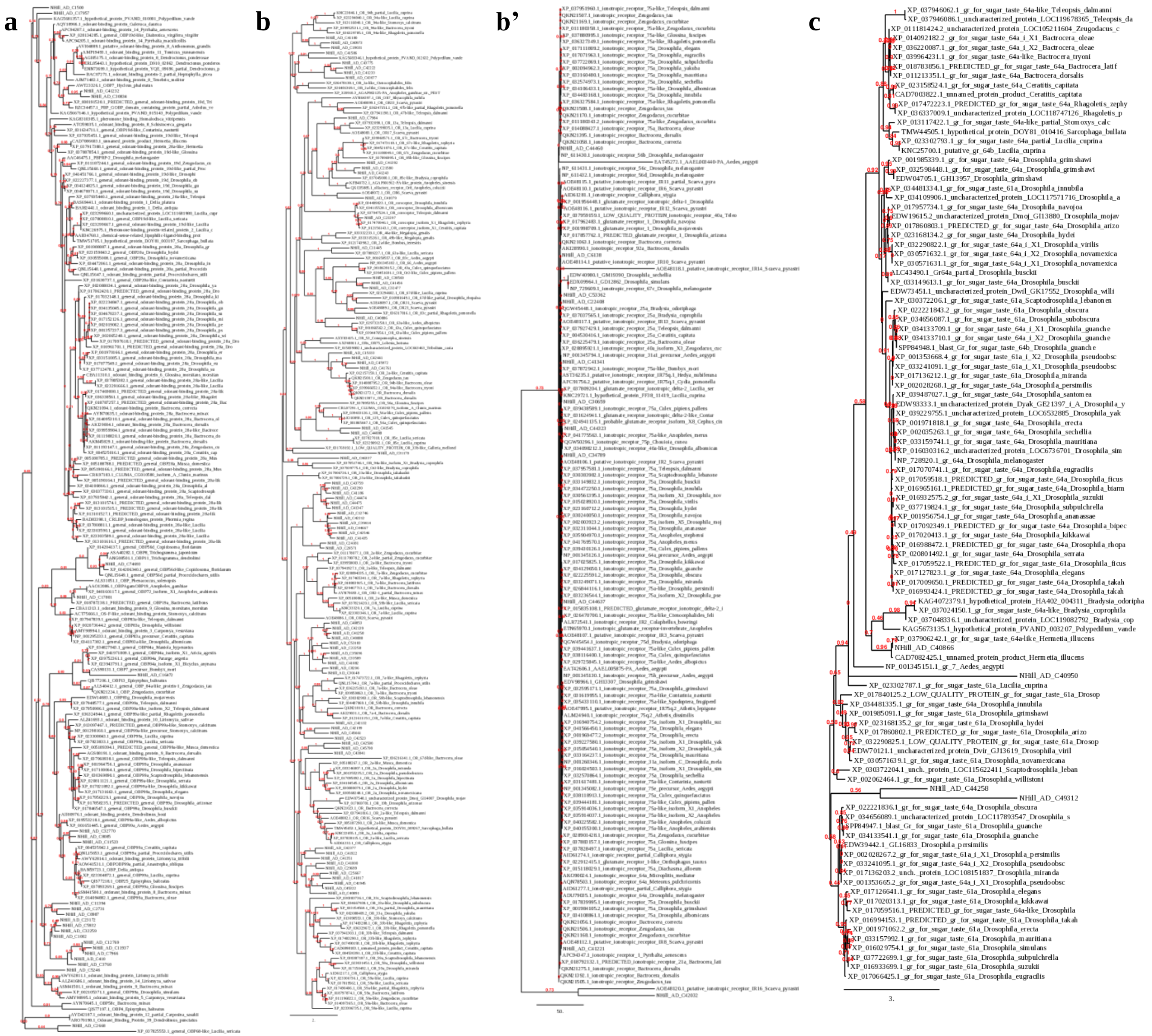
a
b
b'
c

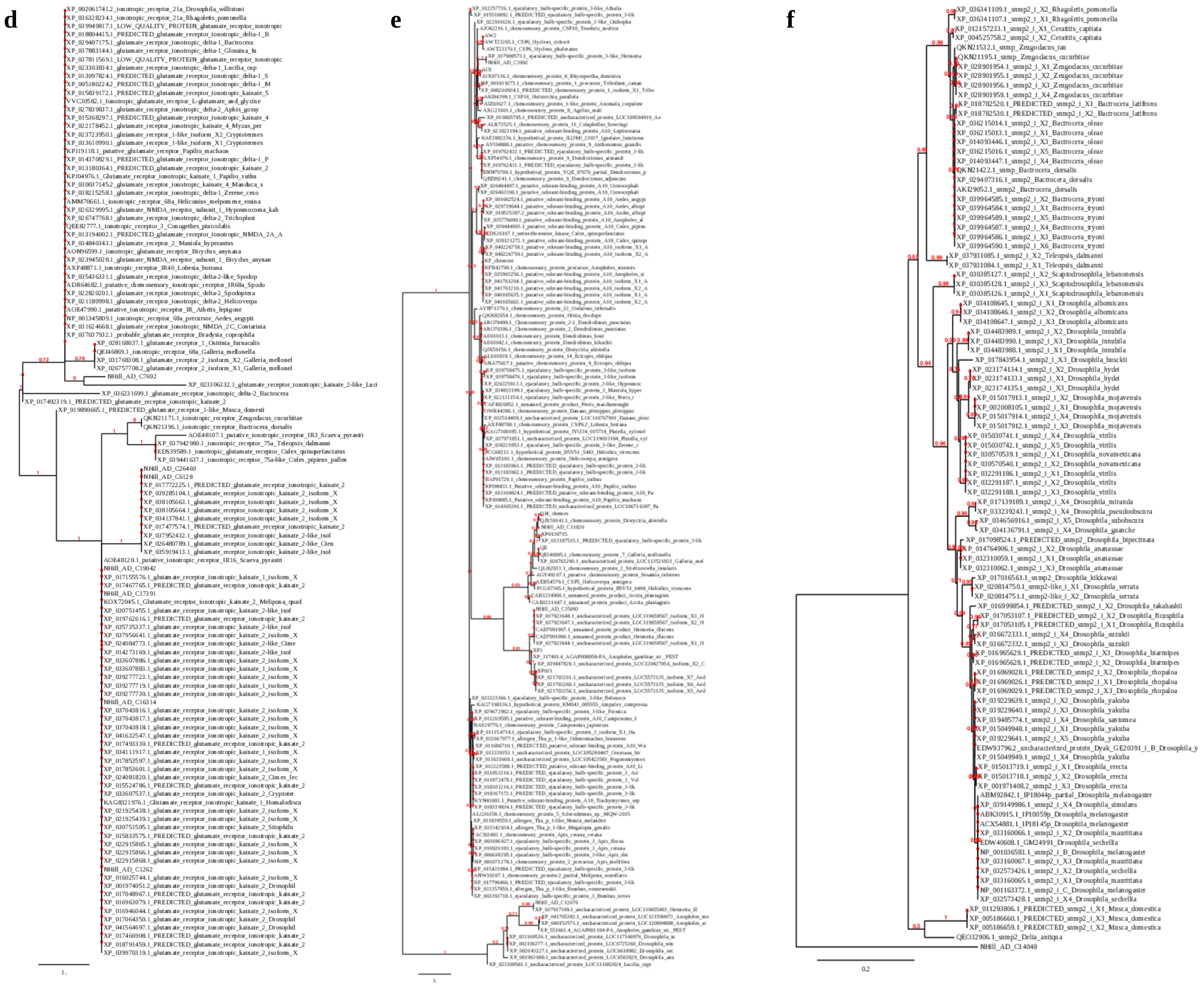
d
e
f
1.
1.
0.2

Figure S1: Phylogenetic trees of adult odorant binding proteins (a), olfactory receptors (b), ionotropic receptors (b'), gustatory receptors (c), glutamate receptors (d), chemosensory proteins (e), sensory neuron membrane proteins (f). Phylogenetic trees were built with Phylogeny Analysis software (http://www.phylogeny.fr/alacarte.cgi). Each sequence of *H. illucens* were manually checked with Blastx software. For each *H. illucens* sequence, the most similar sequences of different species or genus were selected. The corresponding amino acid sequences of all the selected sequences were subsequently aligned with MUSCLE software and then the phylogenetic tree was built, using default parameters. *H. illucens* sequences are reported as the contig number, while sequences of other species are reported with the accession number, the name of the protein. The Arabic numerals at the branching points are bootstrap values.

ODORANT BINDING PROTEINS				
**CONTIG NAME**	**BLASTX**	**QUERY COVER**	**E – VALUE**	**IDENTITY**
Hill_COMB-BB_C21691	general odorant-binding protein 19d-like [Hermetia illucens]	66%	2e-95	99.29%
Hill_COMB-BB_C13368	general odorant-binding protein 19d-like [Hermetia illucens]	71%	3e-96	100%
Hill_COMB-BB_C57	general odorant-binding protein 99a-like [Hermetia illucens]	33%	2e-80	100%
Hill_COMB-BB_C11107	general odorant-binding protein 99a-like [Hermetia illucens]	66%	3e-81	97.79%
Hill_COMB-BB_C31956	general odorant-binding protein 19d-like isoform X1 [Hermetia illucens]	74%	3e-89	100%
Hill_COMB-BB_C4812	odorant binding protein 15 [Holotrichia oblita]<br>uncharacterized protein LOC119648416 [Hermetia illucens]	99%<br>99%	2e-08<br>1e-50	40.48%<br>100%
Hill_COMB-BB_C7755	odorant-binding protein 50c [Bactrocera minax]<br>unnamed protein product [Hermetia illucens]	86%<br>97%	3e-38<br>4e-175	35.37%<br>97.30%
Hill_COMB-BB_C31807	general odorant-binding protein 66-like [Hermetia illucens]	93%	2e-112	98.74%
Hill_COMB-BB_C42512	unnamed protein product [Hermetia illucens]<br>general odorant-binding protein 66-like [Hermetia illucens]	96%	6e-69<br>9e-45	100%<br>65.83%
Hill_COMB-BB_C51370	uncharacterized protein LOC119646539 [Hermetia illucens]<br>general odorant-binding protein 66-like [Hermetia illucens]	80%<br>75%	6e-125<br>6e-31	96.76%<br>39.43%
Hill_COMB-BB_C45961	unnamed protein product [Hermetia illucens]<br>general odorant-binding protein 68-like [Hermetia illucens]	70%<br>68%	3e-74<br>6e-32	100%<br>48.57%
Hill_COMB-BB_C441	general odorant-binding protein 99a-like [Hermetia illucens]	57%	2e-90	100%
Hill_COMB-BB_C758	general odorant-binding protein 99b-like [Hermetia illucens]	65%	1e-83	95.38%
Hill_COMB-BB_C768	general odorant-binding protein 99a-like [Hermetia illucens]	80%	3e-109	98.84%
Hill_COMB-BB_C1173	general odorant-binding protein 99a-like [Hermetia illucens]	65%	5e-95	100%
Hill_COMB-BB_C1844	general odorant-binding protein 99a-like isoform X1 [Hermetia illucens]	80%	1e-90	100%
Hill_COMB-BB_C2633	general odorant-binding protein 99b-like [Hermetia illucens]	64%	4e-87	96.15%
Hill_COMB-BB_C3948	general odorant-binding protein 99b-like [Hermetia illucens]	85%	6e-72	99.07%

Hill_COMB-BB_C3962	general odorant-binding protein 99b-like [Hermetia illucens]	90%	5e-73	83.33%
Hill_COMB-BB_C3982	general odorant-binding protein 99a-like isoform X1 [Hermetia illucens]	90%	3e-98	100%
Hill_COMB-BB_C4168	general odorant-binding protein 99a-like isoform X3 [Hermetia illucens]	68%	4e-73	91.45%
Hill_COMB-BB_C5161	general odorant-binding protein 99a-like isoform X3 [Hermetia illucens]	85%	3e-72	90.60%
Hill_COMB-BB_C5826	general odorant-binding protein 99b-like [Hermetia illucens]	98%	4e-55	97.70%
Hill_COMB-BB_C7404	general odorant-binding protein 99b-like [Hermetia illucens]	96%	1e-77	81.02%
Hill_COMB-BB_C7621	general odorant-binding protein 99b-like [Hermetia illucens]	76%	2e-94	99.26%
Hill_COMB-BB_C9011	general odorant-binding protein 99a-like [Hermetia illucens]	80%	2e-91	100%
Hill_COMB-BB_C13738	general odorant-binding protein 99a-like [Hermetia illucens	58%	2e-72	93.04%
Hill_COMB-BB_C14606	uncharacterized protein LOC119655003 [Hermetia illucens]<br>general odorant-binding protein 99a-like [Hermetia illucens]	86%<br>83%	1e-84<br>6e-58	99.19%<br>72.27%
Hill_COMB-BB_C15984	general odorant-binding protein 99a-like isoform X2 [Hermetia illucens]	90%	9e-51	63.41%
Hill_COMB-BB_C29339	general odorant-binding protein 99b-like [Hermetia illucens]	87%	1e-62	92.31%
Hill_COMB-BB_C171	general odorant-binding protein 99a-like [Hermetia illucens]	65%	1e-73	97.62%
Hill_COMB-BB_C363	general odorant-binding protein 99a-like [Hermetia illucens]	87%	5e-93	100%
Hill_COMB-BB_C1936	general odorant-binding protein 99a-like isoform X2 [Hermetia illucens]	82%	9e-52	70.09%
Hill_COMB-BB_C13737	uncharacterized protein LOC119653464 [Hermetia illucens]<br>general odorant-binding protein 99a-like [Hermetia illucens]	69%<br>68%	5e-75<br>4e-49	100%<br>67.54%
Hill_COMB-BB_C3485	odorant-binding protein 50c [Bactrocera minax]<br>uncharacterized protein LOC119648416 [Hermetia illucens]	98%<br>100%	7e-10<br>3e-51	38.60%<br>97.39%
Hill_COMB-BB_C59460	general odorant-binding protein 28a-like [Hermetia illucens]	71%	3e-97	95.45%

Table S3a. Blastx analysis of *Hermetia illucens* odorant binding protein genes from larval transcriptome. Each larval *H. illucens* contig sequence was manually checked with the BLASTX program from the National Center for Biotechnology Information (NCBI). For each sequence, the best matched protein was reported, with the respective Query Cover, the E-value and the Percentage of Identity.

IONOTROPIC RECEPTORS				
Hill_COMB-BB_C13514	uncharacterized protein LOC119653703 [Hermetia illucens]<br>ionotropic receptor 25a [Hermetia illucens]	26%<br>10%	0.0<br>4e-49	100%<br>100.00%

Table S3b. Blastx analysis of *Hermetia illucens* ionotropic receptor genes from larval transcriptome. Each larval *H. illucens* contig sequence was manually checked with the BLASTX program from the National Center for Biotechnology Information (NCBI). For each sequence, the best matched protein was reported, with the respective Query Cover, the E-value and the Percentage of Identity.

**GUSTATORY RECEPTORS**				
**CONTIG NAME**	**BLASTX**	**QUERY COVER**	**E – VALUE**	**IDENTITY**
Hill_COMB-BB_C54783	gustatory receptor for bitter taste 66a [Hermetia illucens]	71%	2e-174	99.22%

Table S3c. Blastx analysis of *Hermetia illucens* gustatory receptor genes from larval transcriptome. Each larval *H. illucens* contig sequence was manually checked with the BLASTX program from the National Center for Biotechnology Information (NCBI). For each sequence, the best matched protein was reported, with the respective Query Cover, the E-value and the Percentage of Identity.

**GLUTAMMATE RECEPTORS**				
**CONTIG NAME**	**BLASTX**	**QUERY COVER**	**E – VALUE**	**IDENTITY**
Hill_COMB-BB_C17749	glutamate receptor ionotropic, kainate 2-like [Hermetia illucens]	92%	0.0	99.77%
Hill_COMB-BB_C37807	glutamate receptor ionotropic, kainate 2-like isoform X1 [Hermetia illucens]	98%	0.0	100%
Hill_COMB-BB_C24498	glutamate receptor ionotropic, kainate 1 isoform X3 [Hermetia illucens]	90%	0.0	100%
Hill_COMB-BB_C36919	glutamate receptor ionotropic, kainate 1 isoform X3 [Hermetia illucens]	95%	2e-142	100%
Hill_COMB-BB_C24819	glutamate receptor ionotropic, kainate 2-like isoform X1 [Hermetia illucens]	95%	0.0	99.83%

Table S3d. Blastx analysis of *Hermetia illucens* glutamate receptor genes from larval transcriptome. Each larval *H. illucens* contig sequence was manually checked with the BLASTX program from the National Center for Biotechnology Information (NCBI). For each sequence, the best matched protein was reported, with the respective Query Cover, the E-value and the Percentage of Identity.

**CHEMOSENSORY PROTEINS**				
**CONTIG NAME**	**BLASTX**	**QUERY COVER**	**E – VALUE**	**IDENTITY**
Hill_COMB-BB_C6211	chemosensory protein 5 [Bradysia odoriphaga]<br>unnamed protein product [Hermetia illucens]	46%<br>58%	2e-53<br>3e-135	61.25%<br>99.50%
Hill_COMB-BB_C11757	ejaculatory bulb-specific protein 3-like [Hermetia illucens]	33%	2e-55	100%

Table S3e. Blastx analysis of *Hermetia illucens* chemosensory protein genes from larval transcriptome. Each larval *H. illucens* contig sequence was manually checked with the BLASTX program from the National Center for Biotechnology Information (NCBI). For each sequence, the best matched protein was reported, with the respective Query Cover, the E-value and the Percentage of Identity.

**SENSORY NEURON MEMBRANE PROTEINS**				
**CONTIG NAME**	**BLASTX**	**QUERY COVER**	**E – VALUE**	**IDENTITY**
Hill_COMB-BB_C25571	LOW QUALITY PROTEIN: sensory neuron membrane protein 2 [Hermetia illucens]	60%	0.0	99.09%

Table S3f. Blastx analysis of *Hermetia illucens* sensory neuron membrane genes from larval transcriptome. Each larval *H. illucens* contig sequence was manually checked with the BLASTX program from the National Center for Biotechnology Information (NCBI). For each sequence, the best matched protein was reported, with the respective Query Cover, the E-value and the Percentage of Identity.

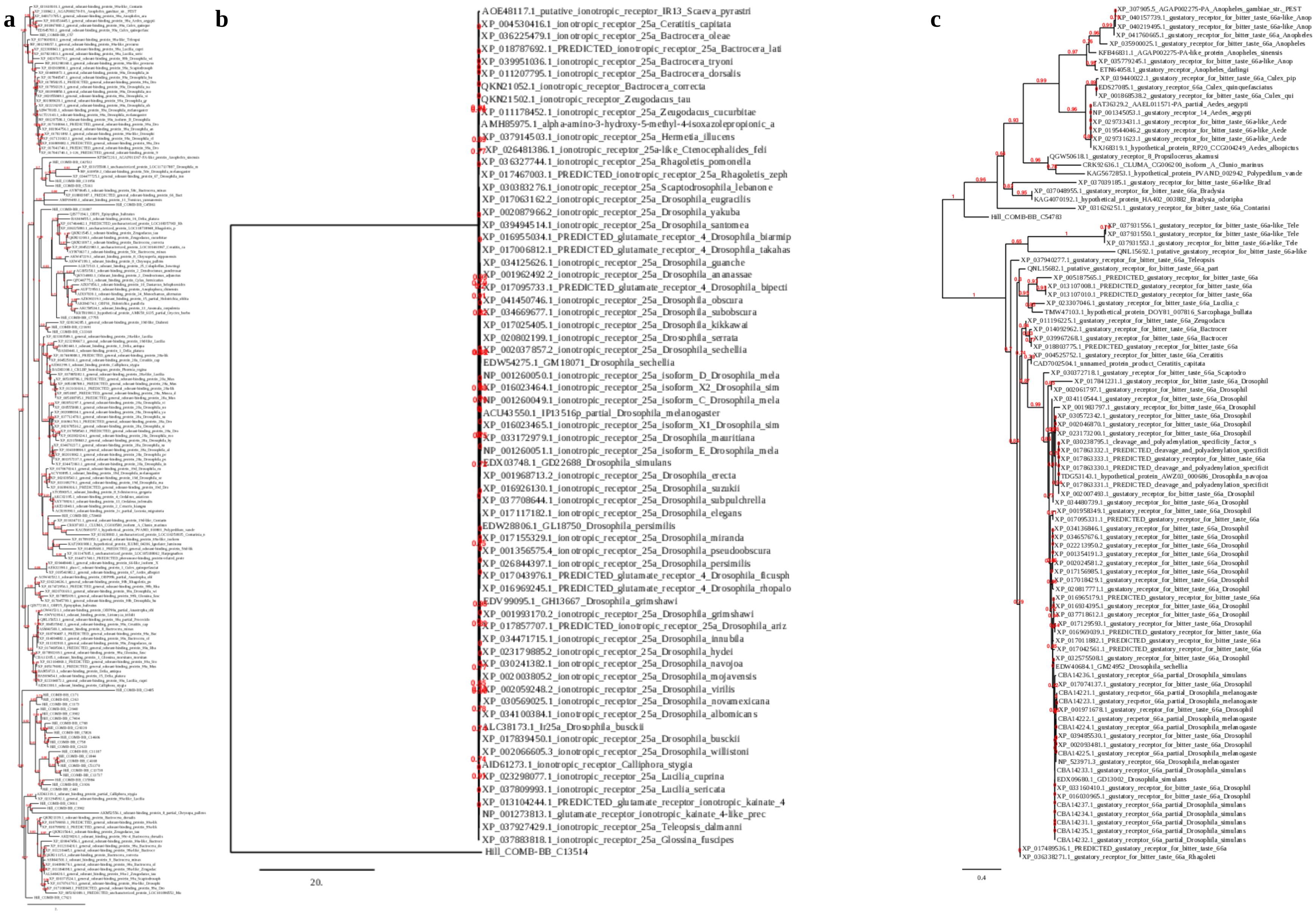
a
b
c
AOE48117.1_putative_ionotropic_receptor_IR13_Scaeva_pyrastri
XP_004530416.1_ionotropic_receptor_25a_Ceratitis_capitata
XP_036225479.1_ionotropic_receptor_25a_Bactrocera_oleae
XP_018787692.1_PREDICTED_ionotropic_receptor_25a_Bactrocera_lati
XP_039951036.1_ionotropic_receptor_25a_Bactrocera_tryoni
XP_011207795.1_ionotropic_receptor_25a_Bactrocera_dorsalis
QKN21052.1_ionotropic_receptor_Bactrocera_correcta
QKN21502.1_ionotropic_receptor_Zeugodacus_tau
XP_011178452.1_ionotropic_receptor_25a_Zeugodacus_cucurbitae
AMH85975.1_alpha-amino-3-hydroxy-5-methyl-4-isoxazolepropionic_a
XP_037914503.1_ionotropic_receptor_25a_Hermetia_illucens
XP_026481386.1_ionotropic_receptor_25a-like_Ctenocephalides_feli
XP_036327744.1_ionotropic_receptor_25a_Rhagoletis_pomonella
XP_017467003.1_PREDICTED_ionotropic_receptor_25a_Rhagoletis_zeph
XP_030383276.1_ionotropic_receptor_25a_Scaptodrosophila_lebanone
XP_017063162.2_ionotropic_receptor_25a_Drosophila_eugracilis
XP_002087966.2_ionotropic_receptor_25a_Drosophila_yakuba
XP_039494514.1_ionotropic_receptor_25a_Drosophila_santomea
XP_016955034.1_PREDICTED_glutamate_receptor_4_Drosophila_biarmip
XP_017006812.1_PREDICTED_glutamate_receptor_4_Drosophila_takahas
XP_034125626.1_ionotropic_receptor_25a_Drosophila_guanche
XP_001962492.2_ionotropic_receptor_25a_Drosophila_ananassae
XP_017095733.1_PREDICTED_glutamate_receptor_4_Drosophila_bipecti
XP_041450746.1_ionotropic_receptor_25a_Drosophila_obscura
XP_034669677.1_ionotropic_receptor_25a_Drosophila_subobscura
XP_017025405.1_ionotropic_receptor_25a_Drosophila_kikkawai
XP_020802199.1_ionotropic_receptor_25a_Drosophila_serrata
XP_002037857.2_ionotropic_receptor_25a_Drosophila_sechellia
EDW54275.1_GM18071_Drosophila_sechellia
NP_001260050.1_ionotropic_receptor_25a_isoform_D_Drosophila_mela
XP_016023464.1_ionotropic_receptor_25a_isoform_X2_Drosophila_sim
NP_001260049.1_ionotropic_receptor_25a_isoform_C_Drosophila_mela
ACU43550.1_IP13516p_partial_Drosophila_melanogaster
XP_016023465.1_ionotropic_receptor_25a_isoform_X1_Drosophila_sim
XP_033172979.1_ionotropic_receptor_25a_Drosophila_mauritiana
NP_001260051.1_ionotropic_receptor_25a_isoform_E_Drosophila_mela
EDX03748.1_GD22688_Drosophila_simulans
XP_001968713.2_ionotropic_receptor_25a_Drosophila_erecta
XP_016926130.1_ionotropic_receptor_25a_Drosophila_suzukii
XP_037708644.1_ionotropic_receptor_25a_Drosophila_subpulchrella
XP_017117182.1_ionotropic_receptor_25a_Drosophila_elegans
EDW28806.1_GL18750_Drosophila_persimilis
XP_017155329.1_ionotropic_receptor_25a_Drosophila_miranda
XP_001356575.4_ionotropic_receptor_25a_Drosophila_pseudoobscura
XP_026844397.1_ionotropic_receptor_25a_Drosophila_persimilis
XP_017043976.1_PREDICTED_glutamate_receptor_4_Drosophila_ficusph
XP_016969245.1_PREDICTED_glutamate_receptor_4_Drosophila_rhopalo
EDV99095.1_GH13667_Drosophila_grimshawi
XP_001993170.2_ionotropic_receptor_25a_Drosophila_grimshawi
XP_017857707.1_PREDICTED_ionotropic_receptor_25a_Drosophila_ariz
XP_034471715.1_ionotropic_receptor_25a_Drosophila_innubila
XP_023179885.2_ionotropic_receptor_25a_Drosophila_hydei
XP_030241382.1_ionotropic_receptor_25a_Drosophila_navojoa
XP_002003805.2_ionotropic_receptor_25a_Drosophila_mojavensis
XP_002059248.2_ionotropic_receptor_25a_Drosophila_virilis
XP_030569025.1_ionotropic_receptor_25a_Drosophila_novamexicana
XP_034100384.1_ionotropic_receptor_25a_Drosophila_albomicans
ALC38173.1_Ir25a_Drosophila_busckii
XP_017839450.1_ionotropic_receptor_25a_Drosophila_busckii
XP_002066605.3_ionotropic_receptor_25a_Drosophila_willistoni
AID61273.1_ionotropic_receptor_Calliphora_stygia
XP_023298077.1_ionotropic_receptor_25a_Lucilia_cuprina
XP_037809993.1_ionotropic_receptor_25a_Lucilia_sericata
XP_013104244.1_PREDICTED_glutamate_receptor_ionotropic_kainate_4
NP_001273813.1_glutamate_receptor_ionotropic_kainate_4-like_prec
XP_037927429.1_ionotropic_receptor_25a_Teleopsis_dalmanni
XP_037883818.1_ionotropic_receptor_25a_Glossina_fuscipes
Hill_COMB-BB_C13514
20.
0.4

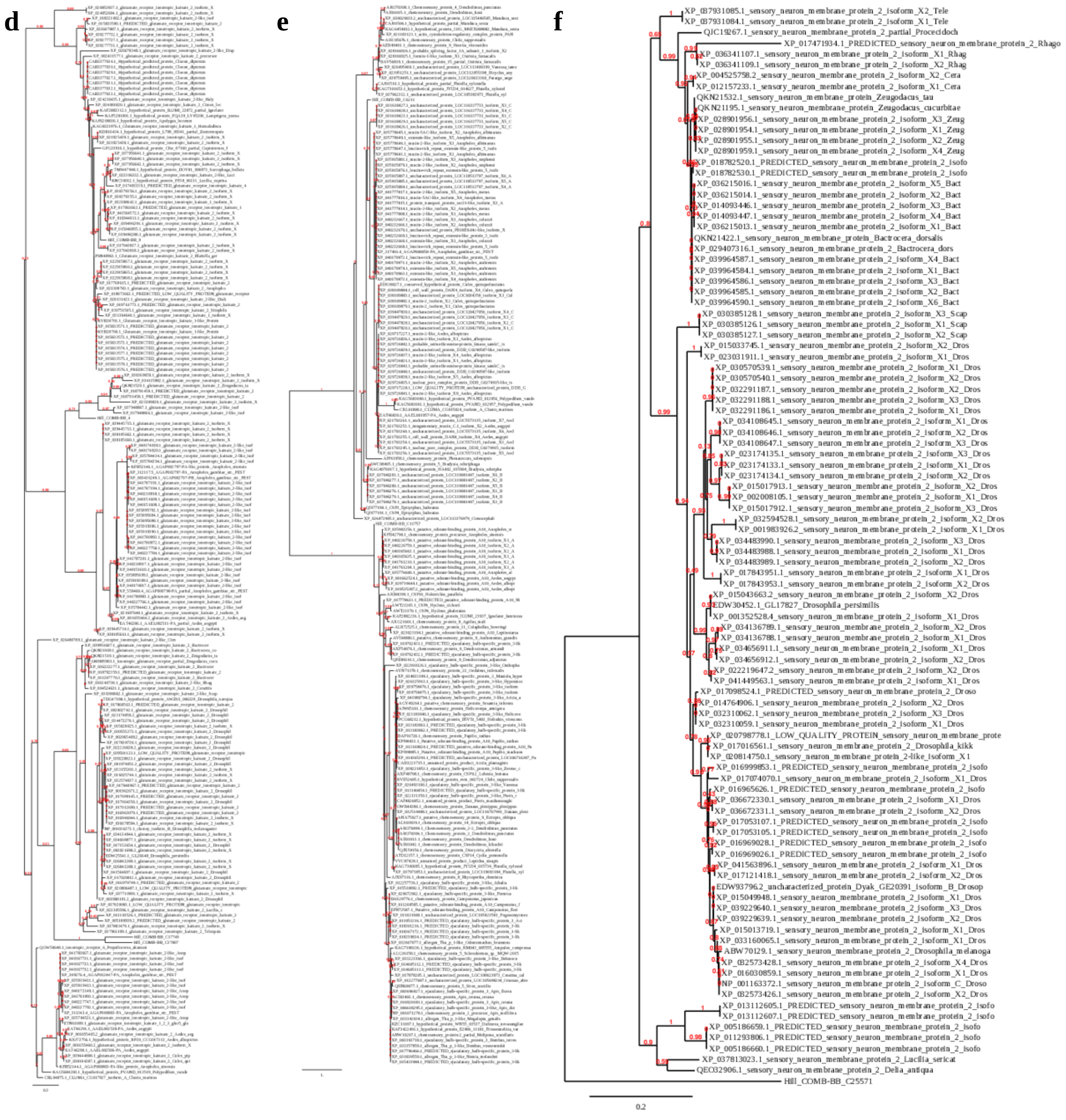
d
e
f
XP_037931085.1_sensory_neuron_membrane_protein_2_isoform_X2_Tele
XP_037931084.1_sensory_neuron_membrane_protein_2_isoform_X1_Tele
QJC19267.1_sensory_neuron_membrane_protein_2_partial_Procecidoch
XP_017471934.1_PREDICTED_sensory_neuron_membrane_protein_2_Rhago
XP_036341107.1_sensory_neuron_membrane_protein_2_isoform_X1_Rhag
XP_036341109.1_sensory_neuron_membrane_protein_2_isoform_X2_Rhag
XP_004525758.2_sensory_neuron_membrane_protein_2_isoform_X2_Cera
XP_012157233.1_sensory_neuron_membrane_protein_2_isoform_X1_Cera
QKN21532.1_sensory_neuron_membrane_protein_Zeugodacus_tau
QKN21195.1_sensory_neuron_membrane_protein_Zeugodacus_cucurbitae
XP_028901956.1_sensory_neuron_membrane_protein_2_isoform_X3_Zeug
XP_028901954.1_sensory_neuron_membrane_protein_2_isoform_X1_Zeug
XP_028901955.1_sensory_neuron_membrane_protein_2_isoform_X2_Zeug
XP_028901959.1_sensory_neuron_membrane_protein_2_isoform_X4_Zeug
XP_018782520.1_PREDICTED_sensory_neuron_membrane_protein_2_isofo
XP_018782530.1_PREDICTED_sensory_neuron_membrane_protein_2_isofo
XP_036215016.1_sensory_neuron_membrane_protein_2_isoform_X5_Bact
XP_036215014.1_sensory_neuron_membrane_protein_2_isoform_X2_Bact
XP_014093446.1_sensory_neuron_membrane_protein_2_isoform_X3_Bact
XP_014093447.1_sensory_neuron_membrane_protein_2_isoform_X4_Bact
XP_036215013.1_sensory_neuron_membrane_protein_2_isoform_X1_Bact
QKN21422.1_sensory_neuron_membrane_protein_Bactrocera_dorsalis
XP_029407316.1_sensory_neuron_membrane_protein_2_Bactrocera_dors
XP_039964587.1_sensory_neuron_membrane_protein_2_isoform_X4_Bact
XP_039964584.1_sensory_neuron_membrane_protein_2_isoform_X1_Bact
XP_039964586.1_sensory_neuron_membrane_protein_2_isoform_X3_Bact
XP_039964585.1_sensory_neuron_membrane_protein_2_isoform_X2_Bact
XP_039964590.1_sensory_neuron_membrane_protein_2_isoform_X6_Bact
XP_030385128.1_sensory_neuron_membrane_protein_2_isoform_X3_Scap
XP_030385126.1_sensory_neuron_membrane_protein_2_isoform_X1_Scap
XP_030385127.1_sensory_neuron_membrane_protein_2_isoform_X2_Scap
XP_015033745.1_sensory_neuron_membrane_protein_2_isoform_X2_Dros
XP_023031911.1_sensory_neuron_membrane_protein_2_isoform_X1_Dros
XP_030570539.1_sensory_neuron_membrane_protein_2_isoform_X1_Dros
XP_030570540.1_sensory_neuron_membrane_protein_2_isoform_X2_Dros
XP_032291187.1_sensory_neuron_membrane_protein_2_isoform_X2_Dros
XP_032291188.1_sensory_neuron_membrane_protein_2_isoform_X3_Dros
XP_032291186.1_sensory_neuron_membrane_protein_2_isoform_X1_Dros
XP_034108645.1_sensory_neuron_membrane_protein_2_isoform_X1_Dros
XP_034108646.1_sensory_neuron_membrane_protein_2_isoform_X2_Dros
XP_034108647.1_sensory_neuron_membrane_protein_2_isoform_X3_Dros
XP_023174135.1_sensory_neuron_membrane_protein_2_isoform_X3_Dros
XP_023174133.1_sensory_neuron_membrane_protein_2_isoform_X1_Dros
XP_023174134.1_sensory_neuron_membrane_protein_2_isoform_X2_Dros
XP_015017913.1_sensory_neuron_membrane_protein_2_isoform_X2_Dros
XP_002008105.1_sensory_neuron_membrane_protein_2_isoform_X1_Dros
XP_015017912.1_sensory_neuron_membrane_protein_2_isoform_X3_Dros
XP_032594528.1_sensory_neuron_membrane_protein_2_isoform_X2_Dros
XP_001983926.2_sensory_neuron_membrane_protein_2_isoform_X1_Dros
XP_034483990.1_sensory_neuron_membrane_protein_2_isoform_X3_Dros
XP_034483988.1_sensory_neuron_membrane_protein_2_isoform_X1_Dros
XP_034483989.1_sensory_neuron_membrane_protein_2_isoform_X2_Dros
XP_017843951.1_sensory_neuron_membrane_protein_2_isoform_X1_Dros
XP_017843953.1_sensory_neuron_membrane_protein_2_isoform_X2_Dros
XP_015043663.2_sensory_neuron_membrane_protein_2_isoform_X2_Dros
EDW30452.1_GL17827_Drosophila_persimilis
XP_001352528.4_sensory_neuron_membrane_protein_2_isoform_X1_Dros
XP_034136789.1_sensory_neuron_membrane_protein_2_isoform_X2_Dros
XP_034136788.1_sensory_neuron_membrane_protein_2_isoform_X1_Dros
XP_034656911.1_sensory_neuron_membrane_protein_2_isoform_X1_Dros
XP_034656912.1_sensory_neuron_membrane_protein_2_isoform_X2_Dros
XP_022219647.2_sensory_neuron_membrane_protein_2_isoform_X2_Dros
XP_041449563.1_sensory_neuron_membrane_protein_2_isoform_X1_Dros
XP_017098524.1_PREDICTED_sensory_neuron_membrane_protein_2_Droso
XP_014764906.1_sensory_neuron_membrane_protein_2_isoform_X2_Dros
XP_032310062.1_sensory_neuron_membrane_protein_2_isoform_X3_Dros
XP_032310059.1_sensory_neuron_membrane_protein_2_isoform_X1_Dros
XP_020798778.1_LOW_QUALITY_PROTEIN_sensory_neuron_membrane_prote
XP_017016561.1_sensory_neuron_membrane_protein_2_Drosophila_kikk
XP_020814750.1_sensory_neuron_membrane_protein_2-like_isoform_X1
XP_016999853.1_PREDICTED_sensory_neuron_membrane_protein_2_isofo
XP_017074070.1_sensory_neuron_membrane_protein_2_isoform_X1_Dros
XP_016965626.1_PREDICTED_sensory_neuron_membrane_protein_2_isofo
XP_036672330.1_sensory_neuron_membrane_protein_2_isoform_X1_Dros
XP_036672331.1_sensory_neuron_membrane_protein_2_isoform_X2_Dros
XP_017053107.1_PREDICTED_sensory_neuron_membrane_protein_2_isofo
XP_017053105.1_PREDICTED_sensory_neuron_membrane_protein_2_isofo
XP_016969028.1_PREDICTED_sensory_neuron_membrane_protein_2_isofo
XP_016969026.1_PREDICTED_sensory_neuron_membrane_protein_2_isofo
XP_041563896.1_sensory_neuron_membrane_protein_2_isoform_X1_Dros
XP_017121418.1_sensory_neuron_membrane_protein_2_isoform_X2_Dros
EDW93796.2_uncharacterized_protein_Dyak_GE20391_isoform_B_Drosop
XP_015049948.1_sensory_neuron_membrane_protein_2_isoform_X1_Dros
XP_039229640.1_sensory_neuron_membrane_protein_2_isoform_X3_Dros
XP_039229639.1_sensory_neuron_membrane_protein_2_isoform_X2_Dros
XP_015013719.1_sensory_neuron_membrane_protein_2_isoform_X1_Dros
XP_033160065.1_sensory_neuron_membrane_protein_2_isoform_X1_Dros
ABW70129.1_sensory_neuron_membrane_protein_2_Drosophila_melanoga
XP_032573428.1_sensory_neuron_membrane_protein_2_isoform_X4_Dros
XP_016030859.1_sensory_neuron_membrane_protein_2_isoform_X1_Dros
NP_001163372.1_sensory_neuron_membrane_protein_2_isoform_C_Droso
XP_032573426.1_sensory_neuron_membrane_protein_2_isoform_X2_Dros
XP_013112605.1_PREDICTED_sensory_neuron_membrane_protein_2_isofo
XP_013112607.1_PREDICTED_sensory_neuron_membrane_protein_2_isofo
XP_005186659.1_PREDICTED_sensory_neuron_membrane_protein_2_isofo
XP_011293806.1_PREDICTED_sensory_neuron_membrane_protein_2_isofo
XP_005186660.1_PREDICTED_sensory_neuron_membrane_protein_2_isofo
XP_037813023.1_sensory_neuron_membrane_protein_2_Lucilia_sericat
QEO32906.1_sensory_neuron_membrane_protein_2_Delia_antiqua
Hill_COMB-BB_C25571
0.2

Figure S2: Phylogenetic trees of larval odorant binding proteins (a), ionotropic receptors (b), gustatory receptors (c), glutamate receptors (d), chemosensory proteins (e), sensory neuron membrane proteins (f). Phylogenetic trees were built with Phylogeny Analysis software (http://www.phylogeny.fr/alacarte.cgi). Each sequence of *H. illucens* were manually checked with Blastx software. For each *H. illucens* sequence, the most similar sequences of different species or genus were selected. The corresponding amino acid sequences of all the selected sequences were subsequently aligned with MUSCLE software and then the phylogenetic tree was built, using default parameters. *H. illucens* sequences are reported as the contig number, while sequences of other species are reported with the accession number, the name of the protein. The Arabic numerals at the branching points are bootstrap values.

**Contig sequence**	**5' end**	**3' end**	**Signal peptide**	**Cystein pattern**
>41290<br>MARGTKATHDRCVAESGVTEEAIEEFSDGEIQEDEALKCYMYC<br>VFNATDVLHEDGEVHLEKILDSLPDSMHEIAVHMGKKCLCPKG<br>DTEWERAFWLHKCWKQADPKHYFFF	Open	Closed	Not evaluable	6 cysteines in the mature part of the protein.<br>The cysteine pattern is not conserved, except for 3 aminoacids between C2 and C3.<br>Being incomplete at 5 'it cannot be classified.
>42622<br>FRINFEYKEEFNNSGSLPDETDTTPMCYETDTTPMAQVLNNGII<br>DEFKVTSLLGPASGDSVEVCQAEGDEEQHPCKKAYAIAKCMMI	Open	Closed	Not evaluable	4 cysteines in the mature part of the protein.<br>The cysteine pattern is not conserved.<br>Being incomplete at 5 'it cannot be classified.
>41232<br>MNCFASVFVIVAALFAVSQANVNDPKLKSILEQCIGSEKASPAD<br>IAALEARSSDLSKEAKCVISCVMKNYKLLSDDGKVNRDVFMAE<br>AEEMTKGDAGAMKEAGEMFEICSAKTVADPCESAFNFGHCMK<br>TEMTARNIPMDF	Closed	Closed	0.9978	6 cysteines in the mature part of the protein.<br>The cysteine pattern is conserved.<br>Classic OBP.
>30834<br>MNRFASVFVIVAALAALSQADVNDPRLKASLEKCIGSEKASPA<br>DVEALKAHSSDLSREAQCVMACVMKEFKLLGDDGKINRDVY<br>MAEAEEMAKGDAGAIKQATEMYDICSAKTVADNCESANNFG<br>QCIKNEMIARNIPLDM	Closed	Closed	0.998224	6 cysteines in the mature part of the protein.<br>The cysteine pattern is conserved.<br>Classic OBP.
>54152	Open	Closed	Not evaluable	1 cystein in the mature part of the protein.

ALAKSSAFRLWFLYPDLSFSVWLIGAPTAEATAQISIRPRANVD NIFGQTENDKSGSKNPKRNAEDLASDANDEADVGMNLPEIMSI CKESFRINFEYKEEFNNSLSLPD				Being incomplete at 5 'it cannot be classified.
>42252 MVARGLILICAVAAAVGAPLSKTENDKSGPKNHKRNAEDSASA ANDEADVGMNLQEIMSSCNESFRINCEYMQEFNKSGSLPDETD KTPMCFMRCVLEQAQ	Open	Open	0.9774	4 cysteines in the mature part of the protein. The cysteine pattern is not conserved. Being incomplete at 3' it cannot be classified.
>18886 MGYLLKFFILVILSYSYGHKTQPRRDDEYPPKEMIRAIMTLHNI CVAKTGVTEEAIKEFSDGEIHEDEALKCYMNCLFHEAGLVNDD GQVDLEMLYAMLPDNLKEIALNMGKQCMTPQGDNLCEKAW WFHQCWKKADPKHYFLV	Closed	Closed	0.9274	6 cysteines in the mature part of the protein. The cysteine pattern is conserved. Classic OBP
>23028 QNKDKTEIRTEVTKCSDKKEQKSDDCTWAYRGFKCFLSKHLQ VVQSSVKP	Open	Open	Not evaluable	3 cysteines in the mature part of the protein. The cysteine pattern is not conserved. Being incomplete at 5’ and at 3’ it cannot be classified.
>2668 MSVRKVSGIAFAVGLLVLILAVDVQAKLDGKKVRAFTANIAKA CQPKTTPFGEIHEIIGKGKPTAEEKCFITCAITKGGLLSNNGEFQP DGIKKINEAMREFDDNPAEYKNIDEVIANCGGIEKPEKCDKGY AIAECSLKAFIDVHGNIF	Open	Closed	0.9301	6 cysteines in the mature part of the protein. The cysteine pattern is conserved. Classic OBP.
>2226 MKVLVLLAVIGVAAATYTIKTHDDLIKTRGLCVKELNVPDNYV EKFKKWDFQDDETTRCYIKCVLNKMELFDTANGFNVENLVEQ LGQNKDKTEVRTEVTKCSDKNEQKSDDCTWAYRGFKCFLSKH LQLVQSSVKS	Closed	Closed	0.9936	6 cysteines in the mature part of the protein. The cysteine pattern is conserved. Classic OBP.
>5246 MRSLFGLSFLLCVVFGTWVPKNREEICKDRKECIKEANLSKSNL DLVMQDAFGAEFPLNDELNRYIYCMGEKSELWEMGTMDANID RVYEVFHALGYDVNKDDLEKCFVPIFDVSYYVWAGRSIKCLW DKKLIRRKAGCSCGNEPTATGQHAK	Closed	Closed	0.9617	7 cysteines in the mature part of the protein. The cysteine pattern is not conserved. Plus OBP.
>16472	Closed	Open	0.9835	7 cysteines in the mature part of the protein.

MTKVRAVTLLLICNYAVLAQSSDSLTTWPKLIRYAKQCGGVDI<br>LKDGFMTSLNADRQQKCFISCVARKLGILTPDRKVNLKSARRV<br>FETLGLNPEQEKSAKAAIQDCKYITDADECEV				The cysteine pattern is not conserved.<br>Being incomplete at 3' it cannot be classified.
>11937<br>MKYFFVQNEASGGALCSSDFALFQVHAHWKPKTLEEWTQARA<br>ECFESEKVSLNCQEEIMSDEYPHNPGCYFRCIGLKTGTRDDKNG<br>YNINSGYETLMSTVWEVRKDSASPQIRRMMIRAGGLQLL	Closed	Closed	No	5 cysteines in the mature part of the protein.<br>The cysteine pattern is not conserved.<br>Being without signal peptide it cannot be classified.
>11394<br>MKLFLVFWTIFALVSAEWVPRTSDQMYKDQAECFKQLELTEEE<br>QQKVKKEDFPDEPKFRCYLRCILMGGQIWDDEKGYNPERAYA<br>ELLNIDMTADVENLRKCNTQNLHHSDSCTRAFRVVKCFANNN<br>YITSIKPKS	Closed	Closed	0.999	6 cysteines in the mature part of the protein.<br>The cysteine pattern is conserved.<br>Classic OBP.
>1500<br>MTTFRKNTIFTFALVLFIYGLAVTSVKAELDEARAKEFFIKVASE<br>CITDDIKEDDIEALMKDDSASHEGKCLIACLMKKLGVLDENGE<br>LSPAGVNDIREKMQSFGGDSEKGHQIATAILEKCSNLKEEDECE<br>TAYQIHQCARGEIKAIRG	Closed	Closed	0.8226	6 cysteines in the mature part of the protein.<br>The cysteine pattern is conserved.<br>Classic OBP.
>17009<br>TLFRSGKLFLILVAIILIGITEALKCRNNDGPSEEKMKRVARSCM<br>KKVSNVQDGDDSSDYYEDRGGFDRGNNSQKRGRDQLGERRH<br>SRVLSRDDYGRQRYNNHFGNLRNPGTSERHQHQVNSGPNRHS<br>NDKISYGNRGNQNDKSCVVHCFFEELNMLNNQDFPDKHKVTY<br>VITRDIRDNELREFYADSIQECFRFIEAERQRDKCQFSKDLINCM<br>AEYARANCEDWYNHTMIFNN	Open	Closed	0.9967	7 cysteines in the mature part of the protein.<br>The cysteine pattern is not conserved.<br>Plus OBP.
>74693<br>MKTLAIFLVICIVGAFASTLTDDQKAKLREHRDACIAETGVDKA<br>VIDGITKGGPITRDSKLDCYSACLLKKIKIMKDDGTIDLEATRAK<br>AATVNADPEKVKKVIEKCKDLTGKDTCEKGGNILACFIENKEF<br>PVLN	Open	Closed	0.9984	6 cysteines in the mature part of the protein.<br>The cysteine pattern is conserved.<br>Classic OBP.
>4062<br>MEFETTKALLKEGNHPYNNNNNRWRNNPCWIRALKCERAFW<br>LHKCWKQADPKHYFIFEKIRRHRWT	Closed	Open	Not evaluable	3 cysteines in the mature part of the protein.<br>The cysteine pattern is not conserved.<br>Being incomplete at 3' it cannot be classified.

>17957<br>MAKLGLTSILLVIFVCISAGLDEDPISIVTEVPDDSELRVIAKKCK<br>IPGV<br>SDEEIERMLNDDTNLSYGGKCTLKCIAENIGVVIKDDQMDIAET<br>KKIVEESAVDKEELAMPFYLIDKCGEIKDKDECELAYKLNLCMI<br>EATKEWNSNSTSDDLL	Closed	Closed	0.9942	6 cysteines in the mature part of the protein.<br>The cysteine pattern is conserved.<br>Classic OBP.
>75784<br>MPDLIPVATEAIEKCGEIIRAKMEKGKDRKPQGDDQCHHVPSM<br>YAGCFFAQTTINCPDSAWQDTDDCNRAREFMKSCPMPHRPKE	Open	Closed	Not evaluable	6 cysteines.<br>The cysteine pattern is not conserved.<br>Being incomplete at 5' it cannot be classified.
>61546<br>MFRDLSVFLVCVFVVQCLADPDCSEPPKKKDFMGCCKKPEALS<br>EIITKCSEKYPFEHRRPNICYGECVFNETGLAVDGVIQRDKISSVI<br>GEFYKDMP	Open	Open	0.9992	8 cysteines.<br>The cysteine pattern is not conserved.<br>Being incomplete at 5' and 3’ it cannot be classified.
>43028<br>MPLPTTYFRGCLFLFLVAVFAEDHCSKPPPKFAPRECCEPVKWS<br>ESNVEDKCAGAPSAVCFTECIFNSTDIIQNDGPNATNIRTFFEIEL<br>KADPDYIPIAINNFEMCYSLVKRRMEDKDHQIDGCDTFPALIME<br>CTIAQMFVDCPSEKWKSSEACELARKYYKRCPDRHFTTNA	Open	Closed	0.9971	12 cysteines.<br>The cysteine pattern is not conserved.<br>Atypical OBP.
>73118<br>MNAVQTCYTFVTQKMKNKNEKVGGCSVLPSLVSECTDTQIFV<br>NCPANKWKSNQLCDSAKAYLKKCPVLNFPMNN	Open	Closed	Not evaluable	6 cysteines.<br>The cysteine pattern is not conserved.<br>Being incomplete at 5' it cannot be classified
>56633<br>LEALQKDVTCVAECVGKKFGVLDEAGNVKHDVFLAHLKTKV<br>QDSEWKLKVSDSIAEKCIEETKKEVEGHIAKRDVTSQKACNPTS<br>LKISQCLWREYVMACPKDLQVDSPKCNKLREKIDKGDAV	Open	Open	Not evaluable	6 cysteines.<br>The cysteine pattern is not conserved.<br>Being incomplete at 5' and 3’ it cannot be classified.
>12724<br>VQIVADPSCLNPPPNNFITSCCVRNGHLRDIVAKCNEMIPAETA<br>ARSFCHAECVFNETGLSANNVIQHDKMVEVTKDLFKDTTEFMP<br>VIDAAVKKCKGVAEAKLVKINEIKLDGADKCHPLPALIMSCFL<br>AELFINCPASLWQNTEECTQAKMFMTVCIMN	Open	Closed	Not evaluable	11 cysteines.<br>The cysteine pattern is not conserved.<br>Plus OBP.
>32770	Closed	Closed	0.998	6 cysteines in the mature part of the protein.

MKVFIVLFACIILGSADDWKPKSKEEALQIREECLKLNNVPAPL VEKVKKFEYEDEESVRCHIKCTAEKVGVWDNVKGYDVDRLY DQVVIKEEVATNKDDLKKCIDDKHDGEDDCTWAYRNFKCML DNKYLTKKA				The cysteine pattern is conserved. Classic OBP.
>410 MKILISLFAIFVLVTADWEPRNREQFIKDRDECFKSENLSEHCID EIKRRIFPHEPKCYFRCVLMKNSVWDDTSGYDVEKAYKELTHN GLEIAKEDLNKCNSDEMKNKDPCVWANNIIKCIWDYLPKKKQ D	Closed	Closed	0.9957	7 cysteines in the mature part of the protein. The cysteine pattern is conserved. Plus OBP.
>56175 ASAFNKEQFLKNREECLKSEKVPEAVIEKLKNRQYGEDLGHEA KCYIRCLALKSGTWDDAKGYDLEKAYEDLQSAGLEVSKENLK KCISSNPDNDDKCTWAAKDLKCLWTNKYISRSEER	Open	Open	Not evaluable	6 cysteines in the mature part of the protein. The cysteine pattern is conserved. Classic OBP.
>23172 MKFFIVLCAVIAMAAAQWTPKSKEELLKFREECFKSENVPQAV IEKLNNRQYDEDLGQEARCYIRCIGLKSEIWDDTNGYDLERSY QNLISTGFEVSKENLQKCITPNTDNDDKCTWAAKNLKCLWTN KYVTKKQ	Open	Closed	0.9985	6 cysteines in the mature part of the protein. The cysteine pattern is conserved. Classic OBP.
>8085 MKLFVVLAAIVAFASAEWATRSPAELAQSRLSCIQELSVPANLV EQIKKFQYPDEEIVRCYIRCTSEKIGIWSDDSGFITDRVIEQLAGN RDKDAFRADVLKCIDSNEQKSDKCTWAYRNFNCFIKNNLILVQ SQINASS	Closed	Closed	0.9986	6 cysteines in the mature part of the protein. The cysteine pattern is conserved. Classic OBP.
>1903 MKLLIALCTVFALVQAWAPSTFEEYLNYRKECFVSENISKDDQ DLLNKGLYPTKPGCYYRCNGLKTGIWDDVNGYSVDRGYELLT AEGWEVTKDSLRKCNTPDKKDADLCKWSAAVAKCLWGNVLI KRKDA	Closed	Closed	0.9995	6 cysteines in the mature part of the protein. The cysteine pattern is conserved. Classic OBP.
>32259 MKLFIALCAVIAVASAAFNKEEFLKMREECLKSEKVPAAIIEKL KHREYEQDLGHEAKCYIRCMGLKTGVWDDTHGYNVDKTYDD FHSAGLEVSKDNLKKCFTSHGDDDKCVWAAKDVKCLWTNKY VTIKKDLFE	Open	Closed	0.9978	6 cysteines in the mature part of the protein. The cysteine pattern is conserved. Classic OBP.
>11523 MKFFVASCVLLAVASAQWAPKTGKEVAQIREDCVKEENVPES LAEGLKKFEYPDEEPIRCYVKCVSAKLGVWNDETGFDADRVA	Closed	Closed	0.9986	6 cysteines in the mature part of the protein.

DQVKQDRNKDDIKAEVEKCIDKNEQNSDKCTWAYRNLKCVM<br>DKKLLQVESLIAN				The cysteine pattern is conserved.<br>Classic OBP.
>3768<br>MKLLIFLFAIFVLVAADWKPRSREQYTKDGDECFKSENISEDGIHEIRR<br>HVFTDDSKCFFRCVLMKNHVWDDTTGYNVERVYKEVTHIGLKASKD<br>GLTQCNSDDKKDKDPCQWVNNIVRCVFEHNYIEPNY	Open	Closed	0.9972	6 cysteines in the mature part of the protein.<br>The cysteine pattern is conserved.<br>Classic OBP.
>2731<br>MKCSLVCFLLVFAIASGSDWEPKGNEELVSIRDECFKLENVSEE<br>SAAKILKNEYPDEPSVHCYVRCTSAKVGTWDDEAGPDIDRTLR<br>QIQKSGNTITREELVRCKPEKQENKCLWAYKGLMCILKSEGIKD<br>DLIK	Closed	Closed	0.9987	6 cysteines in the mature part of the protein.<br>The cysteine pattern is conserved.<br>Classic OBP.
>1002<br>MKFFIVLCAIIAMASAISTEEFQKMREECFKSEKVPEADIEKLKN<br>REYGLDLGHEAKCYIRCLGMKTGNWDDTNGYDVEKIYTDFRT<br>AGLEVTKENLNKCFKSSGDDDKCVWAAKDLKCLWTNKYISRK<br>Q	Open	Closed	0.9993	6 cysteines in the mature part of the protein.<br>The cysteine pattern is conserved.<br>Classic OBP.
>1003<br>MKFFIVLCAIIAMAFAISTEEFQEMREECFKSEKVPEADIEKLKH<br>REYGLDLGHEAKCYIRCLGMKTGNWDDTNGYDVEKIYTDFRT<br>AGLEVTKENLKKCFKSSGDDDKCVWAAKDLKCLWTNKYISRK<br>Q	Open	Closed	0.9988	6 cysteines in the mature part of the protein.<br>The cysteine pattern is conserved.<br>Classic OBP.
>32362<br>FIIFWAIFAIAAADWKPPTMEELAKYREECIKEEKVPEQHAEGIR<br>KHGYFETVNIDSCFIHCMALKTGIRDKGPGYNLDRVYETLKVL<br>NKEVSKSDLYDMTFSMNRNLCAEDAVGMKRIWNKLQEAKEE<br>RESRMHPTECGIVHHSRLAYRRIHIGEKPS	Open	Closed	Not evaluable	5 cysteines in the mature part of the protein.<br>The cysteine pattern is not conserved.<br>Minus OBP.
>12769<br>MKFLVVFCAVLVLVHAVWKPMTSEEWAKHRAECFESEKISQH<br>CQEEIKRGEFPPEPGCYFRCIGLKAGMWDDTHGFNMERSHDSL<br>TANGWEVDKDNLKKCYSANQKDNDPCKWAAAIAKCMFDNE<br>YLKKKA	Closed	Closed	0.9933	6 cysteines in the mature part of the protein.<br>The cysteine pattern is conserved.<br>Classic OBP.
>5932<br>MKLFIILFAIMATASAIPKEKYLKYREECFKSEKVPAVVIEKLNN<br>PQYEEDMGHEAKCFIRCMALKIGSWDDTNGYNIDKTYADFQD<br>GGLEVSKENMKKCFTSNPDNDDKCVWADKDLKCLYRNKYVT<br>HKYSIN	Closed	Closed	0.9989	6 cysteines in the mature part of the protein.<br>The cysteine pattern is conserved.<br>Classic OBP.

>9042<br>LVQADWKTKAPEDLIKDRLECFESEKVSHHCQEQIKKGEFPHE<br>PGCYYRCIGLKSGIWDDTNGFNIERGIASFEATGWVVEKDNLK<br>KCTTDDQKNDDPCKWSAAIAKCLFDNKYLKRKA	Open	Closed	Not evaluable	7 cysteines in the mature part of the protein.<br>The cysteine pattern is not conserved.<br>Plus OBP.
>70911<br>MATAYTKEDFLKDREECLKSEKVPEAVIEKLKNRQYDGDLGH<br>EAQCYIRCLAVKVGSFDDATGYDLDKTYSHLTSAGFVVTKENL<br>KKCISAAPADGDKCAWAAKDLKCLWTNKYISKKQ	Open	Closed	Not evaluable	6 cysteines in the mature part of the protein.<br>The cysteine pattern is not conserved.<br>Being incomplete at 5' and 3’ it cannot be classified.
>8987<br>MKSLIIFCAIFAVVFANWSTPTKEQFKQHRDDCLKEGNVPEETA<br>NKIRKEQYPNDRDTYCYIRCVGSKSGIWNDRKGYDIDRALQVF<br>EANGYEVTRENLERCFAPLPGADTCTWAGVNMRCLRDNKYVT<br>KKASA	Closed	Closed	0.9973	6 cysteines in the mature part of the protein.<br>The cysteine pattern is conserved.<br>Classic OBP.
>7966<br>MKVLLVFCAVLALVYADWKPKTLEEWVKNRAECFESEKVSQ<br>HCQEEIKRGEYPHEPGCYFRCIGTKTGIWHDTNGFDIEKGHEAL<br>VATGWEVEKDNLKKCTSANPKEDDPCKWSAAIAKCMFDNKY<br>LKRKE	Closed	Closed	0.9994	7 cysteines in the mature part of the protein.<br>The cysteine pattern is conserved.<br>Plus OBP.
>61431<br>MKLFIVFWAIFAIATADWKPPTMEELSKYREECIKEEEVPEREA<br>EEIRKHDYFENVPLNHCFIQCMGEKTGIRESRRTFDLDRVYETF<br>KVLNNELSRKDFDENHFDARRSGCIEDGIYMRFIWSTIQERSGL<br>K	Open	Closed	0.9987	4 cysteines in the mature part of the protein.<br>The cysteine pattern is not conserved.<br>Minus OBP.
>17801<br>SLIIPHICIYLLQVTDEQMKKSAQMLRDVCQPKFKISDEVANGIK<br>DGIIPDEQNVKCYINCVLEMMNSIKKGKLNYEASVKQIDLLLPD<br>RLKDSFKAGLAACRNSIDGIRNHCEAATVLLKCLKANIPEFFFP	Open	Closed	Not evaluable	6 cysteines in the mature part of the protein.<br>The cysteine pattern is conserved.<br>Classic OBP.
>76148<br>DDIMSGKKFTPSRELDCYAACIFKKNGMMKPDGTIDQDKPSKT<br>EEAKECKKLSGADECETAGKVMGCFAKHNLIPKM	Open	Closed	Not evaluable	5 cysteines in the mature part of the protein.<br>The cysteine pattern is not conserved.

Table S4. analysis of the OBP sequences from adult transcriptome, searching for complete sequence at 5’ and 3’ ends, the presence of the signal peptide with SignalP-5.0 software (http://www.cbs.dtu.dk/services/SignalP/) and the conserved cysteine pattern. In some cases, although the Expasy-Translate Tool

software highlights the longest frame beginning with a methionine, comparing the sequence with similar proteins in known databases (NCBI protein database), it was possible to indicate the correct frame. In some cases. the frame is not complete at 3’ or at 5’ ends and comparing it with similar *H. illucens* proteins or with similar sequences in known protein databases (NCBI protein database), it is possible to confirm that the sequence from the transcriptome is or is not complete and if it includes or not includes the entire mature peptide.

**Contig sequence**	**5’ end**	**3’ end**	**Signal peptide**	**Cystein pattern**
>21691<br>MNCFASVFVIVAALVAVSQANVNDPKLKSILEQCIGSEKASPADIAALEARSSDLSKEAKCVISCVMKNYKLLSDDGKVNRDVFMAEAEEMTKGDAGAMKEAGEMFEICSAKTVADPCESAFNFGHCMKTEMTARNIPMDF	Closed	Closed	0.9978	6 cysteines in the mature part of the protein.<br>The cysteine pattern is conserved.<br>Classic OBP.
>13368<br>MNRFASVFVIVAALAALSQADVNDPRLKASLEKCIGSEKASPADVEALKAHSSDLSREAQCVMACVMKEFKLLGDDGKINRDVYMAEAEEMAKGDAGAIKQATEMYDICSAKTVADNCESANNFGQCIKNEMIARNIPLDM	Closed	Closed	0.9982	6 cysteines in the mature part of the protein.<br>The cysteine pattern is conserved.<br>Classic OBP.
>57<br>MKVLVLLAVIGVAAATYTIKTHDDLIKTRGLCVKELNVPDNYVEKFKKWDFQDDETTRCYIKCVLNKMELFDTANGFNVENLVEQLGQNKDKTEVRTEVTKCSDKNEQKSDDCTWAYRGFKCFLSKHLQLVQSSVKS	Closed	Closed	0.9936	6 cysteines in the mature part of the protein.<br>The cysteine pattern is conserved.<br>Classic OBP.
>11107<br>MKLFLVFWTIFALVSAEWVPRTSDQMYKDQAECFKQLELTEEEQQKVKKEDFPDEPKFRCYLRCILMGGQIWDDEKGYNPERAYAELLNIDMTADVENLRKCNTQNLHHSDSCTRAFRVVKCFANNNYITSIKPKS	Closed	Closed	0.999	6 cysteines in the mature part of the protein.<br>The cysteine pattern is conserved.<br>Classic OBP.
>31956<br>MSFPKINSVVIAVILSALVLVVVRAKVDENKLKAYTANIAKTCQPEGEPFGEVHDIVEKANPTQDEKCFITCTMTKWGLLSENGKFQPDGVRKVNEAIREFDDNPAEYKNADEAIIAKCSAIEKPEKCDKGYAIAECGFKVFDEIHG	Closed	Closed	0.8483	6 cysteines in the mature part of the protein.<br>The cysteine pattern is conserved.<br>Classic OBP.

>4812<br>MTKILIIATVFCAALTAAYDFKDTAFNEVILKDLLDLTDGSDSS<br>VLHRIARAAEGAADGEECKGRKKDRGVHCCTDKIDPKQLESIK<br>AAKKECLAELHGNDSDVYFKFDPFTCEKLEELKKDVTCVAEC<br>VGKKFGVLDEAGNVKHDVFLAHLKTKVQDSEWKLKVSDSIAE<br>KCIEETKKEVEGHIAKRDVTSQKACNPTSLKISQCLWREYVRA<br>CPKDLQVDSPKCNKLREKIEKGDAVSYKGFYLRHLNDDK	Closed	Closed	0.9918	12 cysteines in the mature part of the protein.<br>The cysteine pattern is not conserved.<br>Atypical OBP.
>7755<br>MAKILIVVAIASLCAAISTAYDFKDSAFNEVIFRDLLDLTDDDD<br>NSVLHREVRTADTAAIRVDPVPGTECKEKRRDRGFYCCNDKV<br>DLNQLEIIRGVRKECLAELHGNDSDIYFKFDPFTCEKLEELKKD<br>VTCIAECVGKKFGLLDDSGNIKPDIFLAYLKMKAKDSEWKLQV<br>TDEIAGKCIEDTRKEVEHHLAERDLTSGKICNPSSLKISQCLWRE<br>YVRACPKNLQTDSPKCCKLREKIEKGDAVTYKGFYLRHLNDD<br>K	Open	Open	0.9898	14 cysteines in the mature part of the protein.<br>The cysteine pattern is not conserved.<br>Atypical OBP.
>31807<br>MFRNLSVFLVCAFAVQCLADPDCSQRPKKEDFMNCCAKPAPF<br>KDVMAKCNDKFPFQKGEPNFCHGECVFNETGISVNGVIQKDKI<br>SAILGEMYKAMPDFIPVATKAVEKCDETVRTKMAKIMEHKPEG<br>ADKCNPVPAVYGFCIFVETMVNCPASGWQNTDDCNRAREFMK<br>SCPMHHKQ	Closed	Closed	0.9996	12 cysteines in the mature part of the protein.<br>The cysteine pattern is not conserved.<br>Atypical OBP.
>42512<br>MFRDLSVFLVCVFVVQCLADPDCSEPPKKKDFMGCCKKPEALS<br>EIITKCSEKYPFEHRRPNICYGECVFNETGLAVDGVIQRDKISSVI<br>GEFYKDMPDLIPVATEAIEKCGEIIRAKMEK	Open	Open	0.9992	7 cysteines in the mature part of the protein.<br>Being incomplete at 3’ it cannot be classified.
>51370<br>MHPLELSVFIFGVFVSTTFAAEPNCAQPPPPANIQQCCNNQVFA<br>ELAPKCVSKIPPATAGMPRPIGMCFGECMFNETGLSVNGQIQLD<br>KAKSILTEKFKAIPEFVAPFEQALTKCDKIIKEKMASGKPPANM<br>PPIPCNAMADIIASCTMLEAFINCPDSKWQNNEACNNAKQYFKS<br>CPMPPPPKA	Closed	Closed	0.9977	12 cysteines in the mature part of the protein.<br>The cysteine pattern is not conserved.<br>Atypical OBP.

>45961<br>MPLPTTYFRGCLFLFLVAVFAEDHCSKPPPKFAPRECCEPVKWS<br>ESNVEDKCAGAPSAVCFTECIFNSTDIIQNDGPNATNIRTFFEIEL<br>KADPDYIPIAINNFEMCYSLVKRRMEDKDHQIDGCDTFPALIME<br>CTIAQMFVDCPSEKWKSSEACELARKYYKRCPDRHFTTNA	Open	Closed	0.9971	12 cysteines in the mature part of the protein.<br>The cysteine pattern is not conserved.<br>Atypical OBP.
>441<br>MKFLIVLCAVLALVQADWKTKAPEDLIKDRLECFESEKVSHHC<br>QEQIKKGEFPHEPGCYYRCIGLKSGIWDDTNGFNIERGIASFEAT<br>GWVVEKDNLKKCTTDDQKNDDPCKWSAAIAKCLFDNKYLKR<br>KA	Open	Closed	0.9997	7 cysteines in the mature part of the protein.<br>The cysteine pattern is conserved.<br>Plus OBP.
>758<br>MKLLIVLCAVIAMACAVSKEEFLKIREEGLKSENVPQAVIEKLK<br>NREYGGDLGHEAKCYIRCLGLKTGNWDDTNGYDVDKVFADF<br>NGAGLEVTKENLKKCFKSSGDDDKCVWAAKDLKCLWTNKYI<br>SRKQ	Closed	Closed	0.9979	5 cysteines in the mature part of the protein.<br>The cysteine pattern is conserved.<br>Minus OBP.
>768<br>MKFFVASCVLLAVASAQWAPKTGKEVAQIREDCVKEENVPES<br>LAEGLKKFEYPDEEPIRCYVKCVSAKLGVWNDETGFDADRVA<br>DQVKQDRNKDDIKAEVEKCIDKNEQNSDKCTWAXXNLKCVM<br>DKKLLQVESLIAN	Closed	Closed	0.9986	6 cysteines in the mature part of the protein.<br>The cysteine pattern is conserved.<br>Classic OBP.
>1173<br>MKSLIIFCAIFAVVFANWSTPTKEQFKQHRDDCLKEGNVPEETA<br>NKIRKEQYPNDRDTYCYIRCVGSKSGIWNDRKGYDIDRSLQVF<br>EANGYEVTRENLERCFAPLPGADTCTWAGVNMRCLRDNKYVT<br>KKASA	Closed	Closed	0.9973	6 cysteines in the mature part of the protein.<br>The cysteine pattern is conserved.<br>Classic OBP.
>1844<br>MKILISLFAIFVLVTADWEPRNREQFIKDRDECFKSENLSEHCID<br>EIKRRIFPHEPKCYFRCVLMKNSVWDDTSGYDVEKAHKELTHN<br>GLEIAKEDLNKCNSEEMKNKDPCVWANNIIKCIWDYLPKKKQE	Closed	Closed	0.9957	7 cysteines in the mature part of the protein.<br>The cysteine pattern is conserved.<br>Plus OBP.
>2633<br>MKFFIVLCAIIAMAFAISTEEFQEMREECFKSEKVPEADIEKLKH<br>REYGLDLGHEAKCYIRCLGMKTGNWDDTNGYDVEKIYTDFRT	Closed	Closed	0.9988	6 cysteines in the mature part of the protein.<br>The cysteine pattern is conserved.

AGLEVTKENLKKCFKSSGDDDKCVWAAKDLKCLWTNKYMSR KQ				Classic OBP.
>3948 MKFFIVLCVVIAMAAAQWTPKSKEELLKFREECFKSENVPQAV IEKLNNRQYDEDLGQEARCYIRCIGLKSEIWDDTNGYDLERSY QNLISTGFEVSKENLQKCITPNTDNDDKCTWAAKNLKCLWTN KYVTKKQ	Open	Closed	0.9987	6 cysteines in the mature part of the protein. The cysteine pattern is conserved. Classic OBP.
>3962 MKFIIVFCAVIAMAAALWTPTTKEEFLKQREECFKSENVPQAVI EKLNNRQYDEDLGPEAKCYIRCLGLKSGTWDDTNGYDLEKSY ENLLSSGFEVTKENLKKCITSNPDNDDKCTWAAKDLKCLWTH KYITKKQ	Open	Closed	0.9902	6 cysteines in the mature part of the protein. The cysteine pattern is conserved. Classic OBP.
>3982 MKCSLICFLLVFAIASGSDWEPKGNEELVSIRDECFKLENVSEES AAKILKNEYPDEPSVHCYVRCTSAKVGTWDDEAGPDIDRTLRQ IQKSGNTITREELVRCKPEKQENKCLWAYKGLMCILKSEGIKDD LIK	Open	Closed	0.9987	6 cysteines in the mature part of the protein. The cysteine pattern is conserved. Classic OBP.
>4168 MKILISLFAIFVLVTADWKPRNKEQLIKDRDECFKSENLSEHCID EVKRRIFPDEPKCHFRCFLMKNSVWDDTSGYDVEKAYKELTH NGFELSKEDLNKCNSEEMKNKDSCVWAGNAVKCTWDYLPKK KQD	Closed	Closed	0.9925	6 cysteines in the mature part of the protein. The cysteine pattern is conserved. Classic OBP.
>5161 MKILISLFAIFVLVTADWKPRNREQLIKDRDECFKSENLSEHCID EIKRRIFPDEPKCHFRCFLMKNTVWDDTSGYDVEKAYKELTHN GFELSKEDLNKCNSEVMKNKDPCVWAGNAVKCTWDYLPKKK QD	Closed	Closed	0.9918	6 cysteines in the mature part of the protein. The cysteine pattern is conserved. Classic OBP.
>5826 LKIREECLKSEKVPEAVIEKLKNREYGQDLGHEAKCYIRCLGLK TGIWDDTHGYNVDKAYDDFHSAGLEVSKDNLKKCFISTGDDD KCVWAAKDLKCLWTNKYATKKE	Open	Closed	Not evaluable	6 cysteines in the mature part of the protein. Being incomplete at 5’ and 3’ it cannot be classified.
>7404 MKYIIVLCAVIAMAAALWTPTTKKEFLKQREECFKSENVPQAV IEKLNNRQYDEDLGPEAKCYIRCLGLKSGTWDDTNGYDLEKSY	Open	Closed	0.9822	6 cysteines in the mature part of the protein. The cysteine pattern is conserved.

ENLLSSGFEVTKENLKKCITSNPDNDDKCTWAAKDLKCLWTH KFITKKQ				Classic OBP.
>7621 MKLLIGLCAMIAMVASQWSPSTKIDYLKFRDECFKSENVPQSAI DKLNNEQYGEDLGHEAKCYIRCLGVKTGTWDDTKGYDIEKGY QNLISWGFEVNKENLQKCATPNTENDDKCVWAAKNTKCLWT NKYVTKKQ	Open	Closed	0.9978	6 cysteines in the mature part of the protein.<br>The cysteine pattern is conserved.<br>Classic OBP.
>9011 MKLFVVLAAIVAFASAEWATRSPAELAQSRLSCIQELSVPANLV EQIKKFQYPDEEIVRCYIRCTSEKIGIWSDDSGFITDRVIEQLAGN RDKDAFRADVLKCIDSNEQKSDKCTWAYRNFNCFIKNNLILVQ SQINASS	Open	Closed	0.9986	6 cysteines in the mature part of the protein.<br>The cysteine pattern is conserved.<br>Classic OBP.
>13738 MKLLIFLFAIFVLVAADWKPRSREQYTKDGDECFKSENISEDGI HEIRRHVFTDDSKCFFRCVLMKNHVWDDTTGYNVERVYKEVT HIGLKASKDGLTQCNSDDKKDKDPCQWVNNIVRCVFEHNYIEP NY	Closed	Closed	0.9972	6 cysteines in the mature part of the protein.<br>The cysteine pattern is conserved.<br>Classic OBP.
>14606 MQLFIILFAMVAMASAIPKEKYLKYREERFKSEKVPAVVIEKLN NPQYEEDMGHEAKCFIRCMALKIGSWDDTNGYNIDKTYADFQ DGGLEVSKENMKKCFTSNPDNDDKCVWADKDLKCLYRNKYV THKYSIN	Closed	Closed	0.9968	5 cysteines in the mature part of the protein.<br>Minus OBP.
>15984 QNEASGGVCAVLILVHAHWKPKTLEE- TQACTECSESEKVSLNCQEEIMSDEYPHNPGCYFRCIRLKTGT- DDTNGYNIDSDYETLMSTVWEVRKDNVKECDSADKKDDDPC RWSAAVAKCLFDNRYRRRSKRIG	Open	Closed	Not evaluable	9 cysteines in the mature part of the protein.<br>Due to the inserted premature stop codon it cannot be classified.
>29339 MKFLIVLCAVIAMACAITKEQVLMYREECFESEKVPEAVIEKLK NREYGEDLGHEAKCYIRCLGLKTGTWDGTHGYDIEKTYEHMT EAGLVGTKENLKKCFKAS	Open	Open	0.997	4 cysteines in the mature part of the protein.<br>Being incomplete at 3’ it cannot be classified.

>171<br>MKYFLLFLTLLPVALATWVPHTKEDFLKYRLDCYKESNITEAN FEKIKKHQFAHDHDTYCYIHCLGLKLGIWDELKGYDVDRVYET LKVHNLDVKKEDLSKCFHQVIDADTCTWSAINWMCLWERKYI TKKN	Closed	Closed	0.9985	6 cysteines in the mature part of the protein.<br>The cysteine pattern is conserved.<br>Classic OBP.
>363<br>MKYFLLFLTLLPVAFATWVPHTKEDFLKYRLDCYKESNITEAN FEKIKKQQFAHDHDTYCYIHCLGLKLGIWDELKGYDVDRVYET LKVHNLDVKKEDLSKCFHQVMDADTCTWSAINWMCLWERKY ITKKN	Open	Closed	0.9984	6 cysteines in the mature part of the protein.<br>The cysteine pattern is conserved.<br>Classic OBP.
>1936<br>MKSLIVFCAILGLVYADWKPKTREEWSRHRVECFESEKVSHHC QEQVKKGEYAHESGCYYRCIGLKVGFWDDTNGFNNDRGDEAF TATGWEVEKENLKKCASADQKDDDPCKWSLIVAKCLIENKYL KLKA	Open	Closed	0.999	6 cysteines in the mature part of the protein.<br>The cysteine pattern is conserved.<br>Classic OBP.
>13737<br>MKLLIFLFAIFVFVAAHWEPRSKEQHDKDGEACFKSEVIHGDMI DYETKCFLRCVLMKNNVWNDTTGYNVERAYNEATHIGLRASK EGLTECNSDDKKDKDPCLWVNNILRCAYEHNYMKPNF	Open	Closed	0.9982	6 cysteines in the mature part of the protein.<br>The cysteine pattern is conserved.<br>Classic OBP.
>3485<br>TAAYDFKDSAFNEVILKDLLDLTDGSDSSVLHRIARAAEGAAE GEECKGRKKDRGVHCCSDKIDPKQLESIKAAKKECLAELHGND SDVYFKFDPFTCEKLEELKKDVTCVAECV	Open	Open	Not evaluable	7 cysteines in the mature part of the protein.<br>Being incomplete at 5’ and 3’ it cannot be classified.
>59460<br>MAKLGVTSILLVIFVCVSAGLDEDPISIVTEVPDDSELRVIAKKC KIPGVSDEEIERMLNDDTNLSYGGKCTLKCIAENIGIVIKDDQID VAATKKIVEKSAVDKEELAMPFYLIDKCGEIKDKDECELAYKL NLCMIEATKEWNSNSTSDDLL	Open	Closed	0.9948	6 cysteines in the mature part of the protein.<br>The cysteine pattern is conserved.<br>Classic OBP.

Table S5. Analysis of the OBP sequences from larval transcriptome, searching for complete sequence at 5’ and 3’ ends, the presence of the signal peptide with SignalP-5.0 software (http://www.cbs.dtu.dk/services/SignalP/) and the conserved cysteine pattern. In some cases, although the Expasy-Translate Tool software highlights the longest frame beginning with a methionine, comparing the sequence with similar proteins in known databases (NCBI protein database), it was possible to indicate the correct frame. In some cases. the frame is not complete at 3’ or at 5’ ends and comparing it with similar *H. illucens* proteins or with

similar sequences in known protein databases (NCBI protein database), it is possible to confirm that the sequence from the transcriptome is or is not complete and if it includes or not includes the entire mature peptide.

**Contig larve *vs* contig adulti**	**Query cover**	**E value**	**Identity**	**FPKMF female**	**FPKMF female antennae**	**FPKMF male**	**FPKMF male antennae**	**FPKMF larve**
L21691 A41232	100%	7e-104	99.29%	-10,7649	3,69384	-10,7649	5,35387	0,0401
L21691 A30834	100%	2e-79	75.18%	-10,7649	2,61425	-4,24521	3,09515	0,0401
L13368 A30834	100%	1e-104	100.00%	-10,7649	2,61425	-4,24521	3,09515	0,89892
L13368 A41232	100%	3e-79	75.18%	-10,7649	3,69384	-10,7649	5,35387	0,89892
L57 A2226	100%	4e-103	100.00%	4,11987	10,57058	5,53602	12,01216	11,85349
L11107 A11394	100%	2e-105	100.00%	9,55913	-1,7328	2,20579	-0,46969	-0,34034
L31956 A2668	100%	2e-72	64.19%	6,6053	13,19403	7,24019	13,70307	0,31151
L45961 A43028	100%	8e-134	100.00%	-0,19222	2,52798	-4,85699	-2,47856	-0,92375
L441 A7966	99%	7e-74	73.85%	3,47829	0,15025	1,58682	0,00442	9,37253
L441 A12769	100%	2e-69	67.94%	3,86579	0,56347	2,73711	0,17682	9,37253
L441 A1903	99%	3e-50	54.62%	4,65469	7,27189	5,03439	8,84078	9,37253
L758 A1003	100%	6e-81	82.31%	6,68164	2,84356	5,75724	3,43187	11,03303
L758 A1002	100%	1e-81	83.08%	0,93363	-1,38168	4,46701	2,46501	11,03303
L758 A32259	100%	4e-72	74.81	-0,96758	0,82233	-2,64139	2,88503	11,03303
L758 A23172	100%	4e-63	65.19%	0,28088	3,84337	-0,28919	3,35445	11,03303

L758<br>A5932	99%	1e-62	63.85%	6,30632	2,36596	5,80518	3,93491	11,03303
L768<br>A11523	100%	4e-100	98.55%	2,32154	2,35944	-0,07859	6,06551	7,61154
A768<br>A8085	98%	9e-53	50.00%	5,55537	8,18802	3,79547	9,13051	7,61154
L1173<br>A8987	100%	3e-103	99.25%	-0,27302	-10,7649	-2,13043	-4,14432	9,13816
L1844<br>A420	100%	7e-97	97.71%	-2,30704	3,12847	7,92604	3,81125	5,58966
L1844<br>A410	100%	7e-97	100.00%	7,11937	3,25015	7,79462	5,78825	5,58966
L2633<br>A1002	100%	2e-94	96.15%	0,93363	-1,38168	4,46701	2,46501	8,23609
L2633<br>A1003	100%	6e-98	99.23%	6,68164	2,84356	5,75724	3,43187	8,23609
L2633<br>A32259	100%	3e-71	73.28%	-0,96758	0,82233	-2,64139	2,88503	8,23609
L2633<br>A23172	100%	2e-61	62.96%	0,28088	3,84337	-0,28919	3,35445	8,23609
L2633<br>A5932	99%	3e-60	62.31%	6,30632	2,36596	5,80518	3,93491	8,23609
L3948<br>A23172	100%	8e-102	99.26%	0,28088	3,84337	-0,28919	3,35445	9,34447
L3948<br>A32259	100%	3e-63	61.48%	-0,96758	0,82233	-2,64139	2,88503	9,34447
L3948<br>A1002	100%	2e-62	63.70%	0,93363	-1,38168	4,46701	2,46501	9,34447
L3948<br>A1003	100%	4e-61	62.22%	6,68164	2,84356	5,75724	3,43187	9,34447
L3948<br>A5932	99%	2e-59	59.70%	6,30632	2,36596	5,80518	3,93491	9,34447
L3962<br>A23172	100%	2e-86	82.96%	0,28088	3,84337	-0,28919	3,35445	7,72721
L3962<br>A1003	100%	2e-65	68.15%	6,68164	2,84356	5,75724	3,43187	7,72721
L3962	100%	2e-66	68.89%	0,93363	-1,38168	4,46701	2,46501	7,72721

A1002								
L3962 A32259	100%	2e-64	63.70%	-0,96758	0,82233	-2,64139	2,88503	7,72721
L3962 A5932	99%	6e-63	61.94%	6,30632	2,36596	5,80518	3,93491	7,72721
L3982 A2731	100%	3e-101	99.26%	5,60252	3,68816	4,80744	4,02918	6,59036
L4168 A420	100%	1e-88	87.79%	-2,30704	3,12847	7,92604	3,81125	4,44637
L4168 A410	100%	1e-88	87.79%	7,11937	3,25015	7,79462	5,78825	4,44637
L5161 A420	100%	5e-89	88.55%	-2,30704	3,12847	7,92604	3,81125	4,62042
L5161 A410	100%	5e-89	88.55%	7,11937	3,25015	7,79462	5,78825	4,62042
L7404 A23172	100%	3e-85	81.48%	0,28088	3,84337	-0,28919	3,35445	5,17879
L7404 A1003	100%	2e-64	66.67%	6,68164	2,84356	5,75724	3,43187	5,17879
L7404 A1002	100%	2e-65	67.41%	0,93363	-1,38168	4,46701	2,46501	5,17879
L7404 A32259	100%	2e-63	62.96%	-0,96758	0,82233	-2,64139	2,88503	5,17879
L7404 A5932	99%	3e-62	61.19%	6,30632	2,36596	5,80518	3,93491	5,17879
L7621 A23172	100%	8e-79	74.07%	0,28088	3,84337	-0,28919	3,35445	0,3045
L7621 A1002	100%	8e-58	60.00%	0,93363	-1,38168	4,46701	2,46501	0,3045
L7621 A1003	100%	5e-57	59.26%	6,68164	2,84356	5,75724	3,43187	0,3045
L7621 A32259	100%	3e-57	57.78%	-0,96758	0,82233	-2,64139	2,88503	0,3045
L7621 A5932	99%	2e-55	58.21%	6,30632	2,36596	5,80518	3,93491	0,3045
L9011 A8085	100%	7e-105	100.00%	5,55537	8,18802	3,79547	9,13051	5,04794

L9011 A11523	97%	5e-55	51.47%	2,32154	2,35944	-0,07859	6,06551	5,04794
L13738 A3768	100%	9e-101	100.00%	7,33693	5,82076	6,06532	5,78634	0,1514
L14606 A5932	100%	1e-98	96.27%	6,30632	2,36596	5,80518	3,93491	3,0838
L14606 A1003	99%	2e-59	60.77%	6,68164	2,84356	5,75724	3,43187	3,0838
L14606 A32259	97%	4e-64	67.94%	-0,96758	0,82233	-2,64139	2,88503	3,0838
L14606 A1002	97%	5e-61	62.31%	0,93363	-1,38168	4,46701	2,46501	3,0838
L14606 A23172	97%	6e-59	59.70%	0,28088	3,84337	-0,28919	3,35445	3,0838
L1936 A7966	100%	6e-69	64.89%	3,47829	0,15025	1,58682	0,00442	5,2587
L1936 A12769	99%	3e-72	70.00%	3,86579	0,56347	2,73711	0,17682	5,2587
L1936 A1903	99%	2e-46	53.08%	4,65469	7,27189	5,03439	8,84078	5,2587
L13737 A3768	100%	3e-68	69.47%	7,33693	5,82076	6,06532	5,78634	0,66434
L59460 A17957	100%	2e-108	95.45%	-2,81417	-2,34619	-1,02855	-1,27948	0,34166

Table S6: Complete OBP sequences from larvae and adult transcriptome were compared using the Basic Local Alignment Search Tool Protein (BLASTp). Query cover, E-value, Similarity and FPKMF of female body, female antennae, male body and male antennae are reported. Alignment with identity between 97% and 100% and identity greater than 50% are reported.

```
L11107    MKLFLVFWTIFALVSAEWVPRTSDQMYKDQAECFKQLELTEEEQQKVKKEDFPDEPKFRC    60
A11394    MKLFLVFWTIFALVSAEWVPRTSDQMYKDQAECFKQLELTEEEQQKVKKEDFPDEPKFRC    60
          ************************************************************
L11107    YLRCILMGGQIWDDEKGYNPERAYAELLNIDMTADVENLRKCNTQNLHHSDSCTRAFRVV    120
A11394    YLRCILMGGQIWDDEKGYNPERAYAELLNIDMTADVENLRKCNTQNLHHSDSCTRAFRVV    120
          ************************************************************
L11107    KCFANNNYITSIKPKS    136
A11394    KCFANNNYITSIKPKS    136
          ****************

L21691    MNCFASVFVIVAALVAVSQANVNDPKLKSILEQCIGSEKASPADIAALEARSSDLSKEAK    60
A41232    MNCFASVFVIVAALFAVSQANVNDPKLKSILEQCIGSEKASPADIAALEARSSDLSKEAK    60
          **************.*********************************************
L21691    CVISCVMKNYKLLSDDGKVNRDVFMAEAEEMTKGDAGAMKEAGEMFEICSAKTVADPCES    120
A41232    CVISCVMKNYKLLSDDGKVNRDVFMAEAEEMTKGDAGAMKEAGEMFEICSAKTVADPCES    120
          ************************************************************
L21691    AFNFGHCMKTEMTARNIPMDF       141
A41232    AFNFGHCMKTEMTARNIPMDF       141
          *********************

L1844     MKILISLFAIFVLVTADWEPRNREQFIKDRDECFKSENLSEHCIDEIKRRIFPHEPKCYF    60
A410      MKILISLFAIFVLVTADWEPRNREQFIKDRDECFKSENLSEHCIDEIKRRIFPHEPKCYF    60
          ************************************************************
L1844     RCVLMKNSVWDDTSGYDVEKAHKELTHNGLEIAKEDLNKCNSEEMKNKDPCVWANNIIKC    120
A410      RCVLMKNSVWDDTSGYDVEKAYKELTHNGLEIAKEDLNKCNSDEMKNKDPCVWANNIIKC    120
          *********************:********************:*****************
L1844     IWDYLPKKKQE  131
A410      IWDYLPKKKQD  131
          **********:

L2633     MKFFIVLCAIIAMAFAISTEEFQEMREECFKSEKVPEADIEKLKHREYGLDLGHEAKCYI    60
A1002     MKFFIVLCAIIAMASAISTEEFQKMREECFKSEKVPEADIEKLKNREYGLDLGHEAKCYI    60
          ************** ********:********************:***************
L2633     RCLGMKTGNWDDTNGYDVEKIYTDFRTAGLEVTKENLKKCFKSSGDDDKCVWAAKDLKCL    120
A1002     RCLGMKTGNWDDTNGYDVEKIYTDFRTAGLEVTKENLNKCFKSSGDDDKCVWAAKDLKCL    120
          *************************************:**********************
L2633     WTNKYMSRKQ   130
A1002     WTNKYISRKQ   130
          *****:****

L2633     MKFFIVLCAIIAMAFAISTEEFQEMREECFKSEKVPEADIEKLKHREYGLDLGHEAKCYI    60
A1003     MKFFIVLCAIIAMAFAISTEEFQEMREECFKSEKVPEADIEKLKHREYGLDLGHEAKCYI    60
          ************************************************************
L2633     RCLGMKTGNWDDTNGYDVEKIYTDFRTAGLEVTKENLKKCFKSSGDDDKCVWAAKDLKCL    120
A1003     RCLGMKTGNWDDTNGYDVEKIYTDFRTAGLEVTKENLKKCFKSSGDDDKCVWAAKDLKCL    120
          ************************************************************
L2633     WTNKYMSRKQ   130
A1003     WTNKYISRKQ   130
          *****:****

L3948     MKFFIVLCVVIAMAAAQWTPKSKEELLKFREECFKSENVPQAVIEKLNNRQYDEDLGQEA    60
A23172    MKFFIVLCAVIAMAAAQWTPKSKEELLKFREECFKSENVPQAVIEKLNNRQYDEDLGQEA    60
          ********.***************************************************
L3948     RCYIRCIGLKSEIWDDTNGYDLERSYQNLISTGFEVSKENLQKCITPNTDNDDKCTWAAK    120
A23172    RCYIRCIGLKSEIWDDTNGYDLERSYQNLISTGFEVSKENLQKCITPNTDNDDKCTWAAK    120
          ************************************************************
L3948     NLKCLWTNKYVTKKQ      135
A23172    NLKCLWTNKYVTKKQ      135
          ***************

L13368    MNRFASVFVIVAALAALSQADVNDPRLKASLEKCIGSEKASPADVEALKAHSSDLSREAQ    60
A30834    MNRFASVFVIVAALAALSQADVNDPRLKASLEKCIGSEKASPADVEALKAHSSDLSREAQ    60
          ************************************************************
L13368    CVMACVMKEFKLLGDDGKINRDVYMAEAEEMAKGDAGAIKQATEMYDICSAKTVADNCES    120
A30834    CVMACVMKEFKLLGDDGKINRDVYMAEAEEMAKGDAGAIKQATEMYDICSAKTVADNCES    120
          ************************************************************
L13368    ANNFGQCIKNEMIARNIPLDM       141
A30834    ANNFGQCIKNEMIARNIPLDM       141
          *********************

L768      MKFFVASCVLLAVASAQWAPKTGKEVAQIREDCVKEENVPESLAEGLKKFEYPDEEPIRC    60
A11523    MKFFVASCVLLAVASAQWAPKTGKEVAQIREDCVKEENVPESLAEGLKKFEYPDEEPIRC    60
          ************************************************************
L768      YVKCVSAKLGVWNDETGFDADRVADQVKQDRNKDDIKAEVEKCIDKNEQNSDKCTWAXXN    120
A11523    YVKCVSAKLGVWNDETGFDADRVADQVKQDRNKDDIKAEVEKCIDKNEQNSDKCTWAYRN    120
          *********************************************************  *
L768      LKCVMDKKLLQVESLIAN   138
A11523    LKCVMDKKLLQVESLIAN   138
          ******************

L45961    MPLPTTYFRGCLFLFLVAVFAEDHCSKPPPKFAPRECCEPVKWSESNVEDKCAGAPSAVC    60
A43028    MPLPTTYFRGCLFLFLVAVFAEDHCSKPPPKFAPRECCEPVKWSESNVEDKCAGAPSAVC    60
          ************************************************************
L45961    FTECIFNSTDIIQNDGPNATNIRTFFEIELKADPDYIPIAINNFEMCYSLVKRRMEDKDH    120
A43028    FTECIFNSTDIIQNDGPNATNIRTFFEIELKADPDYIPIAINNFEMCYSLVKRRMEDKDH    120
          ************************************************************
L45961    QIDGCDTFPALIMECTIAQMFVDCPSEKWKSSEACELARKYYKRCPDRHFTTNA     174
A43028    QIDGCDTFPALIMECTIAQMFVDCPSEKWKSSEACELARKYYKRCPDRHFTTNA     174
          ******************************************************

L2633     MKFFIVLCAIIAMAFAISTEEFQEMREECFKSEKVPEADIEKLKHREYGLDLGHEAKCYI    60
A1003     MKFFIVLCAIIAMAFAISTEEFQEMREECFKSEKVPEADIEKLKHREYGLDLGHEAKCYI    60
          ************************************************************
L2633     RCLGMKTGNWDDTNGYDVEKIYTDFRTAGLEVTKENLKKCFKSSGDDDKCVWAAKDLKCL    120
A1003     RCLGMKTGNWDDTNGYDVEKIYTDFRTAGLEVTKENLKKCFKSSGDDDKCVWAAKDLKCL    120
          ************************************************************
L2633     WTNKYMSRKQ   130
A1003     WTNKYISRKQ   130
          *****:****

L1173     MKSLIIFCAIFAVVFANWSTPTKEQFKQHRDDCLKEGNVPEETANKIRKEQYPNDRDTYC    60
A8987     MKSLIIFCAIFAVVFANWSTPTKEQFKQHRDDCLKEGNVPEETANKIRKEQYPNDRDTYC    60
          ************************************************************
L1173     YIRCVGSKSGIWNDRKGYDIDRSLQVFEANGYEVTRENLERCFAPLPGADTCTWAGVNMR    120
A8987     YIRCVGSKSGIWNDRKGYDIDRALQVFEANGYEVTRENLERCFAPLPGADTCTWAGVNMR    120
          **********************:*************************************
L1173     CLRDNKYVTKKASA       134
A8987     CLRDNKYVTKKASA       134
          **************

L57       MKVLVLLAVIGVAAATYTIKTHDDLIKTRGLCVKELNVPDNYVEKFKKWDFQDDETTRCY    60
A2226     MKVLVLLAVIGVAAATYTIKTHDDLIKTRGLCVKELNVPDNYVEKFKKWDFQDDETTRCY    60
          ************************************************************
L57       IKCVLNKMELFDTANGFNVENLVEQLGQNKDKTEVRTEVTKCSDKNEQKSDDCTWAYRGF    120
A2226     IKCVLNKMELFDTANGFNVENLVEQLGQNKDKTEVRTEVTKCSDKNEQKSDDCTWAYRGF    120
          ************************************************************
L57       KCFLSKHLQLVQSSVKS    137
A2226     KCFLSKHLQLVQSSVKS    137
          *****************

L3982     MKCSLICFLLVFAIASGSDWEPKGNEELVSIRDECFKLENVSEESAAKILKNEYPDEPSV    60
A2731     MKCSLVCFLLVFAIASGSDWEPKGNEELVSIRDECFKLENVSEESAAKILKNEYPDEPSV    60
          *****:******************************************************
L3982     HCYVRCTSAKVGTWDDEAGPDIDRTLRQIQKSGNTITREELVRCKPEKQENKCLWAYKGL    120
A2731     HCYVRCTSAKVGTWDDEAGPDIDRTLRQIQKSGNTITREELVRCKPEKQENKCLWAYKGL    120
          ************************************************************
L3982     MCILKSEGIKDDLIK      135
A2731     MCILKSEGIKDDLIK      135
          ***************

L9011     MKLFVVLAAIVAFASAEWATRSPAELAQSRLSCIQELSVPANLVEQIKKFQYPDEEIVRC    60
A8085     MKLFVVLAAIVAFASAEWATRSPAELAQSRLSCIQELSVPANLVEQIKKFQYPDEEIVRC    60
          ************************************************************
L9011     YIRCTSEKIGIWSDDSGFITDRVIEQLAGNRDKDAFRADVLKCIDSNEQKSDKCTWAYRN    120
A8085     YIRCTSEKIGIWSDDSGFITDRVIEQLAGNRDKDAFRADVLKCIDSNEQKSDKCTWAYRN    120
          ************************************************************
L9011     FNCFIKNNLILVQSQINASS 140
A8085     FNCFIKNNLILVQSQINASS 140
          ********************

L13738    MKLLIFLFAIFVLVAADWKPRSREQYTKDGDECFKSENISEDGIHEIRRHVFTDDSKCFF    60
A3768     MKLLIFLFAIFVLVAADWKPRSREQYTKDGDECFKSENISEDGIHEIRRHVFTDDSKCFF    60
          ************************************************************
L13738    RCVLMKNHVWDDTTGYNVERVYKEVTHIGLKASKDGLTQCNSDDKKDKDPCQWVNNIVRC    120
A3768     RCVLMKNHVWDDTTGYNVERVYKEVTHIGLKASKDGLTQCNSDDKKDKDPCQWVNNIVRC    120
          ************************************************************
L13738    VFEHNYIEPNY  131
A3768     VFEHNYIEPNY  131
          ***********
```

Figure S3. Sequence alignments performed by Clustal Omega (https://www.ebi.ac.uk/Tools/msa/clustalo), between the sequences of common OBPs in larval and adult transcriptomes.

VOC	Source and/or process	References
- acetic acid,1-methylethyl ester<br>- 2-hexen-1-ol, (E)<br>- acetophenone<br>- propanoic acid, 1-methylethyl ester	Thermal decomposition<br>Fermentation and/or oxidative processes	[203-205]
- 2-methyl-butanal<br>- butanoic acid, propyl ester	Milk and meat spoilage (lipid degradation)	[206-209]
- 1-hexanol<br>- 3-octanone,<br>- 1-butanol, 3-methyl acetate,<br>- delta limonene<br>- 3-methyl butanal	Fish and meat spoilage (microbial contamination or unsuitable storage)	[28, 207, 210-212]
- propanoic acid, ethyl ester<br>- 2-hexanone<br>- acetic acid, butyl ester<br>-2-heptanone<br>- styrene<br>- hexanoic acid, ethyl ester<br>- butanoic acid, 2-methyl, hexyl ester<br>- acetic acid, 2-methylpropyl ester<br>- alpha farnesene<br>- 2-pentanone<br>- 1-butanol	Fungi and bacteria (food microbial contaminations)	[26, 213-217]
- butanoic acid, 1-methylethyl ester	Faecal contaminations	[218]
- n-propyl acetate<br>-3-methyl-1-butanol	Vegetable spoilage	[219]

Table S7. Selected VOCs of interest, index of specific phases of organic degradation, in different food matrices. VOC name and source and/or process generating these VOCs were reported.

References:

105. Pontes, M.; Pereira, J.; Câmara, J.S. Dynamic headspace solid-phase microextraction combined with one-dimensional gas chromatography-mass spectrometry as a powerful tool to differentiate banana cultivars based on their volatile metabolite profile. *Food Chem.* **2012**, *134*, 2509–2520.

106. Satora, P.; Cioch, M.; Tarko, T.; Wołkowicz, J. Killer strains of *Saccharomyces*: Application for apple wine production. *J. Inst. Brew.* **2016**, *122*, 412–421.

107. Gao, P.; Martin, J. Volatile metabolites produced by three strains of *Stachybotrys chartarum* cultivated on rice and gypsum board. *J. Occup. Environ. Hyg.* **2002**, *17*, 430–436.

108. Espino-Díaz, M.; Sepúlveda, D.R.; González-Aguilar, G.; Olivas, G.I. Biochemistry of Apple Aroma: A Review. *Food Technol. Biotechnol.* **2016**, *54*, 375–397.

109. Calamai, L.; Villanelli, F.; Bartolucci, G.; Pieraccini, G.; Moneti, G. Sample Preparation for Direct MS Analysis of Food. In *Comprehensive Sampling and Sample Preparation*; Pawliszyn, J., Ed.; Academic Press: Amsterdam, The Netherlands, 2012; pp. 535–557.

110. Facundo, H.V.V.; Garruti, D.S.; Cordenunsi, B.R.; Lajolo, F.M. Isolation of volatiles compounds in banana by HS–SPME: Optimization for the whole fruit and pulp. *Int. J. Biosci. Biochem. Bioinform* **2013**, *3*, 110–115.

111. Pino, J.A.; Febles, Y. Odour-active compounds in banana fruit cv. Giant Cavendish. *Food Chem.* **2013**, *141*, 795–801.

112. Boonsupa, W.; Chumchuere, S.; Chaovarat, M. Evolution of Volatile Compounds Generated During One-Stage and Two-Stage Fermentation and Aging Processes of Banana Vinegars Using HS-SPME-GC-MS. *Indian J. Biotechnol.* **2017**, *13*, 147.

113. Tian, J.; Yu, J.; Chen, X.; Zhang, W. Determination and quantitative analysis of acetoin in beer with headspace sampling-gas chromatography. *Food Chem.* **2009**, *112*, 1079–1083.

114. Liang, S.; Wan, C. Carboxylic acid production from brewer's spent grain via mixed culture fermentation. *Bioresour. Technol.* **2015**, *182*, 179–183.

115. Macku, C.; Jennings, G. Production of volatiles by ripening bananas. *J. Agric. Food Chem.* **1987**, *35*, 845–848.

116. Harb, J.; Streif, J.; Bangerth, K.F. Aroma volatiles of apples as influenced by ripening and storage procedures. *Acta Hortic.* **2008**, *796*, 93–103.

117. Dong, L.; Hou, Y.; Li, F.; Piao, Y.; Zhang, X.; Zhang, X.; Li, C.; Zhao, C. Characterization of volatile aroma compounds in different brewing barley cultivars. *J. Sci. Food Agric.* **2015**, *95*, 915–921.

118. Overton, S.F.; Manura, J.J. Note 17- Identification of Volatile Organics in Wines Over Time. Available online: https://www.sisweb.com/referenc/applnote/app-17.htm (accessed in February 20, 2017.

119. Krzymien, M.; Day, M.; Shaw, K.; Zaremba, L. An Investigation of Odors and Volatile Organic Compounds Released during Composting. *J. Air Waste Manag.* **1999**, *49*, 804–813.

120. Verstrepen, K.J.; Derdelinckx, G.; Dufour, J.P.; Winderickx, J.; Thevelein, J.M.; Pretorius, I.S.; Delvaux, F.R. Flavor-active esters: Adding fruitiness to beer. *J. Biosci. Bioeng.* **2003**, *96*, 110–118.

121. Kopsahelis, N.; Kanellaki, M.; Bekatorou, A. Low temperature brewing using cells immobilized on Brewer's spent grains. *Food Chem.* **2007**, *104*, 480–488.

122. Wang, J.; Chen, Q.; Hu, K.; Zeng, L.; Pan, Y.; Huang, H. Change in aromatic components of banana during the preparation process of juice and microcapsule powder. *J. Food Sci. Technol.* **2011**, *46*, 1398–1405.

123. FooDB. Available online: https://foodb.ca/compounds/FDB008079 (accessed in February 20, 2017).

124. Madrera, R.R.; Valles, B.S. Determination of Volatile Compounds in Apple Pomace by Stir Bar Sorptive Extraction and Gas Chromatography-Mass Spectrometry (SBSE-GC-MS). *J. Food Sci.* **2011**, *76*, C1326–C1334.

125. Cellini, A.; Biondi, E.; Blasioli, S.; Rocchi, L.; Farneti, B.; Braschi, I.; Savioli, S.; Rodriguez-Estrada, M.; Biasioli, F.; Spinelli, F. Early detection of bacterial diseases in apple plants by analysis of volatile organic compounds profiles and use of electronic nose. *Ann. Appl. Biol.* **2016**, *168*, 409–420.

126. Toledo, J.; Malo, E.A.; Cruz-López, L.; Rojas, J.C. Field evaluation of potential fruit-derived lures for *Anastrepha obliqua* (Diptera: *Tephritidae*). *J. Econ. Entomol.* **2009**, *102*, 2072–2077.

127. Beaulieu, J.C.; Grimm, C.C Identification of Volatile Compounds in Cantaloupe at Various Developmental Stages Using Solid Phase Microextraction. *J. Agric. Food Chem.* **2001**, *49*, 1345–1352.

128. Jordán, M.J.; Tandon, K.; Shaw, P.E.; Goodner, K.L. Aromatic profile of aqueous banana essence and banana fruit by gas chromatography-mass spectrometry (GC-MS) and gas chromatography-olfactometry (GC-O). *J. Agric. Food Chem.* **2001**, *49*, 4813–4817.

129. Api, A.M.; Belsito, D.; Botelho, D.; Bruze, M.; Burton, G.A.; Buschmann, J.; Dagli, M.L.; Date, M.; Dekant, W.; Deodhar, C.; *et al.* RIFM fragrance ingredient safety assessment, ethyl 2-methylbutyrate, CAS Registry Number 7452-79-1. *Food Chem. Toxicol.* **2018**, *122*, 738–S746.
130. Ferreira, L.; Perestrelo, R.; Caldeira, M.; Câmara, J.S. Characterization of volatile substances in apples from Rosaceae family by headspace solid-phase microextraction followed by GC-qMS. *J. Sep. Sci.* **2009**, *32*, 1875–1888.
131. Kim, E.; Chang, Y.H.; Ko, J.Y.; Jeong, Y. Physicochemical and Microbial Properties of the Korean Traditional Rice Wine, Makgeolli, Supplemented with Banana during Fermentation. *Prev. Nutr. Food Sci.* **2013**, *18*, 203–209.
132. Palade, L.M.; Duta, D.; Popescu, C.; Croitoru, C.; Popa, M.E. Differentiation of three grape varieties by means of sensory analysis and characterization of the volatile compounds profile of their musts. *Rom. Biotechnol. Lett.* **2016**, 22, 12005.
133. Capobiango, M.; Mastello, R.B.; Chin, S.-T.; Oliveira, E.d.S.; de Lourdes Cardeal, L.; Marriott, P.J. Identification of aroma-active volatiles in banana Terra spirit using multidimensional gas chromatography with simultaneous mass spectrometry and olfactometry detection. *J. Chromatogr. A* **2015**, *3*, 1388, 227–235.
134. Kleinova, J.; Klejdus, B. Determination of Volatiles in Beer using Solid-Phase Microextraction in Combination with Gas Chromatography/Mass Spectrometry. *Czech J. Food Sci.* **2014**, *32*, 241–248.
135. Zhu, X.; Li, Q.; Li, J.; Luo, J.; Chen, W.; Li, X. Comparative Study of Volatile Compounds in the Fruit of Two Banana Cultivars at Different Ripening Stages. *Molecules* **2018**, *23*, 2456.
136. Fuhrmann, E.; Grosch, W. Character impact odorants of the apple cultivars Elstar and Cox Orange. *Nahrung* **2002**, *46*, 187–193.
137. Yauk, Y.-K.; Souleyre, E.J.F.; Matich, A.J.; Chen, X.; Wang, M.Y.; Plunkett, B.; Dare, A.P.; Espley, R.V.; Tomes, S.; Chagné, D.; *et al.* Alcohol acyl transferase 1 links two distinct volatile pathways that produce esters and phenylpropenes in apple fruit. *Plant J.* **2017**, *91*, 2292–2305.
138. Selvaraj, G.; Ramanathan, T.; Kaliamurthi, S. Characterization of Volatile Compounds from Bitter Apple (*Citrullus colocynthis*) Using GC-MS. *Int. J. Anal. Chem*. **2011**, *2*, 108–110.
139. Miranda, E.J.F.; Nogueira, R.I.; Pontes, S.M.; Rezende, C.M. Odour-active compounds of banana passa identified by aroma extract dilution analysis. *Flavour Frag. J.* **2001**, *16*, 281–285.
140. Chen, Y.; Zhou, Z.; Xu, K.; Zhang, H.; Thornton, M.; Sun, L.; Wang, Z.; Xu, X.; Dong, L. Comprehensive evaluation of malt volatile compounds contaminated by *Fusarium graminearum* during malting. *J. Inst. Brew*. **2017**, *123*, 480–487.
141. Steele, D.H.; Midwest, R.I.; Thornburg, M.J.; Stanley, J.S.; Miller, R.R.; Brooke, R.; Cushman, J.R.; Cruzan, G. Determination of styrene in selected foods. *J. Agric. Food Chem.* **1994**, *42*, 8, 1661–1665.
142. Langos, D.; Gastl, M.; Granvogl, M. Reduction of toxicologically relevant styrene in wheat beer using specially produced wheat and barley malts. *Eur. Food Res. Technol.* **2017**, *243*, 1711–1718.
143. Gou, W.; Zhang, L.; Chen, F.; Cui, Z.; Zhao, Y.; Zheng, P.; Tian, L.; Zhang, L. Foliar application of amino acids modulates aroma components of 'fuji' apple (*Malus Domestica* L.) *Pak. J. Bot*. **2015**, *47*, 6, 2257–2262.
144. FooDB. Available online: https://foodb.ca/compounds/FDB001358 (accessed in February 20, 2017).
145. López, M.; Lavilla, M.; Riba, M.; Vendrell, M. Comparison of Volatile Compounds in Two Seasons in Apples: Golden Delicious and Granny Smith. *J. Food Qual.* **1998**, *21*, 155–166.
146. Kaminski, E.; Stawicki, S.; Wasowicz, E. Volatile Flavor Compounds Produced by Molds of *Aspergillus*, *Penicillium*, and *Fungi imperfecti*. *Appl. Microbiol.* **1974**, *27*, 1001–1004.
147. Mehinagic, E.; Royer, G.; Symoneaux, R.; Jourjon, F.; Prost, C. Characterization of odor-active volatiles in apples: Influence of cultivars and maturity stage. *J. Agric. Food Chem.* **2006**, *54*, 2678–2687.

148. Wang, J.; Li, Y.; Chen, R.; Bao, J.; Yang, G. Comparison of volatiles of banana powder dehydrated by vacuum belt drying, freeze-drying and air-drying. *Food Chem.* **2007**, *104*, 1516–1521.
149. Fărcaş, A.; Socaci, S.; Francisc, D.; Tofana, M.; Mudura, E.; Diaconeasa, Z. Volatile profile, fatty acids composition and total phenolics content of brewers' spent grain by-product with potential use in the development of new functional foods. *J. Cereal Sci.* **2015**, *64*, 34–42.
150. Wongs-Aree, C.; Noichinda, S. Sugar apple (*Annona squamosa* L.) and atemoya (A. cherimola Mill. × A. squamosa L.). In *Postharvest Biology and Technology of Tropical and Subtropical Fruits: Mangosteen to White Sapote*; Yahia, E.M., Ed.; Woodhead Publishing Limited: Sawston, UK, 2011, pp. 399–426.

151. Truta, D.; Tofana, M.; Socaci, S. The Evolution of The Volatile Compounds by Aging, In Apple Vinegar Flavored with *Rosmarinus Officinalis* L., Using A GC-MS Technique. *J. Agroaliment. Process. Technol.* **2010**, *16*, 155.
152. Pino, J.A.; Marbot, R.; Rosado, A.; Vázquez, C. Volatile constituents of Malay rose apple [*Syzygium malaccense* (L.) Merr. and Perry]. *Flavour Frag. J.* **2004**, *19*, 32–35.
153. Shiota, H. New esteric components in the volatiles of banana fruit (*Musa sapientum* L.). J. *Agric. Food Chem.* **1993**, *41*, 2056–2062.
154. Fărcaș, A.C.; Socaci, S.A.; Tofană, M.; Mureșan, C.; Mudura, E.; Salanță, L.; Scrob, S. Nutritional properties and volatile profile of brewer's spent grain supplemented bread. *Rom. Biotechnol. Lett*. **2014**, *19*, 9075–9714.
155. López, M.L.; Villatoro, C.; Fuentes, T.; Graell, J.; Lara, I.; Echeverría, G. Volatile compounds, quality parameters and consumer acceptance of 'Pink Lady®' apples stored in different conditions. *Postharvest Biol. Technol.* **2007**, *43*, 55–66.
156. Wei, A.; Mura, K.; Shibamoto, T. Antioxidative Activity of Volatile Chemicals Extracted from Beer. *J. Agric. Food Chem.* **2001**, *9*, 4097–4101.
157. Lara, I.; Graell, J.; López, M.L.; Echeverría, G. Multivariate analysis of modifications in biosynthesis of volatile compounds after CA storage of 'Fuji' apples, *Postharvest Biol. Technol.* **2006**, *39*, 19–28.
158. Rowan, D.D.; Hunt, M.B.; Dimouro, A.; Alspach, P.A.; Weskett, R.; Volz, R.K.; Gardiner, S.E.; Chagné, D. Profiling fruit volatiles in the progeny of a 'Royal Gala' x 'Granny Smith' pple (*Malus* x *domestica*) cross. *J. Agric. Food Chem.* **2009**, *57*, 7953–7961.
159. Fleming-Jones, M.; Smith, R. Volatile Organic Compounds in Foods: A Five Year Study. *J. Agric. Food Chem.* **2004**, *51*, 8120–8127.
160. Chen, L.Y.; Wu, C.C.; Chou, T.I.; Chiu, S.W.; Tang, K.T. Development of a Dual MOS Electronic Nose/Camera System for Improving Fruit Ripeness Classification. *Sensors* **2018**, *18*, 3256.
161. Sun, J. D-Limonene: Safety and clinical applications. *Sci. Rev. Altern. Med*. **2007**, *12*, 259–264.
162. Fein, B.L.; Reissig, W.H.; Roelofs, W.L. Identification of apple volatiles attractive to the apple maggot, *Rhagoletis pomonella*. *J. Chem. Ecol.* **1982**, *8*, 1473–1487.
163. Lasekan, O.; Khatib, A.; Juhari, H.; Patiram, P.; Lasekan, S. Headspace solid-phase microextraction gas chromatography-mass spectrometry determination of volatile compounds in different varieties of African star apple fruit (*Chrysophillum albidum*). *Food Chem.* **2013**, *141*, 2089–2097.
164. Api, A.M.; Belsito, D.; Botelho, D.; Bruze, M.; Burton, G.A.; Buschmann, J.; Dagli, M.L.; Date, M.; Dekant, W.; Deodhar, C.; *et al.* RIFM fragrance ingredient safety assessment, acetophenone, CAS Registry Number 98-86-2. *Food Chem. Toxicol.* **2018**, *118*, 162–169.
165. Hern, A.; Dorn, S. A female-specific attractant for the codling moth, Cydia pomonella, from apple fruit volatiles. *Naturwissenschaften* **2004**, *91*, 77–80.
166. Balázs, A.; Tóth, M.; Blazics, B.; Héthelyi, É.; Szarka, S.; Ficsor, E.; Ficzek, G.; Lemberkovics, É.; Blázovics, A. Investigation of dietary important components in selected red fleshed apples by GC-MS and LC-MS. *Fitoterapia* **2012**, *83*, 1356–1363.
167. Mir, N.A.; Beaudry, R. Effect of superficial scald suppression by diphenylamine application on volatile evolution by stored Cortland apple fruit. *J. Agric. Food Chem.* **1999**, *47*, 7–11.
168. Matich, A.; Rowan, D. Pathway analysis of branched-chain ester biosynthesis in apple using deuterium labeling and enantioselective gas chromatography-mass spectrometry. *J. Agric. Food Chem*. **2007**, *55*, 2727–2735.

169. Qin, L.; Wei, Q.-P.; Kang, W.-H.; Zhang, Q.; Sun, J.; Liu, S.-Z. Comparison of Volatile Compounds in 'Fuji' Apples in the Different Regions in China. *Food Sci. Technol.* **2017**, *23*, 79–89.
170. Zhang, C.; Chen, X.; Song, H.; Liang, Y.; Zhao, C.; Li, H. Volatile Compound Profiles of *Malus baccata* and *Malus prunifolia* Wild Apple Fruit. *J. Am. Soc. Hortic. Sci.* **2017**, *142*, 126–134.
171. Maga, J.A.; Katz, I. Simple phenol and phenolic compounds in food flavour *Crit. Rev. Food Sci. Nutr.* **1978**, *10*, 323–372.
172. Chan, S.T.; Yao, M.W.Y.; Wong, Y.C.; Wong, T.; Mok, C.S.; Sin, D.W.M. Evaluation of chemical indicators for monitoring freshness of food and determination of volatile amines in fish by headspace solid-phase microextraction and gas chromatography-mass spectrometry. *Eur. Food Res. Technol.* **2006**, *224*, 67–74.
173. Caldeira, M.; Rodrigues, F.; Perestrelo, R.; Marques, J.C.; Câmara, J.S. Comparison of two extraction methods for evaluation of volatile constituents patterns in commercial whiskeys. Elucidation of the main odour-active compounds. *Talanta* **2007**, *74*, 78–90.
174. Polychroniadou, E.; Kanellaki, M.; Iconomopoulou, M.; Koutinas, A.A.; Marchant, R.; Banat, I.M. Grape and apple wines volatile fermentation products and possible relation to spoilage. *Bioresour. Technol.* **2003**, *87*, 337–339.
175. Cheng, H. Volatile flavor compounds in yogurt: A review. *Crit. Rev. Food Sci. Nutr*. **2010**, *50*, 938–950.

176. Aprea, E.; Biasioli, F.; Gasperi, F. Volatile compounds of raspberry fruit: From analytical methods to biological role and sensory impact. *Molecules* **2015**, *20*, 2445–2474.
177. Fukami, K.; Ishiyama, S.; Yaguramaki, H.; Masuzawa, T.; Nabeta, Y.; Endo, K.; Shimoda, M. Identification of distinctive volatile compounds in fish sauce. *J. Agric. Food Chem.* **2002**, *50*, 5412–5416.
178. Steingass, C.B.; Dell, C.; Lieb, V.; Mayer-Ullmann, B.; Czerny, M.; Carle, R. Assignment of distinctive volatiles, descriptive sensory analysis and consumer preference of differently ripened and post-harvest handled pineapple (*Ananas comosus* [L.] Merr.) fruits. *Eur. Food Res. Technol.* **2016**, *242*, 33–43.
179. Dudley, R. Ethanol, Fruit Ripening, and the Historical Origins of Human Alcoholism in Primate Frugivory. *Integr. Comp. Biol.* **2004**, *44*, 315–323.
180. Yan, J.W.; Ban, Z.J.; Lu, H.Y.; Li, D.; Poverenov, E.; Luo, Z.S.; Li, L. The aroma volatile repertoire in strawberry fruit: A review. *J. Sci. Food Agric.* **2018**, *98*, 4395–4402.
181. El Had, M.A.; Zhang, F.J.; Wu, F.F.; Zhou, C.H.; Tao, J. Advances in fruit aroma volatile research. *Molecules* **2013**, *18*, 8200–8229.
182. Bruna, J.M.; Hierro, E.M.; de la Hoz, L.; Mottram, D.S.; Fernández, M.; Ordóñez, J.A. The contribution of *Penicillium aurantiogriseum* to the volatile composition and sensory quality of dry fermented sausages. *Meat Sci.* **2001**, *59*, 97–107.
183. Shahidi, F.; Pegg, R.B. Hexanal as An Indicator of Meat Flavor Deterioration. *J. Food Lipids* **1994**, *1*, 177–186.
184. Petersen, M.A.; Poll, L.; Larsen, L.M. Changes in flavor-affecting aroma compounds during potato storage are not associated with lipoxygenase activity. *Am. Potato J.* **2003**, *80*, 397–402.
185. Jetti, R.R.; Yang, E.; Kurnianta, A.; Finn, C.; Qian, M.C. Quantification of selected aroma-active compounds in strawberries by headspace solid-phase microextraction gas chromatography and correlation with sensory descriptive analysis. *J. Food Sci.* **2007**, *72*, 487–496.
186. Serradilla, M.; Joaquín, M.; Hernández, A.; López Corrales, M.; Ruiz-Moyano Seco de Herrera, S.; de Guía Córdoba, M.; Martín, A. Composition of the Cherry (*Prunus avium* L. and *Prunus cerasus* L.; Rosaceae). In *Nutritional Composition of Fruit Cultivars*; Simmonds, M.S.J., Preedy, V.R., Eds.; Academic Press: Amsterdam, The Netherlands, 2016, pp. 127–147.
187. Parlapani, F.F.; Mallouchos, A.; Serkos, A.; Ioannis, H.; Boziaris, S. Volatile organic compounds of microbial and non-microbial origin produced on model fish substrate un-inoculated and inoculated with gilt-head sea bream spoilage bacteria. *LWT* **2017**, *78*, 54–62.
188. Phan, N.-T.; Kim, K.-H.; Jeon, E.-C.; Kim, U.-H.; Sohn, J.R.; Pandey, S.K. Analysis of volatile organic compounds released during food decaying processes. *Environ. Monit. Assess.* **2012**, *184*, 1683–1692.
189. Wang, Y.; Yang, C.; Li, S.; Yang, L.; Wang, Y.; Zhao, J.; Jiang, Q. Volatile characteristics of 50 peaches and nectarines evaluated by HP–SPME with GC–MS. *Food Chem.* **2009**, *116*, 356–364.

190. Frank, D.C.; Owen, C.M.; Patterson, J. Solid phase microextraction (SPME) combined with gas-chromatography and olfactometry-mass spectrometry for characterization of cheese aroma compounds LWT. *J. Food Sci. Tec.* **2004**, *37*, 139–154.
191. Nogueira, M.C.L.; Lubachevsky, G.; Rankin, S.A. A study of the volatile composition of Minas cheese. *LWT* **2005**, *38*, 555–563.
192. Cho, S.J.; Kim, A.K.; Kwak, J.E.; Kim, J.Y.; Kim, S.J.; Kum, J.Y.; Kim, I.Y.; Kim, J.H.; Chae, Y.Z. Monitoring of 1-hexene and 1-octene in Hygienic Polyethylene-based Packaging. *J. Food Hyg. Saf.* **2011**, *26*, 383–387.
193. Buffo, R.; Cardelli-Freire, C. Coffee flavour: An overview. *Flavour Frag. J.* **2004**, *19*, 99–104.
194. Park, E.R.; Lee, H.J.; Kim, K. Volatile Flavor Components in *Bogyojosaeng* and *Suhong* Cultivars of Strawberry (*Fragaria ananassa* Duch.) *J. Food Sci. Nut.* **2000**, *5*, 119–125.
195. Zhou, A.; McFeeters, R.F. Volatile Compounds in Cucumbers Fermented in Low-Salt Conditions. *J. Agric. Food Chem.* **1998**, *46*, 2117-2122.
196. Lasekan, O.; Azeez, S. Chemo-preventive Activities of Common Vegetables' Volatile Organic Compounds (VOCs). *Pharm. Anal. Acta* **2014**, *2*, 7.
197. Engel, E.; Baty, C.; le Corre, D.; Souchon, I.; Martin, N. Flavor-Active Compounds Potentially Implicated in Cooked Cauliflower Acceptance. *J. Agric. Food Chem.* **2002**, *50*, 6459–6467.
198. Goulet, C.; Mageroy, M.H.; Lam, N.B.; Floystad, A.; Tieman, D.M.; Klee, H.J. Role of an esterase in flavor volatile variation within the tomato clade. *Proc. Natl. Acad. Sci. USA* **2012**, *109*, 19009–19014.
199. Kiritsakis, A.K. Flavor Components of Olive Oil—A Review. *J. Am. Oil Chem. Soc.* **1998**, *75*, 673–681.
200. Ulrich, D.; Hoberg, E.; Rapp, A.; Kecke, S. Analysis of strawberry flavour: Discrimination of aroma types by quantification of volatile compounds. *Zeitschrift für Lebensmitteluntersuchung und-Forschung A* **1997**, *205*, 218– 223.

201. Rosillo, L.; Salinas, M.R.; Garijo, J.; Alonso, G.L. Short communication Study of volatiles in grapes by dynamic headspace analysis Application to the differentiation of some *Vitis vinifera* varieties. *J. Chromatogr. A* **1999**, *847*, 155–159.
202. Pino, J.A. Odour-active compounds in pineapple (*Ananas comosus* [L.] Merril cv. Red Spanish). *Int. J. Food Sci.* **2013**, *48*, 564–570.
203. Akbar Ali, M.; Violi, A. Reaction pathways for the thermal decomposition of methyl butanoate. *J. Org. Chem.* **2013**, *78*, 5898–5908.
204. Cheng, Y.; Xu, Q.; Liu, J.; Zhao, C.; Xue, F.; Zhao, Y. Decomposition of five phenolic compounds in high temperature water. J *Brazil Chem. Soc.* **2014**, *25*, 2102–2107.
205. Papuc, C.; Goran, G.V.; Predescu, C.N.; Nicorescu, V. Mechanisms of oxidative processes in meat and toxicity induced by postprandial degradation products: A review. *Compr. Rev. Food Sci. Food Saf.* **2017**, *16*, 96–123.
206. Decimo, M.; Cabeza, M.C.; Ordóñez, J.A.; De Noni, I.; Brasca, M. Volatile organic compounds associated with milk spoilage by psychrotrophic bacteria. *Int. J. Dairy Technol.* **2018**, *71*, 593–600.
207. Zareian, M.; Böhner, N.; Loos, H.M.; Silcock, P.; Bremer, P.; Beauchamp, J. Evaluation of volatile organic compound release in modified atmosphere-packaged minced raw pork in relation to shelf-life. *Food Packag. Shelf Life* **2018**, *18*, 51–61.
208. Illikoud, N.; Rossero, A.; Chauvet, R.; Courcoux, P.; Pilet, M.-F.; Charrier, T.; Jaffrès, E.; Zagorec, M. Genotypic and phenotypic characterization of the food spoilage bacterium *Brochothrix thermosphacta*. *Food Microb.* **2019**, *81*, 22–31.
209. Ziyaina, M.; Rasco, B.; Coffey, T.; Ünlü, G.; Sablani, S.S. Colorimetric detection of volatile organic compounds for shelf-life monitoring of milk. *Food Control*. **2019**, *100*, 220–226.
210. Bennett, J.W.; Inamdar, A.A. Are some fungal volatile organic compounds (VOCs) mycotoxins? *Toxins* **2015**, *7*, 3785–3804.
211. Comi, G. Spoilage of Meat and Fish. In *The Microbiological Quality of Food: Foodborne Spoilers*; Bevilacqua, A., Corbo, M.R., Sinigaglia, M., Eds.; Woodhead Publishing: Sawston, UK, 2016, pp. 179–210.
212. Pennerman, K.K.; Al-Maliki, H.S.; Lee, S.; Bennett, J.W. Fungal volatile organic compounds (VOCs) and the genus *Aspergillus.* In *New and Future Developments in Microbial Biotechnology and Bioengineering*; Gupta, V.K., Ed.; Elsevier: Amsterdam, The Netherlands, 2016; pp. 95–115.

213. Krings, U.; Hardebusch, B.; Albert, D.; Berger, R.G.; Maróstica, M.; Pastore, G.M. Odor-active alcohols from the fungal transformation of α-farnesene. *J. Agric. Food Chem* **2006**, *54*, 9079–9084.
214. Pinches, S.E.; Apps, P. Production in food of 1, 3-pentadiene and styrene by *Trichoderma* species. *Int. J. Food Microbiol*. **2007**, *116*, 182–185.
215. Karlshøj, K.; Nielsen, P.V.; Larsen, T.O. Fungal Volatiles: Biomarkers of Good and Bad Food Quality. In *Food Mycology: A Multifaceted Approach to Fungi and Food*; Dijksterhuis, J., Samson, R.A., Eds.; CRC Press: Boca Raton, FL, USA, 2007; pp. 279–302.
216. Wang, Y.; Li, Y.; Yang, J.; Ruan, J.; Sun, C. Microbial volatile organic compounds and their application in microorganism identification in foodstuff. *TrAC Trends Analyt. Chem.* **2016**, *8*, 1–16.
217. da Silva, N.C.; Nascimento, C.F.; Nascimento, F.A.; de Resende, F.D.; Daniel, J.L.P.; Siqueira, G.R. Fermentation and aerobic stability of rehydrated corn grain silage treated with different doses of *Lactobacillus buchneri* or a combination of *Lactobacillus plantarum* and *Pediococcus acidilactici*. *Int. J. Dairy Sci*. **2016**, *101*, 4158–4167.
218. Granado-Serrano, A.B.; Martín-Garí, M.; Sánchez, V.; Riart Solans, M.; Berdún, R.; Ludwig, I.A.; Rubió, L.; Vilaprinyó, E.; Portero-Otín, M.; Serrano, J.C.E. Faecal bacterial and short-chain fatty acids signature in hypercholesterolemia. *Sci. Rep.* **2019**, *9*, 1–13.
219. Spadafora, N.D.; Cammarisano, L.; Rogers, H.J.; Müller, C.T. Using volatile organic compounds to monitor shelf-life in rocket salad. *Acta Hortic.* **2018**, *1194*, 1299–1306.
